# Generation and Integrated Analysis of Advanced Patient‐Derived Orthoxenograft Models (PDOX) for the Rational Assessment of Targeted Therapies in Endometrial Cancer

**DOI:** 10.1002/advs.202204211

**Published:** 2022-11-14

**Authors:** Laura Devis‐Jauregui, August Vidal, Laura Plata‐Peña, Maria Santacana, Sandra García‐Mulero, Nuria Bonifaci, Eulàlia Noguera‐Delgado, Nuria Ruiz, Marta Gil, Eduard Dorca, Francisco J. Llobet, Laura Coll‐Iglesias, Katja Gassner, Maria Martinez‐Iniesta, Ruth Rodriguez‐Barrueco, Marc Barahona, Lola Marti, Francesc Viñals, Jordi Ponce, Rebeca Sanz‐Pamplona, Josep M. Piulats, Ana Vivancos, Xavier Matias‐Guiu, Alberto Villanueva, David Llobet‐Navas

**Affiliations:** ^1^ Molecular Mechanisms and Experimental Therapy in Oncology‐Oncobell Program Bellvitge Biomedical Research Institute (IDIBELL) L'Hospitalet de Llobregat Barcelona 08908 Spain; ^2^ Department of Pathology University Hospital of Bellvitge Bellvitge Biomedical Research Institute (IDIBELL) L'Hospitalet de Llobregat Barcelona 08908 Spain; ^3^ Scientific and Technical Service of Immunohistochemistry Lleida Institute for Biomedical Research Dr. Pifarré Foundation IRBLleida Hospital Universitari Arnau de Vilanova Lleida 25198 Spain; ^4^ Department of Pathology Universitat de Lleida/Institut de Recerca Biomèdica de Lleida/Hospital Universitari Arnau de Vilanova Lleida 25198 Spain; ^5^ Unit of Biomarkers and Susceptibility Oncology Data Analytics Program (ODAP) Catalan Institute of Oncology (ICO) Oncobell Program Bellvitge Biomedical Research Institute (IDIBELL) and CIBERESP L'Hospitalet de Llobregat Barcelona 08908 Spain; ^6^ Department of Basic Sciences Universitat de Lleida/Institut de Recerca Biomèdica de Lleida Edifici Biomedicina I, Lab 2.4 Lleida 25198 Spain; ^7^ Centro de Investigación Biomédica en Red de Cáncer (CIBERONC) Instituto de Salud Carlos III (ISCIII) Madrid 28029 Spain; ^8^ Catalan Institute of Oncology (ICO) Bellvitge Biomedical Research Institute (IDIBELL) L'Hospitalet de Llobregat Barcelona Barcelona 08908 Spain; ^9^ Chemoresistance and Predictive Factors Group Program Against Cancer Therapeutic Resistance (ProCURE) Catalan Institute of Oncology (ICO) Bellvitge Biomedical Research Institute (IDIBELL) L'Hospitalet de Llobregat Barcelona 08908 Spain; ^10^ Anatomy Unit Department of Pathology and Experimental Therapy School of Medicine University of Barcelona (UB) L'Hospitalet de Llobregat Barcelona 08907 Spain; ^11^ Department of Gynaecology University Hospital of Bellvitge Bellvitge Biomedical Research Institute (IDIBELL) L'Hospitalet de Llobregat Barcelona 08908 Spain; ^12^ Department of Physiological Sciences School of Medicine University of Barcelona (UB) L'Hospitalet de Llobregat Barcelona 08907 Spain; ^13^ Cancer Genomics Group Vall d'Hebron Institute of Oncology (VHIO) Barcelona 08035 Spain; ^14^ Xenopat S.L. Parc Cientific de Barcelona (PCB) Barcelona 08028 Spain

**Keywords:** endometrial cancer, HER2, patient‐derived orthoxenografts, PDOX, targeted therapies

## Abstract

Clinical management of endometrial cancer (EC) is handicapped by the limited availability of second line treatments and bona fide molecular biomarkers to predict recurrence. These limitations have hampered the treatment of these patients, whose survival rates have not improved over the last four decades. The advent of coordinated studies such as The Cancer Genome Atlas Uterine Corpus Endometrial Carcinoma (TCGA_UCEC) has partially solved this issue, but the lack of proper experimental systems still represents a bottleneck that precludes translational studies from successful clinical testing in EC patients. Within this context, the first study reporting the generation of a collection of endometrioid‐EC‐patient‐derived orthoxenograft (PDOX) mouse models is presented that is believed to overcome these experimental constraints and pave the way toward state‐of‐the‐art precision medicine in EC. The collection of primary tumors and derived PDOXs is characterized through an integrative approach based on transcriptomics, mutational profiles, and morphological analysis; and it is demonstrated that EC tumors engrafted in the mouse uterus retain the main molecular and morphological features from analogous tumor donors. Finally, the molecular properties of these tumors are harnessed to assess the therapeutic potential of trastuzumab, a human epidermal growth factor receptor 2 (HER2) inhibitor with growing interest in EC, using patient‐derived organotypic multicellular tumor spheroids and in vivo experiments.

## Graphical abstract

A new system to advance treatment options for endometrial cancer patients is reported. This model is based on the establishment of patient avatars through orthotopic engraftments in the mouse uterus. The morphological, transcriptomic, and DNA mutation analyses evidence that primary tumors and avatars exhibit multiscale resemblance and demonstrate that human epidermal growth factor receptor 2, HER2^R678Q^‐mutated tumors can be targeted in vivo with trastuzumab.

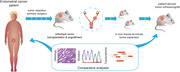

## 1 Introduction

Epidemiological data from cancer registry programs indicate that, between 1975 and 2014, 5‐year survival rates have improved for the most common cancers except for cervix and endometrial cancer, greatly evidencing the need to find more efficient therapeutic options for these patients.^[^ ^1^ ^]^ Endometrial cancer (EC) is the most common gynecological cancer in developed countries, standing as the 4th most frequent and the 6th most deadliest type of tumor in women in the Unites States.^[^ ^2^ ^]^ Not surprisingly, the number of new cases and deaths for EC in 2020 worldwide were estimated as 417 367 and 97 370, respectively,^[^ ^3^ ^]^ and current trends indicate that EC incidence and death rates are further increasing in part due to the rise of predisposing risk factors, especially those related to metabolic syndrome such as obesity.^[^ ^2^, ^4^, ^5^ ^]^

In general, the prognosis and clinical course for most EC patients is favorable. Accordingly, the majority of them (≈75%) present localized early disease symptomatology, such as vaginal bleeding or discharge, which favors early stage diagnosis and curative surgery leading to 5‐year overall survival rates of 80–95%. Nevertheless, around 15–20% of EC patients will experience tumor recurrence, largely impacting treatment options and patient survival.^[^ ^6^, ^7^ ^]^ Adjuvant therapeutic options for recurred, as well as for advanced or metastatic, EC patients are very limited and encompass the delivery of standard chemotherapy, mainly platinum or paclitaxel. However, treatment resistance is unfortunately common and only 10–15% of all EC patients will benefit from it.^[^ ^8^ ^]^ This is worsened by the limited availability of established second line treatments, validated patient selection protocols, and licensed targeted drugs for EC.^[^ ^9^ ^]^ Consequently, the prognosis of patients with recurred or metastatic EC is still poor with median survival rates of less than one year and median progression free survival rates of four months.^[^ ^10^ ^]^ Altogether, these data indicate that EC patients presenting refractory or systemic disease represent nowadays a therapeutic challenge, which is aggravated by the limited response rates associated to current adjuvant chemotherapy.

In the last years, patient‐derived xenograft (PDX) models have emerged as powerful tools in cancer research.^[^ ^11^, ^12^, ^13^ ^]^ PDX models have been revealed as extraordinary accurate preclinical models with a high predictive drug‐response value^[^ ^14^ ^]^ by outperforming regular in vitro cell line models and cell‐line‐derived xenografts because of their ability to retain, among others, essential tumor traits from the original tissue donor such as tumor heterogeneity, molecular fingerprints (genetic and transcriptomic), tissue architecture, spatial distribution, and cell‐to‐cell interactions. Not surprisingly, a high correlation between PDX and patient response has been observed when PDXs were included in clinical or coclinical trials,^[^ ^11^, ^15^, ^16^ ^]^ offering the potential to increase the success rate of compound approval after assessment in patient clinical trials. Altogether, this analytical strategy has become an innovative resource to fine‐tune treatment decisions in the future and to accelerate drug development and personalized medicine by complementing pathology and molecular analysis.

Herein, we expand our knowledge on the therapeutic potential and value of this experimental system by generating and profiling a collection of patient‐derived orthoxenografts (PDOXs) from 15 endometrioid endometrial cancer patients. We provide evidence that EC tissue orthotopic engraftment in mice retains most of the histological, genetic, and transcriptomic features of patient donors. Moreover, by leveraging this information, we have assessed the therapeutic utility of trastuzumab (a human epidermal growth factor receptor 2, HER2 inhibitor) in HER2‐mutated tumors, collectively demonstrating the relevance of PDOX models to explore new therapeutic avenues for EC patients. Finally, we also show that PDOXs are dynamic entities and that, as such, can exhibit intriguing evolutionary features such as dedifferentiation as they progress in vivo.

## 2 Results

### 2.1 Patient Description and Establishment of PDOXs

**Figure 1:**
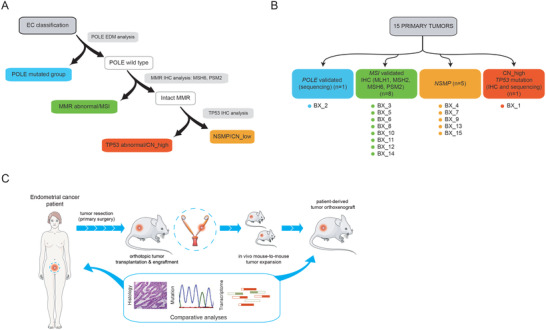
Initial patient sample analysis, characterization, and strategy used for PDOX generation. A) Schematic overview of standard procedures during EC sample pathologic assessment. In a first triage, samples are assessed for the presence of POLE exonuclease domain mutations (EDM) at exons 9 and 13 by targeted DNA sequencing. Wild type POLE EC samples are then analyzed by immunohistochemistry to assess the levels of mismatch repair genes (MMR) as a diagnostic surrogate of the MSI molecular subtype. MMR proficient tumors are then subjected to an analysis by immunohistochemistry of *TP53* protein levels. As in the case of MMR genes, high levels of *TP53* are used as a nonperfect surrogate marker of *TP53*‐mutated EC and CN‐high tumors. Tumors that do not fall into any category are collectively called NSMP (nonspecific molecular profile, low copy number, microsatellite stable (MSS)). B) Schematic overview of the TCGA_UCEC‐based molecular classification of primary tumors used in this study. C) Establishment of PDOX models for preclinical treatment in endometrial cancer overview. Resected fragments of endometrial cancer tumor were orthotopically injected into athymic mice uteruses. To test the reproducibility of patient features in the mouse model, primary tumors and established PDOXs were characterized by immunohistochemistry, RNA‐seq, and mutational analyses. Parts of the Figure 1c were drawn by using pictures from Servier Medical Art. Servier Medical Art by Servier is licensed under a Creative Commons Attribution 3.0 Unported License (https://creativecommons.org/licenses/by/3.0/).

In our study, we prospectively recruited 15 patients diagnosed with EC of endometrioid morphology, the most frequent EC histological subtype, who underwent primary surgical resection at the Hospital Universitari de Bellvitge/Bellvitge Hospital (HUB) (Tables S1 and S2, Supporting Information). Detailed histological analysis determined that 14 patients (93.3%) presented endometroid endometrial cancer (EEC) with the exception of 1 patient (6.7%) who presented a mixed endometroid–serous carcinoma (90% endometrioid; 10% serous). Most patients were diagnosed at stage I (12/15; 80%) and presented well or moderately differentiated tumors (10/15; 66.7%). Seven patients (46.7%) did not receive any additional treatment after primary surgery and 7/15 patients (46.7%) were treated with brachytherapy +/− radiotherapy. Two patients (13.3%) presented recurrence and 1 of these patients died of the disease (6.7%). Median follow‐up time was 59.27 months (47.30–71.23 months). Among the two patients who suffered recurrence, patient 5 (biopsy BX5) was diagnosed at stage IB of the disease with grade 3 (G3) EEC and, in addition to surgery, received a combination of radiotherapy and brachytherapy as adjuvant treatment. Patient 9 (BX9) was diagnosed at stage IA G1 and did not receive adjuvant treatment. Following the HUB standard pathological analysis based on morphological features and TCGA molecular EC biomarkers (**Figure** **1**A), primary tumors were classified into polymerase epsilon mutated (POLE‐mutated), tumors presenting microsatellite instability (MSI), nonspecific molecular profile; low copy number or microsatellite stable (MSS) (NSMP), and *TP53*‐mutated (serous‐like, high copy number) (Figure 1B).

To generate the PDOX models, nonnecrotic tissue fragments from EC resected tumors were stored in supplemented medium and immediately implanted into recipient uteruses of immunodeficient mouse females in order to preserve cell viability (Figure 1C). Tumor growth was monitored twice per week with an engraftment average time (engraftment to early passage P1 tumors) of 102.47 days (84.57–120.36). Once the PDOX models were established, we performed histological, mutational, and genome‐wide transcriptional analyses between the primary tissue and the tumors from the mouse avatar at P1 to assess their main morphological, genetic, and transcriptomic characteristics.

### 2.2 PDOX Models Retain the Main Histological and Molecular Features of Primary Tumors

**Figure 2:**
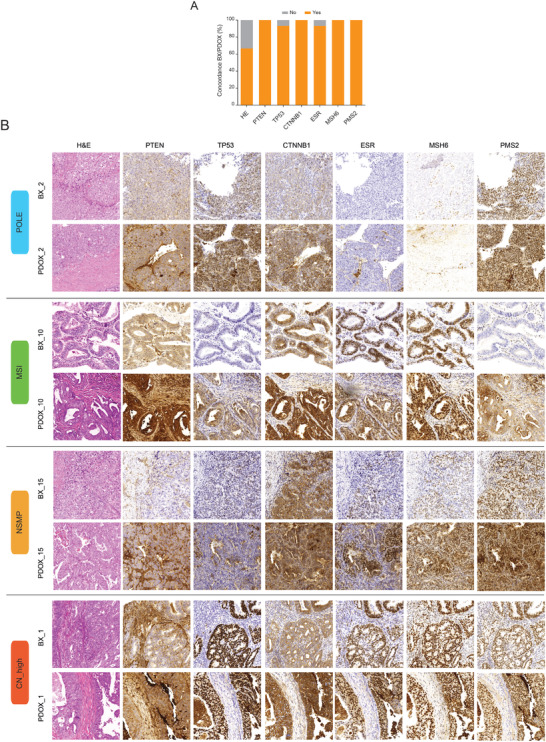
Morphological and immunohistochemical correlation between primary tumors (BXs) and PDOXs. A) Percentage of cases displaying consistent positive correlations between paired BX–PDOX after morphologic analysis by hematoxylin and eosin (HE) and after immunohistochemical detection of canonical EC biomarkers (*PTEN*, *TP53*, *CTNNB1*, *ESR*, *MSH6*, and *PMS2*). B) Representative examples of fully correlated BX–PDOX pairs. Images illustrate HE stainings and detection by immunohistochemistry of *PTEN*, *TP53*, *CTNNB1*, *ESR*, *MSH6*, and *PMS2* between BX and PDOX. BX1 (CN‐high), BX2 (POLE‐mutated), BX10 (MSI), and BX15 (MSS).

**Figure 3:**
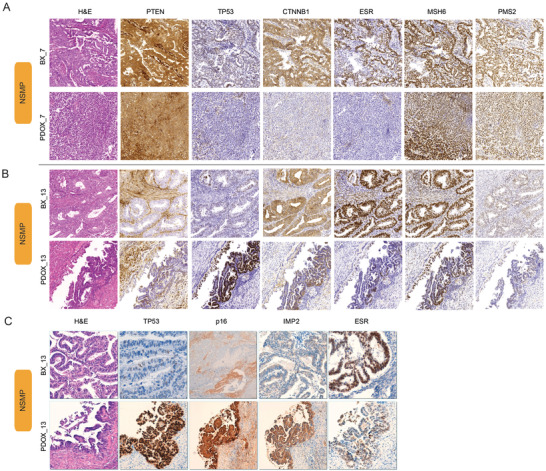
Detection of immunohistochemical differences between primary tumors (BXs) and PDOXs. Representative images presenting two PDOX cases displaying immunohistochemical tumor features that were not detected in the original primary tumor: A) pair BX7–PDOX7 and B) pair BX13–PDOX13. Images show a differential *ESR* expression in PDOX7, while PDOX13 presented areas of serous carcinoma and abnormal *TP53* overexpression that were not observed in the primary tumor. C) HE and immunostaining images for *TP53*, *p16*, *IMP2*, and *ESR* in PDOX13.

Hematoxylin eosin (HE) staining, as well as phosphatase and tensin homolog (*PTEN)*, *TP53*, *CTNNB1*, estrogen receptor (*ESR)*, mutS homolog 6 (*MSH6)*, *PMS2* immunostainings were performed in both primary tumors and PDOXs. Overall, PDOXs presented a high degree of concordance at histological level (≈70% with their primary tumors) and molecular biomarker expression (**Figure** **2**A,B and Figures S1 and S2 and Table S3 (Supporting Information)). Full concordance was detected in *PTEN*, nuclear *CTNNB1*, *MSH6*, and *PMS2* immunostainings between BXs and PDOXs. *ESR* and *TP53* showed a good correlation (93.33%) between BXs and PDOXs. In the case of *TP53*, and in line with our initial assessment (Figure 1B), BX1 and its derived PDOX1 presented an abnormal staining by immunohistochemistry (a surrogate of the copy number high, CN_high molecular EC subgroup) and were therefore classified within the *TP53*‐mutated (serous‐like, high copy number) group. Of note, 2 PDOX models (PDOX7 and PDOX13) displayed differences in the expression of certain molecular biomarkers compared to their primary tumor (BX). This is evidenced in PDOX7 by an increase in tumor grade from G1 to G3 with undifferentiated tumor areas and a concomitant loss in *ESR* expression (**Figure** **3**A). This result is in agreement with the histologic difference observed in this model (G3 and presence of undifferentiated tumor areas) (Table S3, Supporting Information). On the other hand, PDOX13 presented de novo serous carcinoma areas, as observed in the HE, characterized by an abnormal *TP53*, *p16*, and *IMP2* stainings that were not observed in the initial tumor biopsy (Figure 3B,C).

### 2.3 Transcriptomic Characterization of Tumors and PDOX Models

**Figure 4:**
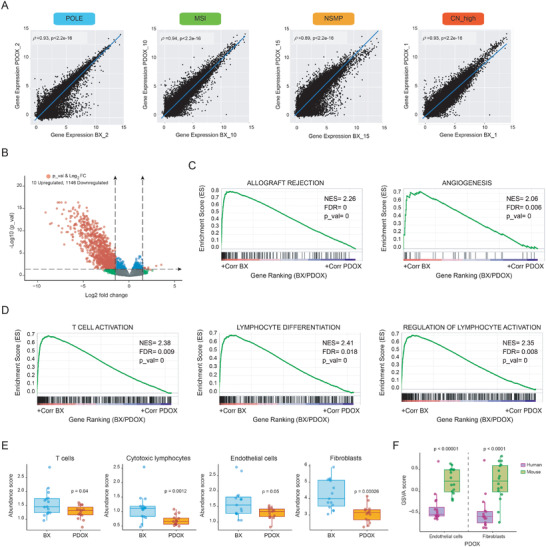
Transcriptomic analysis of BX and PDOX tumor pairs. A) Total RNA sequencing for the 15 pairs of BX/PDOX tumors was conducted and computationally analyzed. BX and PDOX pairs correlated extremely well at transcriptomic level. Spearman's correlation (rho) *ρ* values and *p* values indicate the strength and significance of the correlations, respectively. B) Volcano plot depicting differentially expressed genes between PDOXs and primary tumors (*p* value < 0.05 and logFC > abs(1.5)). A total of 1146 genes were significantly reduced and only 10 were significantly increased in the PDOX samples. C) Significantly enriched gene annotations by GSEA from gene ontology biological pathways (GO) and D) hallmarks indicative of progressive changes in the human tumor microenvironment. E) Transcriptional inference of stroma and immune infiltrates using the MCP‐counter reveals a decrease in the abundance of representative signatures of human T cells, cytotoxic lymphocytes, endothelial cells, and fibroblasts in the PDOX. F) GSVA analysis of the representation in fibroblastic and endothelial gene annotations from mouse and human specific reads.

To further explore the molecular similarity between PDOX and primary tumors, we analyzed their transcriptional landscape by RNA_sequencing. After curated filtering of reads from mouse origin (please see the Experimental Section for further technical details), a high degree of resemblance at transcriptional level was correlated across the 15 paired samples (BX and PDOX) (Spearman's *ρ* = 0.89–0.95) (**Figure** **4**A and Figure S3 (Supporting Information)). We also performed differential expression analysis to identify differentially expressed genes (DEGs) between primary tumors and PDOXs (Figure 4B). Interestingly, we found 1146 downregulated and 10 upregulated genes in PDOXs compared with the corresponding primary samples (Table S4, Supporting Information), reflecting potential biological differences between both tissues. In this line, unbiased gene set enrichment analysis (GSEA) analysis^[^ ^17^ ^]^ using the archived gene ontology biological process (Figure 4C) and hallmark gene annotations (Figure 4D), revealed that genes with enriched expression in primary tumors were mostly associated with human tumor microenvironment (e.g., genes involved in transplant rejection alloimmune response, immune cell differentiation and activation, etc.), which is consistent with the profound changes in the human tumor‐associated stroma (cancer‐associated fibroblasts, immune cells, extracellular matrix, endothelial cells, etc.) and its replacement by the murine stromal components commonly seen in mouse xenografts.^[^ ^12^ ^]^ To support this, we inferred changes in the human infiltrating stromal compartment by employing the microenvironment cell populations‐counter (MCP‐counter) method, which has one of the highest specificities in quantifying the absolute abundance of multiple stromal cell compartments from transcriptomic data^[^ ^18^ ^]^ (Figure 4E). As expected, we identified significant changes in immune and stromal infiltrates across the 15 BX and PDOX samples, including a decrease in human T cells and cytotoxic lymphocytes, and a reduction in human endothelial cells and fibroblasts in the PDOXs with respect to BXs which, collectively, indicate that human stromal cells are progressively being replaced by murine cells upon engraftment in the mouse uterus. This was also supported by our gene set variation analysis (GSVA)^[^ ^19^ ^]^ of the fibroblastic and endothelial content when using gene expression from mouse and human origin in PDOX samples (Figure 4F) which, collectively, evidence a significant enrichment in mouse stromal cells within the human orthoxenograft. Altogether, our results indicate that PDOX tumors exhibit not only a notable retention in histological and biomarker features, but also an excellent correlation with BX samples at transcriptomic level. Also, consistent with previous observations, our data point at important changes in the tumor microenvironment.

### 2.4 Mutational Profiling of Primary Tumors and PDOX Models for the Rational Assessment of Targeted Therapies

**Figure 5:**
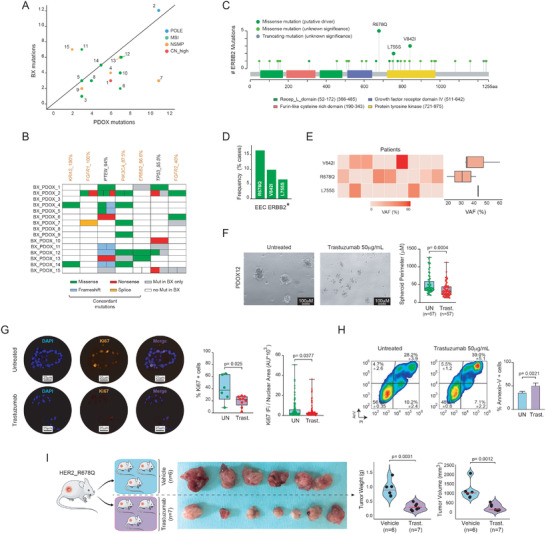
Mutational characterization of BX and PDOXs and effects of trastuzumab in vivo. A) Dot plot representing the correlation between the number of mutations in primary tumors and PDOXs. B) Schematic representation of common somatic mutations for several EC‐relevant genes between primary tumors (BX) and their respective PDOX. Gene mutations are ranked from left to right according to percentage of mutations presenting concordance in relation to all mutations for a given gene in BX (i.e., percentage of mutations for a given gene that are maintained in PDOX models). Missense, frameshift, and nonsense mutations were detected. Genes highlighted in orange represent druggable genes. C) Lollipop plot and analysis of the HER2 mutational landscape in endometrioid EC. Analysis has been performed on HER2‐mutated tumors (*n* = 31), using the TCGA_ucec dataset. D) Bar graph showing that *ERBB2^R678Q^* mutations account for >15% of HER2 mutations in EC. E) Heatmap and boxplots depicting the variant allele frequency (VAF) for the three most frequent HER2 mutations in EC: V842I, R678Q, and L755S. F) Generation of tumor spheroids. Left, bright field representative analysis of untreated and trastuzumab‐treated *ERBB2^R678Q^* spheres after 24 h. Right, quantification of spheroid perimeters. Statistical significance was determined by paired Student's *t*‐test. G) Analysis of *Ki67* expression levels by immunofluorescence (IF) in untreated and trastuzumab‐treated spheroids after 24 h. Left, representative IF images. Right, quantification of percentage of *Ki67*‐positive cells and alternative quantification after normalizing *Ki67* intensity (arbitrary units) to nuclear area, as previously reported.^[^ ^76^ ^]^ H) Analysis of Annexin‐V staining by flow cytometry on untreated and 24 h treated spheroids with trastuzumab. I) In vivo assessment of trastuzumab anticancer effects on PDOX12, *ERBB2^R678Q^*‐mutated, mouse model. Females were treated twice per week with 4 µg kg^−1^ of trastuzumab i.p. for a period for four weeks. Quantification of tumor weight and volume was performed at the end of the experiment. Statistical significance in in vivo experimentation was assessed by the Mann–Whitney test. Parts of the Figure 5I were drawn by using pictures from Servier Medical Art. Servier Medical Art by Servier is licensed under a Creative Commons Attribution 3.0 Unported License (https://creativecommons.org/licenses/by/3.0/).

Our data indicate that PDOXs could be harnessed to explore novel precision medicine compounds in EC based on distinctive BX–PDOX tumor molecular fingerprints. To this end, we resolved to identify relevant BX analogous actionable or predictive treatment response mutations in our PDOXs. Hence, we performed DNA targeted next generation sequencing in EC and PDOX tumors using a comprehensive cancer gene panel that includes 57 cancer‐associated genes (Table S5, Supporting Information). A total of 68 and 85 mutations were detected in primary tumors and in PDOXs, respectively (Table S6, Supporting Information). Noteworthy, PDOXs 1, 3, 4, 6, 7, 8, 9, 10, 12, and 13 presented a slight increase in the number of mutations compared to their original primary tumor (**Figure** **5**A). Common and fully concordant mutations shared by primary and PDOX cases (*n* = 48), representing the 70.59% and 56.47% of all mutations found in primary tumors and PDOX, respectively, encompassed several of the most relevant genes involved in EC development and progression, including *PTEN*, *PIK3CA*, *TP53*, and *KRAS* (Figure 5B). Importantly, full coincidental mutations were also found in other genes with a less clear role in the development and/or progression of EC but with potential therapeutic interest (e.g., fibroblast growth factor receptor 1 (*FGFR1)*, erb‐b2 receptor tyrosine kinase 2 (*ERBB2)*, etc.). Altogether, these data suggest that PDOXs are able to retain the majority of assessed mutations (>50%) detected in primary tumors and that this information could be leveraged to assess personalized treatments.

### 2.5 Evaluation of Trastuzumab as a Potential Precision Medicine Agent for Endometrioid Endometrial Cancer

Based on all the aforementioned, we focused on *ERBB2* for further analysis. *ERBB2* (hereafter termed HER2) is a membrane tyrosine kinase receptor that belongs to a family of four receptors (HER1–4) involved in cell growth, survival, and proliferation.^[^ ^20^, ^21^ ^]^ HER2 is a well‐known oncogene, and it is found overexpressed in multiple types of tumors owing to, mainly, copy number alteration (CNA) events (amplifications).^[^ ^22^ ^]^ In EC, although the reported frequencies are variable, it is well established that HER2 is also found amplified and/or overexpressed in tumors with high grade histology (≈10–80%),^[^ ^22^ ^]^ and that this genetic alteration is frequently associated to *TP53* mutations.^[^ ^23^ ^]^ Importantly, HER2 mutations have also been found in EC, albeit with lower frequency (≈2–3%).^[^ ^22^ ^]^ Despite the clinical relevance of HER2 amplification/overexpression and mutational status in EC is still a matter of intense debate, HER2 has attracted attention as a putative therapeutic target. Indeed, not only the inhibition of HER2 has shown potent anticancer effects in vitro and in vivo, alone or in combination,^[^ ^24^, ^25^ ^]^ but also has demonstrated encouraging results in clinical trials. It must be noted, however, that with the exception of few studies that encompass a reduced number of EC patients with HER2 mutations,^[^ ^26^, ^27^, ^28^ ^]^ most of the existing clinical trials assessing anti‐HER2 agents in EC are conducted on patients presenting amplified or overexpressed *ERBB2*. For instance, existing evidence from the NCT01367002 study indicates that combination of trastuzumab (an anti‐HER2 antibody) with adjuvant paclitaxel–carboplatin increases progression‐free survival rates when compared to the same combination without trastuzumab in advanced/recurrent endometrial cancer patients with HER overexpression.^[^ ^29^, ^30^ ^]^ More recently, results from the Targeted Agent Profiling and Utilization Registry study (NCT02693535) also demonstrate the therapeutic benefit of the combination of trastuzumab with pertuzumab mostly in amplified/overexpressed *ERBB2*/*ERBB3* uterine tumors.^[^ ^26^ ^]^ In this scenario, we decided to explore the potential use of our PDOX models to test novel therapeutic strategies by assessing the anticancer efficacy of trastuzumab in HER2‐mutated tumors. To address this, we selected the PDOX12, which harbors a BX12 matched HER2 mutation at the juxtamembrane domain of HER2 (HER2^R678Q^). HER2^R678Q^ represents a suitable experimental context, since this mutation has been reported as the most recurrent *ERBB2* juxtamembrane domain mutation after massive exome sequencing analysis from ≈111 000 cancer patients, representing ≈400 cancer types, including endometrial cancer.^[^ ^31^ ^]^ Importantly, HER2^R678Q^ is regarded as a hotspot mutation^[^ ^32^, ^33^ ^]^ (https://www.cancerhotspots.org) and a driver oncogenic event that confers gain of function features by increasing HER2 phosphorylation and downstream signaling in bladder cancer and breast epithelial cells, where it accelerates acinar structure formation and cell survival.^[^ ^31^, ^34^ ^]^ Finally, HER2^R678Q^ has been shown to confer sensitivity to trastuzumab in vitro.^[^ ^31^, ^35^ ^]^ In agreement, our analysis of the HER2 mutational landscape across >10 000 tumors using the cBioportal platform^[^ ^36^ ^]^ confirmed that HER2^R678Q^ is one of the most prevalent mutations in cancer (Figure S4A, Supporting Information). Importantly, when we categorized *ERBB2^R678Q^* mutations according to tumor type, we found that >30% of *ERBB2^R678Q^* mutations cluster in the endometrial cancer type (Figure S4B, Supporting Information). Collectively, our data indicate not only that *ERBB2^R678Q^* is one of the most frequent *ERBB2* mutations in cancer globally, but also indicate for the first time that *ERBB2^R678Q^* is particularly enriched in endometrial cancer. Additional analysis using the TCGA_uterine corpus endometrial cancer (ucec) dataset further demonstrates that this mutation is the most frequent in endometrioid EC among all *ERBB2* mutations (Figure 5C–E).

Next, to explore the effects of trastuzumab, we initially assessed its activity using ex vivo patient‐derived organotypic multicellular tumor spheroids.^[^ ^37^ ^]^ Despite tumor spheroids do not fully mimic the 3D conditions of living organs (e.g., vascularization, tissue‐tissue interfaces, mechanical pressures or tumor microenvironment),^[^ ^38^ ^]^ they are more informative than traditional 2D cell cultures as tumor spheroids more closely recapitulate certain tissular architecture features (e.g., cell to cell interactions), and physiological properties of analogous in vivo tumors.^[^ ^39^ ^]^ Hence, spheroids from BX12 were generated and exposed to trastuzumab following previously reported experimental conditions.^[^ ^40^ ^]^ As hypothesized, trastuzumab impinged a dramatic effect on cell fitness, characterized by a reduction in tumor spheroid perimeter (Figure 5F) and decreased expression of the cell proliferation marker *Ki67* (Figure 5G). Interestingly, treatment with trastuzumab also triggered a notable cytotoxic effect, as measured by apoptosis induction (Figure 5H). On the basis of these lines of evidence, we resolved to assess the antitumor effect of trastuzumab in vivo using the *ERBB2^R678Q^*‐mutated PDOX12. To this end, the colony of immunosuppressed mouse females harboring engrafted BX12 tumors in the uterus was expanded and animals were randomized before treatment. As shown in Figure 5I, treatment with trastuzumab significantly delayed tumor growth, as evidenced by a remarkable reduction in tumor weight and volume. Altogether, our data demonstrate not only that PDOXs derived from primary EC biopsies maintain essential morphologic and molecular features, but that its genetic profiling can reveal unexpected tumor vulnerabilities that can be used to advance personalized treatments in endometrial cancer.

## 3 Discussion

Prior to 1930s–1940s, uterine cervix and uterine corpus cancers were considered a single epidemiological entity (i.e., uterine cancer) that constituted the major cause of biased cancer‐related deaths against women in comparison to men.^[^ ^41^ ^]^ The implementation of screenings for the early detection of cervix cancer reduced uterine cancer death rates by 80% and represents to date one of the major achievements in cancer prevention. Unfortunately, in the case of endometrial (or uterine corpus) cancer, the technological advances accomplished in the cancer arena (e.g., robotic surgery, imaging, or pathological analysis) have been complemented with a scarce increase in therapeutic opportunities, especially in the area of precision medicine or targeted therapies.

Up to present, Bokhman's dualistic model is the most widely used strategy to classify EC and one of the pillars aiding treatment decisions.^[^ ^42^ ^]^ The model is based on clinical and pathological features and broadly classifies ECs into type I or endometroid (EEC) and type II or nonendometroid (NEEC) tumors. Type I tumors are low grade, estrogen‐dependent tumors that predominate in pre‐ or perimenopausal women and represent ≈75% of all diagnosed ECs. On the other hand, type II ECs are estrogen‐independent tumors that mainly develop in older women and present a poor clinical outcome. They include mostly serous and clear cell histological subtypes.^[^ ^43^ ^]^ Currently, the mainstay approach guiding treatment decisions in endometrial cancer is based on histological risk criteria that include histological subtype, grade, lymphovascular invasion, and tumor stage,^[^ ^4^ ^]^ being the International Federation of Gynecology and Obstetrics (FIGO) staging the single strongest prognostic parameter for EC patients. However, these assessments are often challenging and the difficulties that pathologists sometimes encounter to reach a consensus in the analysis and interpretation of these criteria has hindered the development of standardized guidelines to avoid patient under‐ or overtreatment. In this line, the discovery in 2013 by the TCGA consortia of a new classification based on molecular profiling of EC has shed light onto the different genomic subtypes of EC, offering the potential to improve EC patient stratification postsurgery by supporting expert gynecopathologist analyses.^[^ ^44^ ^]^ Importantly, the European Society of Gynaecological Oncology (ESGO), the European SocieTy for Radiotherapy and Oncology (ESTRO), and the European Society of Pathology (ESP) ESGO‐ESTRO‐ESP guidelines, which represent the main reference on evidence‐based recommendations for the improvement of care for women with EC, have incorporated the molecular classification as an essential pilar on EC diagnosis and treatment.^[^ ^45^ ^]^ Moreover, the TCGA_UCEC molecular classification has provided abundant genetic and epigenetic information with the potential to improve tailored treatments. Results of this study show that unsupervised hierarchical clustering of somatic copy number alterations (SNCAs) and exome sequence analysis categorized EC tumors in four groups: group‐1 or POLE‐ultramutated (EEC with mutations in POLE, showing high mutation rates, with low SNCA and associated with good prognosis), group‐2 or MSI (EEC with microsatellite instability (hypermutated) and low SNCA), and group‐3 (EEC exhibiting microsatellite stability and amplification of 1q) showing similar progression‐free survival rates. Finally, group‐4 (serous carcinomas (NEEC) and 12% of EEC, particularly grade 3 tumors) is characterized by presenting high SNCA, *TP53* mutations, and worse prognosis. One of the most interesting results is the identification of molecular similarities between group‐4 and high grade serous ovarian carcinomas and basal‐like breast tumors, outlining a potential shared treatment strategy.

Current treatments for EC patients do not substantially differ from those that have been implemented for decades, and little advances have been achieved in this underfunded and often overlooked area of research.^[^ ^46^ ^]^ In fact, it is commonly accepted that traditional chemotherapeutic regimes are less effective in EC when compared with cancers of other organs, greatly emphasizing the urgent need to identify new molecular targets and treatment options. The discovery and emergence of the TCGA_ucec‐based genomic information is thus expected to hasten the discovery of new molecular targets and strategies to combat EC in the future. Some of the most promising targeted therapies currently being assessed include those that involve the inhibition of the phosphatidylinositol‐3‐kinase(*PI3K)*–mammalian target of rapamycin (*mTOR)*, PI3K‐mTOR pathway, *FGFR2*, or *RAS*–mitogen activated protein kinase (*MAPK)* while, most recently, immunotherapy has become the primary approach in many trials.^[^ ^9^ ^]^ In this line, the approval in May 2017 by the U.S. Food and Drug Administration (FDA) of pembrolizumab for the treatment of refractory microsatellite (MS) instable‐high/mismatch‐repair (MMR)‐deficient EC,^[^ ^47^ ^]^ the approval in 2019 of pembrolizumab combined with lenvatinib for the treatment of progressed MMR proficient/MS stable EC,^[^ ^48^ ^]^ and the recent approval of dostarlimab for the treatment of advanced MSI endometrial cancer^[^ ^49^, ^50^ ^]^ have significantly improved the clinical management of this disease. However, despite our knowledge of EC has increased considerably over the last decade, EC treatment still lags far behind other types of malignancies in the area of precision medicine. The lack of proper experimental model systems at the preclinical triage still represents a major impediment precluding success of anticancer drug assessment in clinical trials. Consequently, the future implementation of novel, more efficient and less toxic treatment options in EC must stem from accelerated and improved transferability success of “from bench to bedside” studies, one of the major bottlenecks that hamper therapeutic compound approval. In this line, despite several novel therapies are supported by conventional in vitro*/*in vivo preclinical evidences, the efficacy to translate these findings into the clinic has remained extremely low^[^ ^51^, ^52^ ^]^ and successful drugs in preclinical testing often fail upon reaching phase III clinical trials.^[^ ^53^ ^]^ Accordingly, only 5% of compounds with validated preclinical anticancer properties are approved by the FDA for clinical implementation.^[^ ^54^ ^]^ The reasons behind these low success rates could lie in the inability of commonly established preclinical models to recapitulate the complexity of a patient tumor,^[^ ^52^ ^]^ the discrepancies observed, over time and with increased cell passaging, between the primary tumor and its derived cell line,^[^ ^55^ ^]^ or the limited value of conventional cell lines to predict treatment response.^[^ ^52^ ^]^

In a recent pilot study from our laboratory, we reported the anticancer properties of the autophagy inhibitor chloroquine combined with sorafenib in three PDOX EC models.^[^ ^56^ ^]^ The robustness of the assay combined with an evident translational potential prompted us to further characterize this experimental system by expanding this methodology to the most frequent EC molecular subtypes and by interrogating primary tumor‐PDOX resemblance through genetic and transcriptomic approaches. To achieve this and to overcome all the aforementioned experimental limitations, we have expanded our knowledge on EC by generating direct orthotopic xenograft models from 15 EC patients. Although the generation of panels of >10 subcutaneous and/or subrenal EC PDX have been described in the recent years,^[^ ^57^, ^58^, ^59^, ^60^, ^61^ ^]^ to the best of our knowledge, no other attempts to systematically implant and profile at a genome‐wide scale matched primary tumor and orthotopically inserted primary‐derived EC tissue in mice have been reported. Importantly, we provide abundant data demonstrating that implantation and engraftment of primary EC tissue in the mouse uterus can regenerate the human tumor in mice. In this regard, it is well recognized that the site of implantation (heterotopic vs orthotopic) can influence not only tumor growth, desmoplasia, vascularization, stromal infiltration, and response to antineoplastic treatments but also dramatically affect the metastatic behavior, being heterotopic PDX less able to better recapitulate the tumor progression observed in cancer patients.^[^ ^62^, ^63^, ^64^, ^65^, ^66^ ^]^ Altogether, our experimental system aims at mirroring as close as possible the patient's tumor by preserving the natural setting of the disease through an orthotopic implantation.

Our data indicate that PDOX tumors recapitulate the main morphological, molecular, and transcriptomic features of the primary tumor, can be expanded in recipient mice (avatars), and can be used to explore the therapeutic potential of targeted therapeutic compounds. Accordingly, in this study, we performed a successful in vivo analysis using trastuzumab, an inhibitor of HER2 with growing interest in EC clinical management,^[^ ^26^, ^29^, ^30^ ^]^ after the identification of an actionable mutation (*ERBB2^R678Q^*) in the primary and the corresponding PDOX tumor. Interestingly, we have also found not only that *ERBB2^R678Q^* represents the main missense mutation in endometrioid EC but also that it can be targeted in PDOX tumors which, to the best of our knowledge, constitutes the first demonstration that this mutation can be pharmacologically tackled in vivo. However, it must be noted that other relevant endometrial cancer related genes (e.g., AT‐rich interaction domain 1A, *ARID1A*) were not included in our gene panel, which represents a limitation in our study. Thus, our data demonstrate that the retention of essential morphologic, genetic, and transcriptomic tumor traits within the PDOX models can be harnessed to tailor patient treatment in real time and maximize therapeutic benefit. Importantly, this system could potentially be also used to anticipate and predict tumor progression when included in coclinical trials.

Finally, our data also demonstrate that tumors engrafted in vivo may present features that can differ from the original tumor biopsy, as demonstrated by the detection of increased histological grade (e.g., PDOX3), or the progression into more aggressive histological types (such as *TP53*‐positive serous carcinoma in the case of PDOX13, or undifferentiated carcinoma as seen in PDOX7). While many factors may contribute to these differences,^[^ ^67^, ^68^, ^69^ ^]^ it seems clear that, in light of our findings, the orthotopic perpetuation of EC in mice may allow certain PDOX the opportunity to progress “in vivo” long after surgical resection. Strikingly, these observations maintain the parallelism with what can be seen in patients, since transformation of a preexisting endometrioid carcinoma into these aggressive histological types is a well‐recognized phenomenon and examples of mixed endometrioid–serous carcinomas^[^ ^70^ ^]^ or dedifferentiated carcinomas (endometrioid carcinomas with undifferentiated carcinoma)^[^ ^71^ ^]^ have been previously reported. Interestingly, recent data obtained by microdissection followed by DNA sequencing have demonstrated that, in the cases presenting a morphologic merge between these different tumor entities, the aggressive histological type results from the progression of the endometrioid component.^[^ ^70^, ^72^, ^73^, ^74^, ^75^ ^]^ Hence, our study shows not only that PDOX tumors may recapitulate the morphologic, immunohistochemical, and molecular features of the primary tumor, but also that it may provide a dynamic “in vivo” model to deepen unresolved questions in EC progression from a biological and mechanistic point of view.

Altogether, while these observations warrant additional studies in the PDX arena, our data indicate that EC‐patient‐derived PDOX models unambiguously represent suitable tools to enhance endometrial cancer research in the forthcoming years.

## 4 Experimental Section

### Establishment of PDOX in Mice and Treatment

Primary sample collection and tumor engraftment were approved by the IDIBELL Committee for Animal Experimentation (CEIC Bellvitge Hospital approval reference number PR047/18). Endometrioid endometrial tumors were obtained at the HUB/Bellvitge Hospital and the Catalan Institute of Oncology between 2010 and 2015. Ethical and legal protection guidelines of human subjects, including informed consent from the patient to implant the tumor in mice, were strictly followed and conducted according to the procedure 9111, assigned to AlbertoVillanueva, which was approved by the Generalitat de Catalunya. Selected nonnecrotic tissue fragments (2–3 mm^3^) from resected tumors were placed in supplemented Dulbecco's modified Eagle medium (DMEM) (10% fetal bovine serum, FBS, and penicillin/streptomycin) at room temperature. Six week old Hsd:Athymic Nude‐*Foxn1^nu^* mouse females were subjected to a lateral laparotomy (*n* = 2 to 4) under isofluorane‐induced anesthesia. Animal uteri were exposed and then, primary tumor resected pieces were anchored using prolene 7.0 sutures. Finally, the abdominal incision was closed with surgery staples. Tumor growth was monitored by careful inspection and abdominal palpation twice per week, for the generation of PDOX cohort and during in vivo treatments and harvested when the tumor reached a certain volume (≈300–500 mm^3^).^[^ ^56^ ^]^ Thereafter, small fragments of the tumor were transplanted into 2 to 4 new mice. For subsequent implantation, engrafted tumors at early mouse passages (#1–3) were cut in 6–8 mm^3^ fragments and stored in liquid nitrogen in FBS–10% dimethyl sulfoxide. Animals were housed in a sterile environment, cages and water were autoclaved, and bedding and food were *γ*‐ray sterilized. For tumor characterization, i.e., immunohistochemical analysis and molecular studies, early passage 1 (P1) tumors were selected. For trastuzumab treatment in vivo, engrafted tumors at early mouse passage 2 (P2) were used. Females orthotopically harboring the *ERBB2*‐mutated BX12 tumor were randomized and treated with 4 µg kg^−1^ of intraperitoneally (i.p.) injected trastuzumab for 4 weeks, twice per week. Total time elapsed for the in vivo experimentation was 85 days, 55 days between tumor engraftment until treatment, and 30 days of trastuzumab experimentation. A control group of females (*n* = 5) harboring *ERBB2*‐mutated BX12 was used as surrogate controls to indirectly estimate tumor growth of the experimental cohort. All animals were culled, and tumors were resected in parallel the same day. Trastuzumab was purchased from the HUB pharmacy service.

### Generation and Analysis of Patient‐Derived Organotypic Multicellular Tumor Spheroids

Tumor samples were collected in DMEM (Gibco, 11965‐092) and transported to the laboratory at 4 °C. Samples were transferred to a 10 cm tissue culture dish and minced into small pieces (2 mm or smaller) with a sterile scalpel. Fragments were collected in a tube with 20 mL of sterile phosphate‐buffered saline (PBS) and centrifuged at 200 *g* for 5 min. After that, minced samples were resuspended in DMEM supplemented with Liberase DH (Roche, 05401054) at 0.28 u mL^−1^ and digested for 1–2 h at 37 °C. Sample aliquots were observed every 20 min under optical microscopy to check for small cell aggregates. After digestion, samples were centrifuged at 200 *g* for 5 min, after which pellets were resuspended in 20 mL of PBS. Thereafter, samples were filtered through a 100 µm cell strainer and the filtrate was transferred to a 50 mL tube. Samples were finally centrifuged at 200 *g* for 5 min and the pellet containing spheroids was cultured in 6‐well ultralow attachment plates with Mammocult human medium kit (StemCell Technologies, 05620) supplemented with 4 µg mL^−1^ of heparin (StemCell Technologies, 7980) and 0.48 µg mL^−1^ of hydrocortisone solution (StemCell Technologies, 07925) at 37 °C and 5% CO_2_. After 10 days in culture, spheroids were collected, and media was removed after centrifugation at 100 *g* for 5 min. Fresh media containing 50 µg mL^−1^ of trastuzumab (Selleckchem, A2007) was added and the spheroids were kept in 6‐well ultralow attachment plate during 24 h at 37 °C and 5% CO_2_.

To analyze cell viability by flow cytometry, tumor spheroids were disaggregated after incubating with 200 µL of TrypLE Express (Gibco, 12604‐013) at 37 °C for 20–30 min. Samples were then brought to single cell suspensions by gentle pipetting, after which they were washed with PBS and resuspended in 500 µL of binding buffer (BD Biosciences, 556454) with 5 µL fluorescein‐isothiocyanate‐conjugated Annexin V (Immunostep S.L., ANXVDY‐200T) and 5 µL propidium iodide (PI) staining solution (BD Biosciences, 51‐66211E). Cells were incubated on ice for 30 min and analyzed using the FlowJo and Kaluza softwares. To estimate spheroid perimeters, 10× spheroid images were captured on an Olympus IX70 inverted microscope. Perimeters were then measured using the ImageJ software. To perform *Ki67* immunofluorescent stainings, spheroids were embedded in Histogel (Life Technologies S.A., HG‐4000‐012) and fixed in 4% paraformaldehyde (Life Technologies S.A., 28908) overnight at room temperature. After that, spheroids were embedded in paraffin and the immunofluorescence staining was performed as follows: first, paraffin sections were dewaxed and rehydrated to carry out an antigen retrieval with citrate buffer at pH = 6 on a DeCloaking chamber. For the immunodetection, samples were washed with tris‐buffered saline (TBS) 3 times for 5 min, blocked with TBS + 0.5% triton + 3% donkey serum for 1 h at room temperature, and finally incubated with 1:100 purified mouse anti‐*Ki67* (BD Pharmigen, 550609) for 48 h at 4 °C. A goat anti‐mouse immunoglobulin G (IgG, (H+L)) Alexa Fluor 568 (TermoFisher Scientific, A‐11004) was used as secondary antibody. Nuclei were stained with 4',6‐diamidino‐2‐phenylindole (DAPI) (Invitrogen, D21490). Differences in *Ki67* expression by immunofluorescence were conducted as previously reported:^[^ ^76^ ^]^ nuclear DAPI stainings were used to estimate nuclei dimensions using ImageJ on images captured on a Nikon Eclipse 80i vertical fluorescence microscope. Then, the ImageJ ROI Manager tool was used to measure the *Ki67* fluorescence intensity after which the intensity of *Ki67* staining per nuclei was normalized to its respective area. Alternatively, overlaid DAPI–*Ki67* images were used to calculate the percentage of *Ki67*‐positive nuclei. Statistical significances for all experimental procedures involving tumor spheroids were determined by Student's *t*‐test.

### Primary Tissue Analysis

Immunohistochemical studies: tissue blocks were sectioned at thickness of 3 µm, dried for 1 h at 65 °C before pretreatment procedure of deparaffinization, rehydration, and epitope retrieval in the Pretreatment Module, PT‐LINK (Agilent Technologies‐DAKO, Santa Clara, CA, USA) at 95 °C for 20 min in 50× Tris/EDTA (ethylenediaminetetraacetic acid) buffer, pH 9. Before staining the sections, endogenous peroxidase was blocked. The antibodies used were against *P53* (ready to use, clone DO‐7), *β‐catenin* (ready to use, clone *β‐catenin‐1*), *PTEN* (1:100 dilution, clone 6H2.1), *ESR* (ready to use, clone 1D5), *MSH6* (ready to use, clone EP49), *PMS2* (ready to use, clone EP51 from Agilent Technologies‐DAKO, Santa Clara, CA, USA), *IMP2* (1:100 dilution, clone EPR6741 from Abcam, Cambridge, MA), and *p16* (clone E6H4, Roche Diagnostics). After incubation, the reaction was visualized with the EnVision FLEX Detection Kit for *P53*, *ER*, *MSH6 PMS2*, *IMP2*, and *p16* or EnVision FLEX+ Mouse/Rabbit Linker Detection Kit (Agilent Technologies‐DAKO, Santa Clara, CA, USA) for *PTEN* and *β‐catenin*, using diaminobenzidine chromogen as a substrate. Slides were counterstained with hematoxylin. Immunoexpression was graded semiquantitatively by considering the percentage and the intensity of the staining. Histological scores (Hs) from two independent pathologists were obtained from each sample, encompassing values from 0 (no immunoreaction) to 300 (maximum immunoreactivity). The score was obtained by applying the following formula, Hs = 1*x* (% light staining) + 2*x* (% moderate staining) + 3*x* (% strong staining), as previously reported.^[^ ^77^ ^]^ Identification of POLE‐mutated tumors: POLE mutation status was determined after the identification of pathogenic mutations in the POLE exonuclease domain (exons 9–14) by Sanger sequencing and analysis with the SeqStudio genetic analyzer (Applied Biosystems).

### DNA Extraction and Amplicon Seq VHIO‐Card

DNA extraction was performed from 5 × 10 µm sliced sections of formalin‐fixed paraffin‐embedded (FFPE) material using the Maxwell FFPE Tissue LEV DNA Purification Kit. Tumor area content was evaluated by a pathologist. A minimum tumor content was set to 20%, in order to allow detection of 5% minimum allele frequency mutations. An initial multiplex‐polymerase chain reaction with a proofreading polymerase was performed on samples using a panel of over 800 primer pairs targeting frequent mutations in oncogenes plus several tumor suppressors, totaling 57 genes (Table S4, Supporting Information). Indexed libraries were pooled and loaded onto a HiSeq instrument and sequencing performed (2 × 100). Initial alignment was performed with Burrows‐Wheeler Aligner (BWA) after primer sequence clipping and variant calling performed with the GATK Unified Genotyper and VarScan2 followed by ANNOVAR annotation. Somatic single nucleotide polymorphisms (SNPs) were filtered out with dbSNP and 1000 genome datasets (minor allele frequency MAF > 0.05). All detected variants were manually checked.

### RNA Extraction

Total RNA was isolated from primary tumors (BX) and PDOX tumors from mice using the RNeasy Micro kit (Qiagen, 74004) according to the manufacturer instructions. Total RNA concentration was determined on a Thermo Scientific NanoDrop 2000c UV–vis Spectrophotometer (Thermo Scientific, Wilmington, DE). RNA integrity number was assessed using an Agilent 2100 Bioanalyzer to determine the quality of RNA.

### RNA‐Seq

RNA_sequencing data that support the findings of this study were deposited in the Gene Expression Omnibus (https://www.ncbi.nlm.nih.gov/geo/) under accession number GSE214657. Stranded 2 × 75 bp single end polyA capture messenger RNA (mRNA) sequencing was performed on a NextSeq500 Illumina sequencing platform at Newcastle University Genomics Core Facility (https://www.ncl.ac.uk/gcf/). Raw files were merged, and quality control assessed by FastQC analysis (https://www.bioinformatics.babraham.ac.uk/projects/fastqc/). Trimmomatic software^[^ ^78^ ^]^ was used to trim Illumina adaptors and bad quality reads. Then, reads were mapped over human reference transcriptome (hg19/GRCh38) with STAR.^[^ ^79^ ^]^ An annotation file in general transfer format (GTF) (downloaded from the UCSC Table Browser, using RefSeq genes table)^[^ ^80^ ^]^ including 23 687 genes and 41 970 transcript isoforms was used for the indexing step. Samples from xenografts were aligned against mouse transcriptome (mm10/GRCm38), previously indexed. R package XenofilteR^[^ ^81^ ^]^ was used for deconvolution of mouse and human reads on xenografts samples. XenofilteR is an accurate method developed to remove sequence reads of mouse origin from human sequences in DNA and RNA‐seq data with high sensitivity results. After removal of the mouse‐derived reads, aligned sequences were quantified with RNA‐Seq by Expectation‐Maximization software (RSEM),^[^ ^82^ ^]^ and gene expression matrix was extracted as transcripts per million and then transformed to log2 scale. Finally, not expressed genes were removed and an adjustment for reduction of the batch effect was performed with ComBat function from R package sva.

### DEG and Functional Analysis

To identify enrichment in specific cellular functions and pathways, a GSEA^[^ ^17^ ^]^ was performed comparing “Primary Tumor” samples versus “Xenografts–PDOXs” samples. GSEA performs a functional enrichment analysis under different conditions by nonparametric test of equality of distributions. Hallmarks collection from MsigDB was interrogated, including a total of 50 gene sets that summarized the most representative biological states and processes. A differential Expression Analysis was performed to identify DEGs between primary tumors and PDOXs. A linear model was fitted using the R package Limma,^[^ ^83^ ^]^ and a list of DEG with *p*‐value < 0.05 and logFC > abs(1.5) was extracted.

### Stromal Cell Infiltration Analysis

The R package MCP‐counter^[^ ^18^ ^]^ was used to quantify infiltration of nine cell types, including T and B lymphocytes, natural killer (NK) cells, monocytic lineage cells, myeloid dendritic cells, neutrophils, endothelial cells, and fibroblasts. Comparisons of cell types between groups were performed using nonparametric Mann–Whitney test, and differences were considered significant when *p* < 0.05. In order to infer the specie originating the tumor microenvironment cells (human or mouse) in the PDOX samples, four MCP‐counter gene sets (T cells, cytotoxic lymphocytes, endothelial cells, and fibroblasts) were selected. Then, gene set variation analysis from R package “GSVA” was performed using those gene profiles to obtain the enrichment scores.^[^ ^19^ ^]^ GSVA was performed both over the human expression matrix and the mouse expression matrix. The resulting scores were compared by the Mann–Whitney test.

## Conflict of Interest

The authors declare no conflict of interest.

## Author Contributions

X.M.‐G., A.V., and D.L.‐N. developed the project conception and designed the research. L.D.‐J., R.S.‐P., X.M.‐G., A.V., and D.L.‐N. prepared the paper. L.D.‐J., A.V., L.P.‐P., M.S., E.N.‐D., F.J.L., L.C.‐I., K.G., M.M.‐I., R.R.‐B., and F.V. conducted research (hands on conduct of the experiments and data collection). L.D.‐J., S.G.‐M., N.B., R.S.‐P., and A.V. analyzed data and performed statistical analysis. A.V., N.R., M.G., E.D., M.B., L.M, J.P., J.M.P., and X.M.‐G. coordinated human sample collection, clinical information, written consents, and intellectual content collection. All authors contributed to data interpretation and revised the paper.

## Supporting information

Supporting Information

## Data Availability

RNA_sequencing data that support the findings of this study have been deposited in the Gene Expression Omnibus (https://www.ncbi.nlm.nih.gov/geo/) under accession number GSE214657.
