# Supplementary material for: Generation and Integrated Analysis of Advanced Patient‐Derived Orthoxenograft Models (PDOX) for the Rational Assessment of Targeted Therapies in Endometrial Cancer
Source: Adv Sci (Weinh). 2022 Nov 14;10(1):2204211. doi: 10.1002/advs.202204211 (PMC9811454; doi:10.1002/advs.202204211)
Supplement: Supplementary file 1 — Supporting Information [file ADVS-10-2204211-s001.pdf]

SUPPORTING TABLES

Table S1: Patients characteristics

**Age**	(N=15)
Mean (±sd)	61.1 (±13.9)
Median	59
**BMI**	(N=15)
Mean (±sd)	30.3 (±5.1)
Median	31.5
**Tumor type**	(N=15)
Endometrioid	14 (93.3%)
Mixed (90% endometroid)	1 (6.7%)
**Grade**	(N=15)
1	6 (40%)
2	4 (26.7%)
3	5 (33.3%)
**FIGO**	(N=15)
IA	10 (66.7%)
IB	2 (13.3%)
II	2 (13.3%)
IIIC2	1 (6.7%)
**Treatment**	(N=15)
None	7 (46.7%)
BT	4 (26.7%)
BT+RT	3 (20.0%)
CT+RT	1 (6.7%)
**Recurrence**	(N=15)
No	13 (86.7%)
Yes	2 (13.3%)
**Death**	(N=15)
No	14 (93,3%)
Yes	1 (6,7%)
**Follow-up**	(N=15)

Mean months (CI)	59.27 (47.30-71.23)
**PDOX growth time**	(N=15)
Mean days (CI)	102.47 (84.57-120.36)

Table S2: Individual patient clinical parameters

Patient	Age	BMI	Treatment	Time-elapsed after primary tumor PDOX engraftment to first passage in mice
1	49	24	BT	128
2	50	34	BT	115
3	73	36	None	49
4	35	21	CT+RT	113
5	85	32.1	BT+RT	30
6	77	31	None	99
7	59	23.3	BT+RT	109
8	67	27.8	None	79
9	54	39	None	98
10	84	31.5	None	120
11	51	25	BT	90
12	61	33.8	BT+RT	152
13	58	30.9	None	96
14	59	34	None	113
15	54	31.7	BT	146

Table S3. Histologic characterization of primary tumors and PDOX

Case	TCGA	Histology		Grade		PTEN		TP53		CTNNB1 (nuclear)		ESR		MSH6		PMS2	
	BX	BX	PDOX	BX	PDOX	BX	PDOX	BX	PDOX	BX	PDOX	BX	PDOX	BX	PDOX	BX	PDOX
1	TP53	EEC	EEC	G2	G2	Loss	Loss	Abn	Abn	-	-	+	+	WT	WT	WT	WT
2	POLE	EEC	EEC	G3	G3	Loss	Loss	WT	WT	-	-	-	-	Loss	Loss	WT	WT
3	MSI	EEC	EEC	G1	G2	WT	WT	WT	WT	-	-	+	+	WT	WT	Loss	Loss
4	MSS	EEC	EEC	G3	G2	WT	WT	WT	WT	-	-	+	+	WT	WT	WT	WT
5	MSI	EEC	EEC	G3	G3	WT	WT	WT	WT	+	+	-	-	WT	WT	Loss	Loss
6	MSI	EEC	EEC	G2	G2	WT	WT	WT	WT	-	-	+	+	WT	WT	Loss	Loss
7	MSS	EEC	Undiff	G1	G3	WT	WT	WT	WT	-	-	+	-	WT	WT	WT	WT
8	MSI	EEC	EEC	G1	G1	Loss	Loss	WT	WT	-	-	+	+	WT	WT	Loss	Loss
9	MSS	EEC	EEC	G1	G1	Loss	Loss	WT	WT	+	+	+	+	WT	WT	WT	WT
10	MSI	EEC	EEC	G1	G1	WT	WT	WT	WT	-	-	+	+	WT	WT	Loss	Loss
11	MSI	Mixed	Mixed	G3	G3	Loss	Loss	WT	WT	-	-	+	+	WT	WT	Loss	Loss
12	MSI	EEC	EEC	G1	G1	Loss	Loss	WT	WT	-	-	+	+	WT	WT	Loss	Loss
13	MSS	EEC	Serous	G2	G2	Loss	Loss	WT	Abn	-	-	+	+	WT	WT	WT	WT
14	MSI	EEC	EEC	G2	G3	Loss	Loss	WT	WT	-	-	+	+	WT	WT	Loss	Loss
15	MSS	EEC	EEC	G3	G3	Loss	Loss	WT	WT	-	-	+	+	WT	WT	WT	WT

Table S4. Differentially expressed genes PDOX vs BX

Gene	logFC	AveExpr	t	P.Value	adj.P.Val	B statistic
C1QB	-7,814922925	1,868216456	-22,39080318	4,15916E-21	5,08307E-17	37,6826424
C1QA	-7,66019027	1,799580054	-21,66837235	1,11456E-20	5,08307E-17	36,76541243
TYROBP	-6,963280723	1,567376174	-21,64417598	1,15255E-20	5,08307E-17	36,73413673
C1QC	-7,412964263	1,598733799	-21,55820184	1,29864E-20	5,08307E-17	36,6227118
CLEC14A	-5,587954538	0,717256717	-21,46638945	1,47583E-20	5,08307E-17	36,50320678
SPI1	-5,444553187	0,707378125	-20,18458471	9,26759E-20	2,65995E-16	34,77743355
SLAMF8	-5,496187136	0,695962838	-19,3851532	3,06896E-19	6,68589E-16	33,64402193
FCER1G	-6,12959568	2,017399602	-19,37729833	3,10592E-19	6,68589E-16	33,63265678
ABI3	-4,66939749	0,295453738	-19,2351486	3,86008E-19	7,38604E-16	33,42619432
DCN	-7,912376879	2,860590238	-18,89782777	6,5013E-19	1,11959E-15	32,93021726
AIF1	-6,179118086	1,036547619	-18,6721115	9,25558E-19	1,449E-15	32,59350598
FCGR3A	-7,165966752	1,628361201	-18,41852404	1,38233E-18	1,98376E-15	32,21050324
MPEG1	-5,911680551	0,956197276	-18,29383127	1,68658E-18	2,23421E-15	32,02031775
APBB1IP	-4,580569852	0,182536593	-17,87256461	3,33081E-18	4,09713E-15	31,36854937
CD93	-6,414983117	1,236691756	-17,78718385	3,82956E-18	4,39659E-15	31,23468613
FCGR2A	-6,608132982	1,386464083	-17,74591951	4,09753E-18	4,41022E-15	31,1697741
CD2	-5,694804974	0,852421511	-17,58987979	5,29792E-18	5,36679E-15	30,92302868
SPARC	-9,141039395	4,207004753	-17,2772907	8,91442E-18	8,52863E-15	30,42254957
CYTH4	-5,048042807	0,600751332	-16,95232521	1,54376E-17	1,34053E-14	29,89333418
ELTD1	-5,61363943	0,814504256	-16,93232567	1,59726E-17	1,34053E-14	29,86046236
CSF1R	-6,023060225	1,249842964	-16,91873724	1,6347E-17	1,34053E-14	29,83810786
RGS1	-7,335967252	1,739307808	-16,68924523	2,42306E-17	1,89671E-14	29,45808693
SLA	-5,643033916	0,837008861	-16,5529819	3,06723E-17	2,29655E-14	29,23020781
CYYR1	-5,036357229	0,410430282	-16,28069082	4,93585E-17	3,54167E-14	28,76976765
FLI1	-4,980161875	0,56454293	-16,17588776	5,93768E-17	4,09011E-14	28,59072044
MS4A6A	-6,40371989	1,1169257	-16,00573571	8,03143E-17	5,27464E-14	28,29783731
GIMAP6	-4,7393148	0,407609434	-15,98933095	8,26985E-17	5,27464E-14	28,26945527
PLVAP	-6,255626616	1,920225786	-15,93044753	9,18724E-17	5,65048E-14	28,16736985
TMEM204	-4,644736136	0,426445028	-15,90771249	9,56887E-17	5,68226E-14	28,12786603
LCP2	-5,736803542	0,89674134	-15,86055586	1,04132E-16	5,97755E-14	28,04577042
MRVI1	-5,322397915	0,963304103	-15,64627091	1,53303E-16	8,51623E-14	27,67002284
CD3E	-4,892186917	0,737502524	-15,57963739	1,7304E-16	9,31223E-14	27,55227301
STAB1	-5,938827953	2,278400619	-15,46269602	2,14225E-16	1,09724E-13	27,34457073
ROBO4	-4,876452814	0,330478074	-15,45659621	2,16632E-16	1,09724E-13	27,33369976
COL15A1	-6,422871146	1,763815365	-15,32638371	2,75196E-16	1,35404E-13	27,10075767
CCR5	-4,57634218	0,180422757	-15,25280934	3,15254E-16	1,50805E-13	26,96839071
SASH3	-4,992300421	0,687620781	-15,05875286	4,52247E-16	2,1049E-13	26,61665211
GYPC	-4,94670184	0,466278541	-15,02978219	4,77424E-16	2,16361E-13	26,56381356
NDN	-4,942256415	0,476253756	-15,01282701	4,92822E-16	2,17612E-13	26,53284991
PDGFRB	-6,886855887	2,283122905	-14,99118579	5,13219E-16	2,20954E-13	26,49328592
GNLY	-5,179000123	0,504565816	-14,94023645	5,64736E-16	2,37203E-13	26,39995213
EVI2B	-5,300023245	0,661686826	-14,84456442	6,76307E-16	2,71133E-13	26,22396994
CD37	-4,487694842	0,157454344	-14,84401745	6,77006E-16	2,71133E-13	26,22296111
CXorf36	-4,683596467	0,234049901	-14,71652305	8,62048E-16	3,37394E-13	25,98696381
RAMP3	-5,455866508	0,814887089	-14,53805405	1,21222E-15	4,63901E-13	25,65375569
TREM2	-4,598552086	0,297242479	-14,51496872	1,26715E-15	4,74384E-13	25,61040931
CD247	-4,016658163	0,035744818	-14,41627501	1,53243E-15	5,6149E-13	25,42445796
LST1	-5,417246617	0,709925566	-14,32473763	1,82946E-15	6,56358E-13	25,25106065
DOK2	-3,744463153	-0,153983556	-14,2975389	1,92866E-15	6,77826E-13	25,19936539
LILRB4	-5,137129491	0,486844614	-14,21056925	2,28455E-15	7,86843E-13	25,03353112

ITGAX	-5,48024372	1,322660245	-14,19380007	2,36058E-15	7,97088E-13	25,00146144
PTPRC	-6,45268209	1,382046501	-14,17927669	2,42853E-15	8,04262E-13	24,97366199
RASAL3	-4,017620404	0,47345537	-14,16489507	2,49779E-15	8,11593E-13	24,94611133
HCK	-5,005590274	0,709010789	-14,09246755	2,87873E-15	9,18049E-13	24,80702065
CD84	-4,852444959	0,318474147	-14,01083357	3,38035E-15	1,05842E-12	24,64956224
LAIR1	-5,001141552	0,904185524	-13,97995127	3,59277E-15	1,10484E-12	24,58980462
IL16	-4,470612103	0,780129083	-13,8913048	4,28194E-15	1,29367E-12	24,41768799
TRAF3IP3	-3,982412551	-0,047550683	-13,7870555	5,26889E-15	1,56441E-12	24,21416135
VSIG4	-5,311579787	0,579069341	-13,74277986	5,75605E-15	1,68008E-12	24,12735488
TIGIT	-4,629314868	0,282714287	-13,70724077	6,18041E-15	1,77388E-12	24,05751831
DOCK2	-4,593025189	0,26203482	-13,67465597	6,59769E-15	1,8626E-12	23,99336242
FCGR1A	-4,914697064	0,349600199	-13,64229648	7,04074E-15	1,92877E-12	23,9295318
HOXD10	-4,608815104	0,385950336	-13,64121436	7,05608E-15	1,92877E-12	23,92739522
GIMAP1	-3,4874944	-0,309551203	-13,58779552	7,85719E-15	2,08384E-12	23,82175879
IGLL5	-8,478650234	2,321861191	-13,58727977	7,86537E-15	2,08384E-12	23,82073731
SPN	-3,729564621	-0,181237508	-13,57772615	8,01837E-15	2,09219E-12	23,80181031
LILRB2	-4,62087369	0,202688512	-13,54385484	8,58585E-15	2,20682E-12	23,73462323
ADORA3	-3,604059193	-0,14828729	-13,47470295	9,87595E-15	2,50108E-12	23,59704851
ARHGAP9	-4,482836457	0,6913504	-13,45693274	1,02385E-14	2,55533E-12	23,56160735
VWF	-5,849211507	2,882184865	-13,44949308	1,03944E-14	2,55716E-12	23,54675887
COL3A1	-8,647755135	4,456930782	-13,34925235	1,27479E-14	3,09199E-12	23,34607417
CD52	-5,943181402	1,217293922	-13,29549026	1,42289E-14	3,40328E-12	23,23796465
ARHGAP25	-4,648960956	0,926310079	-13,28269517	1,46067E-14	3,44578E-12	23,21218597
IKZF1	-4,410421333	0,522711158	-13,24487195	1,57848E-14	3,67338E-12	23,13587151
GIMAP7	-4,368013341	0,315045484	-13,19292101	1,75638E-14	4,03288E-12	23,03078147
NKG7	-4,972885178	0,447197582	-13,18270735	1,79371E-14	4,0644E-12	23,01008363
CNRIP1	-4,129165903	0,003003879	-13,17250146	1,83182E-14	4,09686E-12	22,9893894
HAVCR2	-5,003847329	0,878810438	-13,15379566	1,90385E-14	4,20336E-12	22,95142856
ANGPT2	-5,900526221	1,75635974	-13,14415673	1,94209E-14	4,23352E-12	22,93185177
FCGR2B	-5,169034647	0,47676899	-13,1040207	2,11E-14	4,54204E-12	22,85021813
PLEK	-5,76174741	1,129824181	-13,07080415	2,26019E-14	4,80527E-12	22,78251551
FLT1	-5,963811996	2,152036929	-13,05482921	2,33628E-14	4,90648E-12	22,74990898
GPR116	-5,000684407	1,111506685	-13,0156517	2,53422E-14	5,24557E-12	22,66981658
FPR3	-4,930021882	0,411104476	-13,01103197	2,55867E-14	5,24557E-12	22,66036033
ZEB2	-5,132420072	0,887809771	-13,00002066	2,61792E-14	5,30391E-12	22,63781087
C3AR1	-5,126822576	0,455662955	-12,94764529	2,91963E-14	5,8464E-12	22,53035823
FNDC1	-4,565656225	0,403478957	-12,90996727	3,15856E-14	6,25213E-12	22,45285777
GGT5	-4,33454745	0,750049659	-12,88937324	3,29754E-14	6,45305E-12	22,41042647
GPR4	-3,877871766	-0,145903675	-12,88074184	3,35763E-14	6,49683E-12	22,39262762
CD53	-6,037901657	1,842461847	-12,87123964	3,4251E-14	6,55373E-12	22,37302284
IL10RA	-4,689022902	1,17096223	-12,81250983	3,87407E-14	7,33136E-12	22,25161398
A2M	-7,722541104	3,972616434	-12,78768245	4,08162E-14	7,64017E-12	22,20016596
FGR	-4,851095032	0,630816439	-12,74251693	4,48892E-14	8,31222E-12	22,10638356
GZMA	-5,3202227	0,826330751	-12,73307596	4,57919E-14	8,38918E-12	22,0867493
CXCL9	-6,681974115	1,490395915	-12,71000549	4,80771E-14	8,71512E-12	22,03872491
FERMT3	-3,523580879	1,865521815	-12,64090522	5,56471E-14	9,98228E-12	21,89449999
IGSF6	-4,268014344	0,08070877	-12,51053769	7,34336E-14	1,30371E-11	21,62082755
NLRC3	-3,558224006	0,060574432	-12,49465931	7,59667E-14	1,33492E-11	21,58735409
APOC2	-4,86287055	0,528990322	-12,47998883	7,83868E-14	1,35553E-11	21,55639973
TESPA1	-2,76543485	-0,725030908	-12,47804211	7,87138E-14	1,35553E-11	21,55229022
CCL3	-5,202834279	0,562172132	-12,42435416	8,83056E-14	1,49774E-11	21,43877321
SFRP4	-6,431590506	1,545725076	-12,42221737	8,87113E-14	1,49774E-11	21,43424792
MYCT1	-3,830580752	-0,151060907	-12,35439334	1,02631E-13	1,71593E-11	21,2903197

CD27	-4,239336671	0,158549035	-12,3267838	1,08921E-13	1,80359E-11	21,23156822
TNFAIP8L2	-3,921301897	-0,147097384	-12,32088049	1,10317E-13	1,8093E-11	21,21899413
GMFG	-5,108151576	0,715626965	-12,28350711	1,19587E-13	1,94284E-11	21,13928911
AMICA1	-4,231808232	0,374251873	-12,25454325	1,27318E-13	2,04911E-11	21,07740027
NCKAP1L	-4,692905181	0,938864486	-12,22666414	1,35244E-13	2,15652E-11	21,01773132
FYB	-4,893387514	0,98972134	-12,15118114	1,59341E-13	2,51745E-11	20,85569354
ISLR	-6,035650956	2,545338612	-12,12443246	1,68901E-13	2,64423E-11	20,79810283
SAMD3	-3,086838379	-0,541515056	-12,07550287	1,87938E-13	2,91575E-11	20,69252525
COL6A3	-7,328289567	3,430832427	-12,05662211	1,9586E-13	3,01152E-11	20,65170546
LILRB3	-4,176036321	-0,019730172	-12,04292842	2,01819E-13	3,07568E-11	20,62207212
IL2RB	-4,528343545	1,013340625	-12,01969715	2,12356E-13	3,20788E-11	20,57174564
CXCR6	-4,307170052	0,076864474	-11,97446262	2,34522E-13	3,51192E-11	20,47355879
INMT	-3,258579073	-0,376877899	-11,96076323	2,41692E-13	3,56434E-11	20,443772
RGS5	-4,633747438	3,826987826	-11,95988004	2,42162E-13	3,56434E-11	20,44185085
LAPTM5	-6,42203236	3,724157134	-11,94918083	2,4793E-13	3,61831E-11	20,41856977
CD163	-5,940132615	0,973406238	-11,92826918	2,59612E-13	3,75696E-11	20,37302525
MMRN1	-4,255274808	0,134115259	-11,91768235	2,6574E-13	3,8136E-11	20,34994667
CARD11	-3,211026544	-0,296472951	-11,8801585	2,8868E-13	4,10856E-11	20,26803338
ITK	-4,120850039	-0,047323313	-11,87627665	2,91166E-13	4,10998E-11	20,25954927
NTM	-3,584182066	-0,114164657	-11,84646098	3,11008E-13	4,35436E-11	20,19432129
KLRK1	-4,231981835	0,079993649	-11,83342156	3,20116E-13	4,44574E-11	20,16575949
SIGLEC10	-4,421829043	0,103166188	-11,80807771	3,38608E-13	4,66493E-11	20,11018425
MYO1G	-3,41572415	0,117873146	-11,75818575	3,78277E-13	5,17008E-11	20,00054066
MS4A4A	-4,610229929	0,228394413	-11,75128161	3,8413E-13	5,19719E-11	19,98534308
IFFO1	-3,673719489	0,581508829	-11,74875314	3,86296E-13	5,19719E-11	19,97977583
GIMAP8	-4,039437563	0,372274734	-11,71031967	4,20815E-13	5,61771E-11	19,89505179
ITGAL	-4,63857996	1,188514212	-11,66630565	4,64252E-13	6,14073E-11	19,79779456
LILRB1	-3,737314085	0,063082122	-11,66354509	4,67125E-13	6,14073E-11	19,79168632
SLAMF7	-5,281770396	0,891770568	-11,63936778	4,93077E-13	6,43222E-11	19,73814823
CSF2RB	-4,627978573	0,238015192	-11,63603547	4,96768E-13	6,43222E-11	19,73076333
NID2	-4,833074577	0,722322599	-11,62766172	5,06169E-13	6,50503E-11	19,71219954
MS4A7	-4,883247561	0,392499873	-11,60171928	5,36466E-13	6,84332E-11	19,6546307
C1orf162	-3,664858686	1,20135071	-11,56886491	5,7752E-13	7,31284E-11	19,58159982
EMCN	-4,314929829	0,08149082	-11,52396229	6,38892E-13	8,03092E-11	19,48156316
CD86	-4,732927618	0,64365662	-11,51663087	6,49529E-13	8,10547E-11	19,46520518
HK3	-3,815060598	-0,009340655	-11,47203467	7,18263E-13	8,89871E-11	19,3655523
SLAMF6	-3,750392007	-0,191311585	-11,43082458	7,88398E-13	9,69786E-11	19,27323785
EVI2A	-5,339623214	0,670168608	-11,36794252	9,09203E-13	1,11026E-10	19,13195305
GIMAP4	-5,5616421	1,098532171	-11,36490654	9,15494E-13	1,11026E-10	19,1251188
OLFML2B	-4,70898361	1,571446235	-11,35360313	9,39311E-13	1,13118E-10	19,09966329
SH2D3C	-3,948405243	0,528721523	-11,2426213	1,20973E-12	1,44672E-10	18,8488485
SIGLEC9	-2,853873394	-0,639570892	-11,16023896	1,4611E-12	1,73528E-10	18,66162962
SRGN	-6,714019653	2,420742268	-11,12062908	1,60042E-12	1,88773E-10	18,57129786
THBS2	-5,913176652	1,469463165	-11,11297322	1,62888E-12	1,90822E-10	18,55381469
PRKCB	-3,859693536	-0,045931403	-11,09721498	1,6891E-12	1,9654E-10	18,51780449
SPARCL1	-6,854973046	2,903330912	-11,09399278	1,70169E-12	1,96676E-10	18,51043721
GPR65	-3,99912998	0,05086723	-11,04853621	1,88991E-12	2,16975E-10	18,40635956
IGJ	-7,400688599	1,963616011	-11,02298653	2,00494E-12	2,28656E-10	18,34774166
BIN2	-4,18294869	0,603575063	-10,98118737	2,20879E-12	2,50089E-10	18,2516578
CLEC2B	-5,303944767	1,173836468	-10,9786337	2,22191E-12	2,50089E-10	18,24578023
RUNX1T1	-3,514578639	-0,125848855	-10,92435519	2,5205E-12	2,81854E-10	18,12064805
CXCL12	-5,282811833	0,596253583	-10,91512452	2,57523E-12	2,86116E-10	18,09932924
IL21R	-3,520453397	-0,32461286	-10,89077892	2,72547E-12	3,0075E-10	18,04304752

GZMB	-5,139975282	0,736550237	-10,88820502	2,74187E-12	3,0075E-10	18,03709264
TNFRSF4	-2,788274219	-0,580214092	-10,88455056	2,76533E-12	3,01403E-10	18,02863631
MARCH1	-4,016623686	0,21074099	-10,75771396	3,72062E-12	4,02974E-10	17,73404359
CD3D	-4,48458665	0,461855493	-10,74339707	3,84785E-12	4,12937E-10	17,70065689
MSR1	-5,10281673	0,782813895	-10,74199152	3,86057E-12	4,12937E-10	17,69737769
C11orf96	-4,595153726	0,813965825	-10,7193392	4,07174E-12	4,31519E-10	17,64449301
CD48	-5,468936901	0,695223443	-10,71748625	4,08953E-12	4,31519E-10	17,64016405
LY86	-3,664807217	-0,275344725	-10,71541941	4,10946E-12	4,31519E-10	17,63533485
ALOX5AP	-5,200210363	0,869096822	-10,65807407	4,70408E-12	4,88073E-10	17,50111983
OLFML1	-4,021203003	0,26990168	-10,65801614	4,70473E-12	4,88073E-10	17,50098401
MYO1F	-4,039549504	1,628963933	-10,62426668	5,09519E-12	5,25415E-10	17,42178935
BTK	-3,779922924	0,067166933	-10,61994335	5,14755E-12	5,27654E-10	17,41163347
CCL4	-4,864107969	0,457891403	-10,57739567	5,69316E-12	5,8013E-10	17,31155222
EGFLAM	-3,764503463	0,173261078	-10,55601078	5,98939E-12	6,06725E-10	17,26115906
CLEC5A	-4,07970606	-0,016442489	-10,54497464	6,14838E-12	6,19189E-10	17,23512867
CLEC1A	-3,207508848	-0,210009607	-10,53870075	6,24069E-12	6,24831E-10	17,22032347
SULF1	-6,199735594	3,167045414	-10,51900809	6,53972E-12	6,50986E-10	17,17381837
SIRPG	-3,242091026	-0,343626254	-10,51250968	6,64159E-12	6,57326E-10	17,15846069
ZEB1	-4,24562681	1,706267938	-10,50740858	6,72269E-12	6,61551E-10	17,14640132
TEK	-4,155390403	0,166776535	-10,46260619	7,47994E-12	7,3142E-10	17,04033549
CLEC10A	-3,225750804	-0,494872931	-10,46049862	7,51764E-12	7,3142E-10	17,03533938
JAM3	-4,453439345	0,73412396	-10,43827022	7,92729E-12	7,66943E-10	16,98260942
COL5A1	-6,322382238	2,432825461	-10,428624	8,1121E-12	7,80438E-10	16,95970617
SIGLEC1	-4,20351705	0,322141298	-10,42624878	8,15828E-12	7,8052E-10	16,95406471
ADAMTS2	-5,296119701	1,247071415	-10,4081866	8,51834E-12	8,10466E-10	16,9111399
SLA2	-3,128620785	-0,262411933	-10,3602382	9,55512E-12	9,03471E-10	16,796978
HSPA7	-3,857742097	0,270929037	-10,35825036	9,60078E-12	9,03471E-10	16,79223842
RPL19P12	-2,843771315	1,121854586	-10,34268042	9,96629E-12	9,32769E-10	16,75509688
THBD	-4,216913071	0,634188802	-10,29347437	1,12177E-11	1,04422E-09	16,63750332
HAND2	-3,350555962	-0,432470352	-10,2770532	1,16702E-11	1,0763E-09	16,59818724
COX7A1	-3,781643124	0,067156788	-10,27644603	1,16873E-11	1,0763E-09	16,59673284
TAGAP	-3,951652228	0,133128558	-10,2722158	1,18071E-11	1,08154E-09	16,58659846
EXOC3L2	-2,619128911	-0,554551297	-10,25813274	1,22148E-11	1,10988E-09	16,55284225
KLRB1	-3,744555077	-0,235470794	-10,25709596	1,22454E-11	1,10988E-09	16,5503561
CYBB	-5,155146861	2,063661764	-10,2473436	1,25369E-11	1,12778E-09	16,52696333
PTPN7	-4,314827763	0,834992941	-10,24559848	1,25898E-11	1,12778E-09	16,522776
OR51E1	-2,994746267	-0,406229451	-10,24397383	1,26393E-11	1,12778E-09	16,51887735
CD8A	-4,607243577	1,59159468	-10,1862403	1,45322E-11	1,28999E-09	16,38010449
KCNMB1	-4,121464138	0,034963334	-10,17250302	1,50238E-11	1,32679E-09	16,34701834
WNT2	-4,757558123	1,290514149	-10,17014661	1,51098E-11	1,32758E-09	16,34134037
GPR183	-4,433634316	0,333089877	-10,16005903	1,54838E-11	1,35354E-09	16,31702511
PPP1R16B	-3,722124947	0,149227812	-10,11738926	1,71734E-11	1,49365E-09	16,21402132
DTX1	-3,255239176	-0,021988605	-10,10392706	1,77447E-11	1,53559E-09	16,18147292
PECAM1	-5,379243006	2,616952847	-10,10152774	1,78485E-11	1,53685E-09	16,17566936
MAGI2-AS3	-3,65070467	0,102182403	-10,09788197	1,80075E-11	1,54282E-09	16,16684936
ATP8B4	-3,077365307	0,02103526	-10,08849774	1,84234E-11	1,57064E-09	16,1441384
MRGPRF	-3,569299122	0,332786388	-10,03457681	2,10109E-11	1,77443E-09	16,01341288
NRXN2	-3,685556939	0,086081023	-10,03440147	2,10199E-11	1,77443E-09	16,01298717
WAS	-3,380386859	1,090503758	-10,02889612	2,13043E-11	1,78966E-09	15,9996178
IRF4	-3,271958314	-0,400018112	-10,01972783	2,17866E-11	1,8213E-09	15,97734418
ABCC9	-3,492789504	-0,251609511	-10,01311781	2,21413E-11	1,84201E-09	15,96127861
C3orf72	-3,353900168	-0,163183331	-9,993155594	2,32487E-11	1,92484E-09	15,91272491
MNDA	-4,216146871	0,000325103	-9,98933365	2,34671E-11	1,93362E-09	15,90342272

PCDH12	-3,546700074	0,034237479	-9,966025423	2,48452E-11	2,03742E-09	15,84665039
LSP1	-4,817914387	1,927639155	-9,960977267	2,51544E-11	2,04446E-09	15,83434482
CCR1	-3,805104746	0,87805979	-9,960748831	2,51684E-11	2,04446E-09	15,8337879
MYH11	-6,177888149	1,641745924	-9,916708512	2,804E-11	2,26703E-09	15,72628613
SIGLEC7	-2,699674688	-0,757910989	-9,912801502	2,83104E-11	2,27819E-09	15,71673651
THY1	-6,160316113	2,537175855	-9,893752638	2,96674E-11	2,37629E-09	15,67014722
TMEM119	-3,891483035	0,347954699	-9,877657098	3,08658E-11	2,46083E-09	15,63074286
HCST	-3,328686763	0,599052599	-9,848909069	3,31306E-11	2,62923E-09	15,56027609
NCF1	-3,594415094	0,232389395	-9,810247209	3,64469E-11	2,87652E-09	15,46533244
CD28	-3,186662041	-0,455792887	-9,808762493	3,65808E-11	2,87652E-09	15,46168233
GVINP1	-3,367514922	-0,243093667	-9,806563591	3,67801E-11	2,87904E-09	15,45627587
GAB3	-2,961984898	0,058032961	-9,781574199	3,91239E-11	3,04866E-09	15,3947883
FOXF1	-3,128555663	-0,439813021	-9,773471158	3,99162E-11	3,09639E-09	15,37483226
SAMSN1	-4,601610329	0,397139818	-9,742533816	4,30952E-11	3,32799E-09	15,29855857
CCL5	-5,123547936	2,4113772	-9,718199396	4,57766E-11	3,51928E-09	15,23847293
LOC100130231	-2,677148076	-0,769174295	-9,706340656	4,71445E-11	3,59279E-09	15,20916275
C16orf54	-2,813991081	-0,67472459	-9,706293304	4,71501E-11	3,59279E-09	15,20904568
FAM162B	-2,445244714	-0,862311888	-9,686165717	4,95683E-11	3,76042E-09	15,15925447
IL7R	-4,800806008	0,907512861	-9,63043819	5,69465E-11	4,30121E-09	15,02111109
TARP	-3,091182734	-0,562156966	-9,625319068	5,76782E-11	4,33745E-09	15,0084002
GIMAP1-GIMAP5	-3,646483373	-0,253478865	-9,620322353	5,84016E-11	4,37276E-09	14,99598984
ZNF683	-2,89682661	-0,659335028	-9,583811729	6,3977E-11	4,76947E-09	14,90520583
CETP	-2,324759223	-0,877725639	-9,546049156	7,0317E-11	5,21952E-09	14,81111935
OLR1	-4,062961302	0,00092936	-9,430836737	9,39212E-11	6,9417E-09	14,52287455
SELP	-2,980841027	-0,562877889	-9,417860534	9,7044E-11	7,14186E-09	14,49029775
HTR2B	-3,798028707	0,243759883	-9,401373243	1,01165E-10	7,41348E-09	14,44887362
SCIMP	-2,877641672	-0,550297194	-9,397774307	1,02088E-10	7,44942E-09	14,43982645
CD300LF	-3,314262727	-0,38037306	-9,373927764	1,08426E-10	7,8785E-09	14,3798359
GNG2	-4,426718919	0,887814068	-9,337494193	1,18894E-10	8,60284E-09	14,28803236
HNRNPKP3	-2,505125902	-0,855185382	-9,315968545	1,25558E-10	9,04701E-09	14,23370912
ZAP70	-3,409122524	0,437044831	-9,286751006	1,35218E-10	9,70245E-09	14,1598745
FCGR1B	-2,861566548	-0,608461733	-9,240985245	1,51898E-10	1,08541E-08	14,04399083
GIMAP5	-4,110729541	0,070802777	-9,213932095	1,62732E-10	1,15802E-08	13,97535718
COL6A2	-6,201470865	3,350693727	-9,205434643	1,66295E-10	1,1785E-08	13,95377894
FGF1	-3,367289765	0,509163549	-9,167396052	1,83246E-10	1,29331E-08	13,85706592
KIAA1755	-3,406654249	0,085929529	-9,159361921	1,87046E-10	1,31475E-08	13,83661436
KDR	-4,316053886	1,27277224	-9,130071054	2,01596E-10	1,41125E-08	13,76197882
HIC1	-3,44773281	0,177245276	-9,115733885	2,09133E-10	1,45809E-08	13,72540469
MRC1	-3,829150221	0,12575944	-9,109492143	2,12503E-10	1,47089E-08	13,70947341
BATF	-3,228284539	-0,033677813	-9,108141666	2,1324E-10	1,47089E-08	13,7060258
RASGRP2	-3,05647192	-0,312613463	-9,107607761	2,13532E-10	1,47089E-08	13,70466274
CD34	-4,330739302	2,250498487	-9,066206957	2,37454E-10	1,62916E-08	13,5988501
ZNF469	-3,151279368	-0,116339649	-9,059496792	2,41582E-10	1,6509E-08	13,58167863
BCL6B	-3,177294852	0,531193461	-9,036738259	2,56133E-10	1,74342E-08	13,52339431
SYNPO2	-4,878435453	1,799742126	-9,025466095	2,63668E-10	1,78765E-08	13,49450087
CD209	-2,790590072	-0,671212553	-9,020756019	2,66883E-10	1,80235E-08	13,48242272
NRIP2	-2,825638196	-0,694929235	-8,992057067	2,87356E-10	1,93303E-08	13,40876559
P2RY14	-3,270942497	-0,472277085	-8,971701557	3,02842E-10	2,02928E-08	13,35645585
APOE	-4,091510249	4,023385541	-8,94492084	3,24522E-10	2,16493E-08	13,28755071
CDH13	-4,160649274	0,12311391	-8,943332849	3,25856E-10	2,16493E-08	13,28346192
ECSCR	-3,625858231	0,563613636	-8,940699432	3,2808E-10	2,16493E-08	13,2766806
CTSW	-3,610712126	0,343141139	-8,940658958	3,28115E-10	2,16493E-08	13,27657637

MMP9	-5,259518313	1,461417678	-8,91334677	3,52125E-10	2,31448E-08	13,20619032
TRPC4	-2,679075883	-0,607653914	-8,909769252	3,554E-10	2,32186E-08	13,19696339
BHLHE22	-2,566951986	-0,82427234	-8,909178832	3,55944E-10	2,32186E-08	13,19544046
LOC100506 776	-2,523675983	-0,777407016	-8,902993692	3,61688E-10	2,35043E-08	13,17948363
ACVRL1	-4,322885638	1,541375697	-8,899341286	3,65125E-10	2,36384E-08	13,17005854
LILRA6	-2,814115308	-0,700690679	-8,896759651	3,67574E-10	2,37078E-08	13,16339552
ACTA2	-5,00622035	4,375001807	-8,8705146	3,93443E-10	2,52817E-08	13,09560884
CD14	-4,276048504	2,735375729	-8,85992487	4,044E-10	2,58891E-08	13,06823152
LYL1	-2,624598284	-0,303988285	-8,844472612	4,20949E-10	2,68487E-08	13,02825665
CNTN4	-3,589092585	-0,31320204	-8,82248152	4,45699E-10	2,83224E-08	12,97131154
GPR171	-2,75118492	-0,732155873	-8,81868919	4,50114E-10	2,84979E-08	12,961485
CCR2	-3,134052669	-0,540721998	-8,817102281	4,51975E-10	2,85109E-08	12,9573725
PXDN	-4,852009063	1,917676537	-8,81239742	4,57539E-10	2,86714E-08	12,94517782
CD96	-3,745646063	0,935681104	-8,812135799	4,5785E-10	2,86714E-08	12,94449963
LILRB5	-2,466525595	-0,874485535	-8,802896009	4,68988E-10	2,92625E-08	12,92054192
GZMH	-3,812705092	-0,201395787	-8,784138865	4,92456E-10	3,06158E-08	12,87187225
MEF2C	-4,213283527	1,284223277	-8,762928262	5,2044E-10	3,22392E-08	12,81678077
HAPLN1	-5,038951989	1,072896652	-8,756917238	5,28661E-10	3,26311E-08	12,80115726
ITGA11	-4,082590154	0,405640051	-8,720966709	5,80672E-10	3,57134E-08	12,70761772
VIPR2	-2,812189517	-0,677249614	-8,701617088	6,10795E-10	3,74324E-08	12,65720192
CMKLR1	-3,5591721	0,474709472	-8,688016096	6,32917E-10	3,86506E-08	12,62173497
WISP1	-4,155772722	0,960637577	-8,684446546	6,38857E-10	3,88755E-08	12,61242274
CD7	-3,043501676	0,535412623	-8,658173215	6,84364E-10	4,1498E-08	12,5438298
LY96	-3,778940482	0,251401571	-8,655068766	6,89955E-10	4,16902E-08	12,53571893
NUDT10	-3,025065117	-0,566489341	-8,645309657	7,07835E-10	4,26211E-08	12,51021352
DIO2	-4,68967287	2,52160994	-8,631751653	7,33462E-10	4,40103E-08	12,47475911
TFEC	-3,455141562	-0,100729894	-8,626185213	7,44255E-10	4,45028E-08	12,46019585
MIR143HG	-3,960169892	0,403470486	-8,623238956	7,50033E-10	4,46932E-08	12,45248604
IL12RB1	-2,708067104	-0,557231068	-8,618113794	7,60194E-10	4,51424E-08	12,43907175
SLC2A3	-5,133722251	2,020165581	-8,61032728	7,759E-10	4,59167E-08	12,41868526
SIGLEC14	-2,863272628	-0,676112019	-8,567791497	8,67722E-10	5,11748E-08	12,30718008
HTRA3	-4,119295635	1,371357533	-8,563653445	8,77226E-10	5,15588E-08	12,2963199
SP140	-3,214903438	0,028742235	-8,561629028	8,81915E-10	5,1658E-08	12,29100607
RNASEK-C17orf49	-4,035932143	1,152313262	-8,559833497	8,86094E-10	5,17269E-08	12,2862926
HOXD9	-4,089784698	0,886396937	-8,558296479	8,89688E-10	5,17612E-08	12,28225742
F13A1	-4,794870079	1,442639601	-8,548880743	9,12029E-10	5,28824E-08	12,25753134
TBX21	-2,134671271	-0,907264549	-8,529076505	9,60904E-10	5,55293E-08	12,20548727
MFNG	-3,093561781	1,39531282	-8,516076456	9,9443E-10	5,72745E-08	12,17129659
ACAN	-3,216760484	-0,478943058	-8,509058369	1,01302E-09	5,81508E-08	12,15282967
NCF1C	-2,975925944	-0,352682446	-8,485442079	1,07822E-09	6,1688E-08	12,09064079
RCSD1	-4,11587971	0,868422156	-8,457344306	1,16139E-09	6,62264E-08	12,01655744
ABCG2	-3,148421491	-0,13152089	-8,449060504	1,18714E-09	6,74711E-08	11,99469681
ALDH1A2	-3,875206908	1,033267689	-8,432340738	1,24089E-09	7,0294E-08	11,95054725
MMP11	-3,869175331	5,829341996	-8,420664449	1,27989E-09	7,19594E-08	11,9196941
APOC1	-4,799226042	3,117208197	-8,420652138	1,27993E-09	7,19594E-08	11,91966156
ICOS	-2,752707077	-0,731394794	-8,419800219	1,28283E-09	7,19594E-08	11,91740978
AOAH	-3,088350271	0,02035977	-8,416219462	1,29506E-09	7,241E-08	11,90794419
CCL18	-4,272847029	0,127855778	-8,402345852	1,34361E-09	7,48812E-08	11,87125437
MMP12	-5,678432666	1,048759813	-8,388174209	1,39511E-09	7,75007E-08	11,83375106
UBASH3A	-2,114119885	-0,757986633	-8,36105799	1,49933E-09	8,30224E-08	11,76192046
IL2RA	-3,590111577	-0,24338657	-8,313673706	1,70086E-09	9,38786E-08	11,63617604
CD33	-2,733560254	-0,684269868	-8,312477893	1,70629E-09	9,38786E-08	11,63299902

LPL	-3,092016041	0,27315618	-8,299181448	1,76785E-09	9,69558E-08	11,59766102
FCGR1C	-3,603575038	-0,197209358	-8,280885409	1,85627E-09	1,01265E-07	11,54899915
CXCR3	-2,646550398	-0,602516262	-8,280500497	1,85818E-09	1,01265E-07	11,54797495
GUCY1A2	-3,7268095	0,614722915	-8,277664864	1,87229E-09	1,01712E-07	11,54042912
HBB	-5,367833292	0,666026895	-8,260574516	1,95971E-09	1,06126E-07	11,49492906
FOLR2	-3,223497584	-0,219542055	-8,239307843	2,07432E-09	1,11981E-07	11,43825908
LUM	-7,106702301	3,976245145	-8,235476159	2,09569E-09	1,12781E-07	11,42804264
OSCAR	-2,463651393	-0,140944361	-8,163610585	2,5406E-09	1,36298E-07	11,23608774
TIE1	-4,219908044	1,127561578	-8,158664297	2,57455E-09	1,3769E-07	11,22285245
MAGEH1	-3,683646827	0,606676096	-8,156159693	2,59191E-09	1,3819E-07	11,21614946
P2RY13	-3,283328087	-0,466084289	-8,130900935	2,77385E-09	1,47434E-07	11,14850694
PDGFRA	-4,61800076	1,831925364	-8,12392196	2,82637E-09	1,49763E-07	11,12980346
ACAP1	-3,208564532	1,152463088	-8,108217681	2,9483E-09	1,55433E-07	11,08769444
LYZ	-5,442084959	3,032029891	-8,107821505	2,95144E-09	1,55433E-07	11,08663175
MS4A14	-2,548077974	-0,833709346	-8,100763851	3,00802E-09	1,5793E-07	11,06769731
CSF3R	-4,336017714	0,623328253	-8,074474095	3,22868E-09	1,69E-07	10,99711271
HBA2	-4,34468438	0,751508497	-8,052592615	3,42486E-09	1,78726E-07	10,9382992
SLAMF1	-2,73890043	-0,683848188	-8,041723438	3,52677E-09	1,83488E-07	10,90906305
GSPT2	-2,563033361	-0,713357771	-8,032106668	3,61951E-09	1,87506E-07	10,88318365
KLHL6	-3,416581129	0,662864791	-8,03146653	3,62577E-09	1,87506E-07	10,88146059
EDNRB	-3,362632861	-0,290356567	-8,012131178	3,82014E-09	1,96966E-07	10,82939225
CD4	-4,734147497	2,351463728	-7,999293821	3,95502E-09	2,03311E-07	10,79479734
GZMK	-3,988364977	-0,045062519	-7,996881188	3,9809E-09	2,04033E-07	10,7882934
FLJ44635	-2,249140098	1,050184406	-7,992122436	4,03246E-09	2,06062E-07	10,77546276
CLEC4A	-3,096196996	0,122103721	-7,979606156	4,17133E-09	2,12528E-07	10,74170305
EXOC3L1	-2,150814916	-0,716788933	-7,969914188	4,2822E-09	2,17457E-07	10,71554826
GPR34	-3,18447152	-0,453667151	-7,968955852	4,29333E-09	2,17457E-07	10,71296148
ECM2	-3,491474114	-0,028165582	-7,966137788	4,32621E-09	2,1848E-07	10,70535418
FOXL2	-2,996462812	-0,251096747	-7,959111698	4,40931E-09	2,22026E-07	10,68638327
SEMA6B	-3,155002909	0,297543588	-7,952653066	4,48713E-09	2,25286E-07	10,66893929
SH2D1A	-3,582877271	-0,316309697	-7,943032476	4,60565E-09	2,30564E-07	10,64294595
MSC	-3,24652059	-0,341275962	-7,92975706	4,77444E-09	2,38321E-07	10,60705961
ESM1	-5,324939956	1,255335163	-7,927013279	4,81011E-09	2,39407E-07	10,59963994
PARP15	-2,370345504	-0,922575581	-7,913530472	4,98932E-09	2,47611E-07	10,56316695
ARHGDIB	-4,462266162	4,194715893	-7,908379667	5,05957E-09	2,50376E-07	10,54922753
CD6	-2,928063618	0,940439165	-7,893456749	5,26881E-09	2,59983E-07	10,50882435
CPXM1	-4,899706366	2,145237536	-7,891079444	5,30295E-09	2,6092E-07	10,50238545
CD248	-4,707177395	1,428840856	-7,857237862	5,814E-09	2,85251E-07	10,41065318
RGS18	-2,723060764	-0,746217951	-7,854659262	5,85494E-09	2,85937E-07	10,40365797
PVRIG	-2,434610432	-0,700202922	-7,854266778	5,86119E-09	2,85937E-07	10,40259317
LMOD1	-4,158518669	0,86306976	-7,812169618	6,57327E-09	3,19769E-07	10,28827942
GPR84	-2,893748541	-0,47172956	-7,797670975	6,83837E-09	3,31728E-07	10,24886055
KCNJ8	-3,373601737	0,266366946	-7,780757299	7,16139E-09	3,46422E-07	10,20284463
TRPV2	-3,407831617	0,807195485	-7,770988442	7,35498E-09	3,5479E-07	10,17625196
GIPC3	-2,569052181	-0,402514863	-7,767426198	7,42689E-09	3,57258E-07	10,16655209
ITM2A	-3,940888265	0,436216837	-7,750864248	7,77071E-09	3,72756E-07	10,12143514
HLA-G	-3,378640367	0,461082658	-7,730494304	8,21586E-09	3,93015E-07	10,06590114
CADPS	-2,856121492	-0,519054718	-7,701251248	8,90061E-09	4,24591E-07	9,986093005
CD300A	-4,035140611	1,086294027	-7,681407383	9,39803E-09	4,47081E-07	9,931880616
ASPN	-4,518031303	0,404014194	-7,679622314	9,44414E-09	4,48037E-07	9,927001694
IL2RG	-4,549249198	2,194596451	-7,671084715	9,66786E-09	4,57391E-07	9,903661846
EMR2	-3,254592594	0,394052774	-7,667885601	9,75307E-09	4,60158E-07	9,894914057
RNASE6	-3,180673108	1,176880349	-7,625848384	1,09463E-08	5,15043E-07	9,779857861

CCND2	-4,469397033	2,055180746	-7,602013738	1,16876E-08	5,48426E-07	9,71453361
SRPX2	-3,656233318	1,003350321	-7,591815533	1,20202E-08	5,62499E-07	9,686563635
TCEAL7	-3,168406333	-0,400521932	-7,571867574	1,26988E-08	5,92644E-07	9,631820018
DAB2	-4,707023576	2,05186807	-7,553711932	1,33502E-08	6,21361E-07	9,581956581
DLL4	-3,712760948	1,384995552	-7,54038228	1,38499E-08	6,42883E-07	9,545324183
HEPH	-3,965445498	1,599866853	-7,538384892	1,39264E-08	6,44697E-07	9,53983329
CTSK	-2,715361979	3,830915885	-7,524504368	1,44701E-08	6,68068E-07	9,501663076
LPXN	-1,588263633	3,240789623	-7,513404868	1,49203E-08	6,8701E-07	9,471125173
CCM2L	-2,721167605	-0,490700946	-7,510542003	1,50387E-08	6,90616E-07	9,463246417
FCGR2C	-3,068267334	-0,550800578	-7,499910194	1,54868E-08	7,09304E-07	9,43397926
ADAMTS4	-3,747918622	0,816708628	-7,498229647	1,55589E-08	7,10714E-07	9,429351932
FOXP3	-2,167521678	0,248112995	-7,472155791	1,67216E-08	7,61805E-07	9,357519006
PDE1B	-2,683759398	-0,631735098	-7,458524409	1,73642E-08	7,88993E-07	9,319935432
RFTN1	-3,953432207	2,069307153	-7,447838533	1,78854E-08	8,10537E-07	9,290458966
HSPA12B	-2,918513828	-0,283171588	-7,436566879	1,84524E-08	8,34037E-07	9,259353319
PYHIN1	-2,452195939	-0,814945742	-7,422382823	1,91919E-08	8,65191E-07	9,220191106
ELK2AP	-5,289296691	0,536900013	-7,414697821	1,9605E-08	8,81509E-07	9,198963797
ADAMDEC1	-4,021473042	-0,097011812	-7,402467339	2,02812E-08	9,09539E-07	9,165168078
FCN1	-2,644081612	-0,785707527	-7,397430019	2,05666E-08	9,1994E-07	9,151244135
NCF1B	-2,625021383	-0,558447633	-7,394378585	2,07414E-08	9,25357E-07	9,142808183
SIT1	-2,729178839	-0,527241253	-7,388314692	2,10933E-08	9,37196E-07	9,126041091
TMEM130	-2,48253781	-0,761597335	-7,387933867	2,11156E-08	9,37196E-07	9,124987952
TLR7	-2,679397532	-0,630299585	-7,382173607	2,14559E-08	9,4985E-07	9,109056584
PLCL1	-2,203945161	-0,587883513	-7,376811251	2,17776E-08	9,61622E-07	9,094222559
SGIP1	-3,321469358	0,209317746	-7,372306732	2,20517E-08	9,71233E-07	9,081759243
SLCO2B1	-4,191591211	1,365003672	-7,335597648	2,44197E-08	1,07278E-06	8,980111246
GNGT2	-2,180874263	-0,74189871	-7,296449299	2,72303E-08	1,19321E-06	8,871553993
LAMC3	-3,86859022	0,947549658	-7,279907658	2,85144E-08	1,24631E-06	8,825636909
MGP	-5,116295236	4,155588571	-7,259691722	3,01675E-08	1,31523E-06	8,769482466
RNASE2	-2,090824736	-1,062335965	-7,25034185	3,09646E-08	1,34657E-06	8,743496938
CRTAM	-2,346412902	-0,842641733	-7,246913626	3,12622E-08	1,35609E-06	8,733966859
OSM	-3,47106035	-0,232111261	-7,241480983	3,17397E-08	1,37334E-06	8,718862283
JAK3	-3,70155945	1,571388863	-7,233312377	3,24717E-08	1,40149E-06	8,696145185
DOCK10	-3,29379013	1,179404775	-7,228143044	3,29437E-08	1,41831E-06	8,681765659
CD80	-2,190616632	-0,896258544	-7,217450213	3,39423E-08	1,45766E-06	8,65201289
EDNRA	-3,89443414	1,18785897	-7,213610466	3,43084E-08	1,46971E-06	8,641326003
CXorf21	-2,284936645	-0,965280011	-7,184120693	3,72563E-08	1,59204E-06	8,559200163
ARHGEF6	-3,38850526	1,460440632	-7,170947944	3,86548E-08	1,64771E-06	8,522487552
LDB2	-3,988942083	1,293130419	-7,163775358	3,94385E-08	1,67696E-06	8,50249026
LRRN4CL	-2,927296367	0,245288684	-7,138900896	4,22835E-08	1,79351E-06	8,433100673
SHE	-2,508576102	-0,216703853	-7,1357276	4,26611E-08	1,80508E-06	8,424244122
DENND1C	-2,890713452	0,829227461	-7,124518326	4,40224E-08	1,85811E-06	8,392951607
PLXDC1	-4,125022387	1,150262925	-7,118529455	4,47677E-08	1,88495E-06	8,3762277
COLEC12	-2,922476656	0,322631668	-7,091531974	4,82895E-08	2,02828E-06	8,300794321
HPGDS	-2,256901922	-0,922399428	-7,087979305	4,87734E-08	2,04362E-06	8,290862631
ITGB2	-4,587997363	2,817937336	-7,083574459	4,93802E-08	2,06402E-06	8,27854696
CCL4L1	-3,175549691	-0,496569517	-7,068916558	5,14549E-08	2,14035E-06	8,23755107
CCL4L2	-3,175549691	-0,496569517	-7,068916558	5,14549E-08	2,14035E-06	8,23755107
SDS	-3,230830681	0,366486359	-7,062563759	5,23814E-08	2,17364E-06	8,219776932
TNFSF13B	-3,09672287	0,703550497	-7,034413132	5,66943E-08	2,34695E-06	8,140970054
VCAM1	-4,301370909	1,215404544	-7,029633614	5,74615E-08	2,37301E-06	8,127582546
LILRA1	-1,59355545	-1,310970608	-7,018033315	5,93676E-08	2,44106E-06	8,095081057
FAM78A	-2,531679238	0,737279738	-7,01788216	5,93928E-08	2,44106E-06	8,09465747

PTPRCAP	-2,685828919	1,741067607	-7,011956307	6,03915E-08	2,4762E-06	8,078049624
STX11	-2,818784759	-0,054592373	-7,002264444	6,20617E-08	2,53863E-06	8,050880118
PAMR1	-4,295706105	1,928421221	-6,974348437	6,71386E-08	2,7398E-06	7,972574005
SOX18	-2,522377836	-0,103253397	-6,970902201	6,77938E-08	2,75999E-06	7,96290216
GPR85	-1,83117263	-0,866967592	-6,965154049	6,89012E-08	2,79846E-06	7,946767598
SCML4	-1,83652191	-1,16667329	-6,957351265	7,04337E-08	2,85397E-06	7,924861066
C1QTNF7	-3,019887734	-0,597804466	-6,954837073	7,09348E-08	2,86753E-06	7,917801231
CACNA2D4	-2,515801534	-0,394923862	-6,932405501	7,55686E-08	3,04268E-06	7,854788332
HLA-DQA1	-5,20962391	3,283021378	-6,931974273	7,56607E-08	3,04268E-06	7,853576521
DYSF	-2,87105293	1,27367826	-6,931333134	7,57977E-08	3,04268E-06	7,851774801
ATP1B2	-2,474802945	-0,605315535	-6,907730939	8,10207E-08	3,24478E-06	7,785422722
LAG3	-2,995530945	1,301935494	-6,905021365	8,16432E-08	3,26213E-06	7,777802228
MEG3	-3,874463701	0,149403159	-6,90182823	8,23829E-08	3,28407E-06	7,76882092
CORO1A	-2,672698981	3,278445534	-6,899341406	8,29638E-08	3,29958E-06	7,761825628
NLRC4	-2,311010782	-0,879227959	-6,896797385	8,35623E-08	3,31573E-06	7,75466888
LY9	-2,492242262	-0,861627202	-6,892736518	8,45266E-08	3,34628E-06	7,74324382
TNFRSF1B	-3,573012295	2,805171594	-6,879154019	8,78348E-08	3,46928E-06	7,705019608
RHOJ	-3,360675106	1,218760764	-6,878185039	8,80758E-08	3,47083E-06	7,702292066
SPOCK2	-2,37484459	3,507190798	-6,875355291	8,87833E-08	3,49072E-06	7,694326257
CXCL13	-5,092680602	1,264452548	-6,871293205	8,9809E-08	3,52301E-06	7,682890169
COL5A2	-3,397445018	4,34231704	-6,859051593	9,29732E-08	3,63884E-06	7,64841743
GAPT	-2,364895003	-0,925300831	-6,849015491	9,56512E-08	3,73517E-06	7,620145841
ITIH3	-2,134128351	-0,894999516	-6,8414734	9,7715E-08	3,80713E-06	7,59889417
FGD2	-2,691893646	0,421506862	-6,824211871	1,0261E-07	3,98881E-06	7,550237393
LOC100132891	-2,712774553	-0,605993354	-6,809615602	1,06942E-07	4,14784E-06	7,509073782
PDCD1LG2	-2,679312444	-0,053114252	-6,801201109	1,09523E-07	4,2384E-06	7,485335556
NCF4	-3,809628739	1,022661503	-6,765069369	1,21341E-07	4,68522E-06	7,383337029
ARHGEF15	-3,133071817	0,55695613	-6,735702949	1,31891E-07	5,07963E-06	7,30035799
APLNR	-3,036341834	0,420184116	-6,73502487	1,32145E-07	5,07963E-06	7,298441157
AIM2	-2,822100375	-0,464443205	-6,721636112	1,37269E-07	5,26482E-06	7,260585493
LYVE1	-2,433499865	-0,890998401	-6,710158867	1,4182E-07	5,4273E-06	7,228123107
TMEM255B	-2,224367721	-0,814968569	-6,708110907	1,42648E-07	5,44688E-06	7,222329535
STAT4	-2,609624079	0,641321363	-6,697395922	1,47061E-07	5,60296E-06	7,192011998
SNX20	-2,249729514	-0,761280578	-6,691244128	1,49656E-07	5,68926E-06	7,174601714
ARHGAP15	-3,643541236	0,711647291	-6,689591278	1,50362E-07	5,70348E-06	7,169923453
SVEP1	-3,123259164	0,987667125	-6,687910555	1,51082E-07	5,71822E-06	7,165166081
ADORA2A	-2,473718386	1,00858187	-6,669002729	1,59433E-07	6,02105E-06	7,111631399
GRP	-2,40597247	-0,834749885	-6,666110457	1,60751E-07	6,05754E-06	7,103439924
AQP1	-3,208726205	3,182864431	-6,648372023	1,69078E-07	6,35742E-06	7,053187217
IL3RA	-2,806534646	0,485443106	-6,633443501	1,76424E-07	6,61915E-06	7,010876399
S100B	-3,082943529	-0,293064796	-6,631110336	1,776E-07	6,64882E-06	7,004262161
ZNF831	-1,935369438	-1,003612505	-6,617792364	1,84471E-07	6,87966E-06	6,966499522
FCN3	-2,474978989	-0,736134143	-6,617612527	1,84566E-07	6,87966E-06	6,96598951
RAC2	-4,554720046	2,029561126	-6,613005009	1,87006E-07	6,95557E-06	6,952921916
NLRP3	-2,375399542	-0,649891074	-6,611060218	1,88046E-07	6,97917E-06	6,94740573
MEI1	-2,445898089	-0,301532535	-6,607657358	1,89879E-07	7,03206E-06	6,937753219
POU2F2	-2,408294367	0,732265349	-6,606452373	1,90533E-07	7,04112E-06	6,934334969
MMP19	-3,326146102	0,77558807	-6,601438633	1,93276E-07	7,12722E-06	6,920111052
CD79B	-2,978770735	-0,099826635	-6,583953864	2,03161E-07	7,47572E-06	6,870492554
MZB1	-3,848851147	0,649673485	-6,57021039	2,11288E-07	7,75819E-06	6,831475544
HOPX	-4,141794936	2,074408741	-6,553871068	2,21379E-07	8,11141E-06	6,785071398
DARC	-3,494489171	-0,02587318	-6,549041511	2,24453E-07	8,20661E-06	6,771351674
SLC2A5	-2,817178033	0,821127366	-6,525730919	2,39912E-07	8,75322E-06	6,705108244

ENPP2	-4,310523604	2,472640132	-6,500454756	2,57891E-07	9,38932E-06	6,633236459
TNFSF8	-3,163036507	-0,069234491	-6,493089069	2,63383E-07	9,56901E-06	6,612284193
ABCA8	-2,922015726	-0,580035849	-6,485886517	2,68866E-07	9,74767E-06	6,591792426
LAT2	-2,9378371	1,514628391	-6,476986057	2,75802E-07	9,97813E-06	6,566465175
PPAPDC3	-2,040819424	-0,895612296	-6,47052957	2,80946E-07	1,01429E-05	6,54808921
CD1C	-2,446682867	-0,884406899	-6,461460064	2,88336E-07	1,0388E-05	6,522271598
ITGAM	-2,972278543	0,680237646	-6,43843441	3,07992E-07	1,10729E-05	6,45670163
ISM1	-3,319691067	1,465173846	-6,413450575	3,30853E-07	1,187E-05	6,385516697
LRRC25	-3,040790725	0,380034169	-6,412229582	3,32013E-07	1,18869E-05	6,382036778
EMILIN1	-3,958390382	1,721401078	-6,402163915	3,41735E-07	1,22096E-05	6,353345336
KIR2DL4	-1,689259621	-1,263118522	-6,383334268	3,607E-07	1,28605E-05	6,29965589
ADAM12	-3,111798222	0,028878531	-6,369562539	3,75239E-07	1,33512E-05	6,260374438
C1orf54	-2,746451767	1,751647316	-6,354236977	3,92116E-07	1,39229E-05	6,216647488
STMN2	-2,017842009	-0,916191178	-6,351122836	3,95637E-07	1,40191E-05	6,207760494
CCDC102B	-2,249178052	-0,380374932	-6,33929937	4,09302E-07	1,44735E-05	6,174014006
GJD3	-1,801385481	0,516547766	-6,318618949	4,34356E-07	1,5328E-05	6,114968412
PIK3R6	-2,264031795	-0,653077445	-6,317371927	4,35916E-07	1,53515E-05	6,111407196
PIEZO2	-1,899633427	-1,157931619	-6,28144913	4,83343E-07	1,6987E-05	6,008782006
LOC158376	-1,677715076	-1,192103797	-6,270835407	4,98327E-07	1,7478E-05	5,978446748
RAPGEF4	-2,094769218	0,585001605	-6,26336812	5,09149E-07	1,78213E-05	5,957100704
TAL1	-1,915243952	-0,911327607	-6,253417378	5,2394E-07	1,83018E-05	5,928650788
HBA1	-2,927777308	-0,314980958	-6,24740201	5,3309E-07	1,85837E-05	5,911449846
GREM1	-3,950239615	0,377429282	-6,244231055	5,37978E-07	1,87161E-05	5,902381734
TRAF1	-2,17208335	2,50249429	-6,24353139	5,39063E-07	1,87161E-05	5,900380801
HLA-DRB6	-3,935445948	2,161329638	-6,237004742	5,49288E-07	1,90328E-05	5,881714374
DCHS1	-2,824516287	2,216150082	-6,23499341	5,52478E-07	1,91049E-05	5,875961452
GRAP2	-1,88513326	-1,043548882	-6,226554239	5,66068E-07	1,95356E-05	5,851820985
CD244	-2,111309102	-0,769357098	-6,225298442	5,68119E-07	1,95672E-05	5,848228432
PCED1B-AS1	-2,407904955	-0,877767654	-6,211889444	5,9049E-07	2,02971E-05	5,809863291
RASGRP4	-1,822740334	-1,196378166	-6,20821852	5,96768E-07	2,0472E-05	5,799358644
PRF1	-3,631407653	0,548155574	-6,205734437	6,01055E-07	2,05621E-05	5,792249856
ZBTB46	-2,737732848	0,623991448	-6,205314182	6,01783E-07	2,05621E-05	5,791047167
RARRES2	-4,003034412	3,580234252	-6,199527457	6,11901E-07	2,08664E-05	5,774485779
CLMP	-2,648285375	-0,412166141	-6,197886193	6,14802E-07	2,09239E-05	5,76978824
PDCD1	-2,801971617	-0,098299756	-6,181482719	6,44566E-07	2,18936E-05	5,722831827
KLRC1	-2,021468232	-1,097014217	-6,18021636	6,46923E-07	2,19118E-05	5,719206218
PDPN	-3,965547207	1,160451418	-6,179829147	6,47646E-07	2,19118E-05	5,718097605
COL1A1	-3,353715926	7,197402238	-6,176478424	6,53932E-07	2,20811E-05	5,708503984
IL18RAP	-2,371333438	-0,473968105	-6,162194211	6,81429E-07	2,29645E-05	5,667600139
CD226	-2,086983059	-0,638278071	-6,147047568	7,11859E-07	2,39432E-05	5,6242162
HAAO	-2,547266214	-0,235425435	-6,144702077	7,16692E-07	2,40553E-05	5,617497152
CLIP3	-2,023866615	2,577344558	-6,144077631	7,17984E-07	2,40553E-05	5,615708281
SLC7A7	-3,207009696	1,666888571	-6,139778198	7,26946E-07	2,43082E-05	5,603391068
NOVA2	-1,830921679	-1,023274644	-6,135594922	7,35773E-07	2,45557E-05	5,591405819
FAP	-4,334173701	1,521727682	-6,134474422	7,38156E-07	2,45876E-05	5,58819541
PIK3CG	-2,683890222	-0,734775441	-6,121799516	7,65657E-07	2,54234E-05	5,551875876
PLA2G2D	-2,940083836	-0,568715041	-6,121553082	7,66201E-07	2,54234E-05	5,551169657
MEDAG	-2,681909551	-0,54689215	-6,119365671	7,71054E-07	2,55352E-05	5,544900957
AKT3	-3,427525656	1,119710513	-6,103885685	8,06293E-07	2,6651E-05	5,500532302
LRRC32	-3,269819233	2,272472648	-6,097879618	8,20398E-07	2,70653E-05	5,483314963
P2RY10	-2,405097789	-0,869389101	-6,091608866	8,3539E-07	2,75072E-05	5,465337226
FASLG	-2,367735267	-0,706220719	-6,075716406	8,74633E-07	2,87444E-05	5,419767472
SELPLG	-2,398562343	2,159266159	-6,072244543	8,8345E-07	2,89788E-05	5,409810931

AMPH	-1,988285603	-0,728702053	-6,066166215	8,99102E-07	2,94362E-05	5,39237844
ITGBL1	-2,553368559	-0,247493517	-6,058042004	9,20459E-07	3,00782E-05	5,369076095
ANKRD30BL	-1,926764074	-0,856314464	-6,048845145	9,45253E-07	3,07913E-05	5,342693962
GABRD	-1,668552672	-1,097554721	-6,048624085	9,45858E-07	3,07913E-05	5,342059786
CYTIP	-3,586622581	1,478799422	-6,044628467	9,56845E-07	3,10902E-05	5,330596884
WFDC1	-2,968958667	0,998911576	-6,040127711	9,69375E-07	3,14381E-05	5,317684067
ARHGAP20	-1,779188633	-1,082458021	-6,033569979	9,87929E-07	3,19796E-05	5,298868333
CRMP1	-2,762905492	0,564572928	-6,023928088	1,01586E-06	3,2822E-05	5,271200446
CALCRL	-4,081104063	1,461649975	-6,02196995	1,02163E-06	3,29465E-05	5,265581049
PCDH17	-3,444049087	0,38763563	-6,002470482	1,0809E-06	3,47929E-05	5,209614498
AEBP1	-4,122469484	4,050535969	-5,995547801	1,10277E-06	3,54306E-05	5,189742037
FPR1	-3,640160672	0,093551551	-5,994064732	1,10751E-06	3,55166E-05	5,185484476
SLC11A1	-2,878485363	1,338948247	-5,988009516	1,12709E-06	3,60772E-05	5,168100499
TNFRSF9	-2,887079724	0,017221549	-5,96305083	1,21151E-06	3,87078E-05	5,096433256
MMP3	-4,070519181	0,312386149	-5,960926	1,21899E-06	3,88745E-05	5,090330992
TMEM176B	-4,15800786	2,771660661	-5,956798198	1,23364E-06	3,92691E-05	5,078476011
IL18BP	-1,688161362	2,406073662	-5,949640826	1,25947E-06	4,00174E-05	5,057918865
DKK3	-3,662409779	2,600465429	-5,946645631	1,27045E-06	4,02916E-05	5,049315695
CCL8	-2,68604861	-0,35602559	-5,940920126	1,29168E-06	4,08899E-05	5,032869413
FLT4	-2,946286467	0,681182198	-5,911298284	1,40741E-06	4,44715E-05	4,947766083
CD79A	-3,225194744	0,396700368	-5,90560107	1,43083E-06	4,51288E-05	4,931395105
ARL11	-2,46347059	-0,534512592	-5,897643622	1,46421E-06	4,6044E-05	4,908527815
ADAM33	-3,197814998	0,270118051	-5,897411392	1,46519E-06	4,6044E-05	4,90786043
PIK3R5	-3,51126834	0,554973723	-5,890812105	1,49348E-06	4,68475E-05	4,888894725
TPSAB1	-3,61280465	-0,224112755	-5,8833268	1,52624E-06	4,76365E-05	4,867381278
MAP4K1	-2,560038187	1,023393605	-5,883169787	1,52693E-06	4,76365E-05	4,866929994
PDE10A	-2,514631386	-0,120347718	-5,870421466	1,58442E-06	4,93404E-05	4,83028675
CPA3	-3,574051436	-0,320722615	-5,867828097	1,59637E-06	4,9623E-05	4,822831971
PSTPIP1	-2,71235284	0,490313482	-5,864954723	1,60973E-06	4,9948E-05	4,814572109
AGAP2	-2,384271698	0,246945109	-5,857629355	1,64428E-06	5,09284E-05	4,793513527
CCRL2	-2,622952241	0,030015873	-5,847515701	1,69322E-06	5,22212E-05	4,76443721
VENTX	-1,577821895	-1,277057096	-5,846980008	1,69585E-06	5,22212E-05	4,76289705
LOC100499405	-2,040309413	-0,285145509	-5,846512763	1,69815E-06	5,22212E-05	4,761553676
BCL2A1	-3,995192578	1,146279686	-5,841541377	1,72281E-06	5,28852E-05	4,747260162
FCRL5	-2,829796169	-0,692850249	-5,837193225	1,74468E-06	5,3461E-05	4,734758083
ITGA8	-1,770417397	-1,222539635	-5,836325002	1,74908E-06	5,35006E-05	4,732261662
TSPYL5	-3,374549487	0,108518147	-5,828810663	1,78762E-06	5,45826E-05	4,710654822
KLRD1	-2,594992484	-0,406306065	-5,826629617	1,79896E-06	5,48318E-05	4,704383179
JAKMIP2	-1,617989334	-1,024265314	-5,823137869	1,81728E-06	5,52922E-05	4,69434237
FMO1	-1,819090112	-1,061576465	-5,821698007	1,82489E-06	5,54257E-05	4,690201855
PRKG1	-2,817350133	0,128288491	-5,8187155	1,84074E-06	5,58089E-05	4,681625115
LTA	-1,511124548	-1,002052873	-5,812269917	1,87549E-06	5,67624E-05	4,663089042
PIK3CD	-2,522540525	1,997118906	-5,810985168	1,88249E-06	5,68745E-05	4,659394286
GAL3ST4	-2,569495697	1,342974313	-5,807289824	1,90279E-06	5,73869E-05	4,648766813
GFRA1	-2,572258248	-0,534065916	-5,80490311	1,91601E-06	5,76847E-05	4,641902696
GRIK3	-2,249006943	-0,836378499	-5,800593258	1,94012E-06	5,82118E-05	4,629507408
CD300C	-1,743947133	-1,093863309	-5,800565137	1,94028E-06	5,82118E-05	4,629426529
CLIC2	-2,505573255	1,911413585	-5,794668131	1,97377E-06	5,91136E-05	4,612465947
LILRA5	-2,680135092	-0,767680787	-5,792773951	1,98465E-06	5,93167E-05	4,60701789
TMEM156	-1,991041921	-0,931374964	-5,792290048	1,98744E-06	5,93167E-05	4,605626073
CCL21	-3,860779097	-0,054118549	-5,791484866	1,99209E-06	5,93526E-05	4,603310173
SIGLEC11	-1,572544616	-1,321476025	-5,787300031	2,01643E-06	5,99741E-05	4,591273376

FAM65C	-2,286139615	0,987125251	-5,780993291	2,05368E-06	6,09767E-05	4,573132766
LOC100505702	-2,211013657	0,197702925	-5,775880698	2,08439E-06	6,17818E-05	4,558426461
NAALADL1	-2,628310876	-0,266024002	-5,763454534	2,16095E-06	6,39411E-05	4,522680912
GBP5	-3,86040661	2,561356567	-5,760107737	2,18205E-06	6,44547E-05	4,513052959
C5AR1	-2,961732413	1,486517645	-5,753140717	2,22664E-06	6,56591E-05	4,493009904
APOBR	-2,346192346	1,183879203	-5,746913946	2,26726E-06	6,67428E-05	4,4750958
SIGLEC5	-1,995886606	-1,10980503	-5,725779836	2,41078E-06	7,08464E-05	4,41428989
SLC9A9	-2,435876156	0,41854432	-5,711612528	2,51205E-06	7,35716E-05	4,373525195
TPSB2	-3,542590611	-0,281716118	-5,694804007	2,63777E-06	7,71222E-05	4,325157703
DNAJC5B	-2,038876992	-0,69878135	-5,675635841	2,78885E-06	8,12628E-05	4,269996701
TMEM176A	-3,691201563	2,369269365	-5,675457381	2,7903E-06	8,12628E-05	4,269483125
F2RL2	-2,624376999	-0,07007708	-5,675057383	2,79354E-06	8,12628E-05	4,268331998
LILRA2	-1,991694765	-1,11190095	-5,665568373	2,87165E-06	8,3394E-05	4,241023853
CTLA4	-3,227927821	0,045301011	-5,653868953	2,97097E-06	8,61332E-05	4,207353505
CCL19	-2,561583897	-0,826956384	-5,644296783	3,0548E-06	8,84146E-05	4,179804605
PREX1	-3,669806827	2,674248102	-5,640588764	3,0879E-06	8,92228E-05	4,169132707
GPR124	-3,4917187	2,15807095	-5,636650683	3,12346E-06	9,00989E-05	4,157798598
NOTCH4	-2,638358381	2,269666	-5,634304685	3,14483E-06	9,05432E-05	4,151046592
AVPR1A	-2,630670732	-0,019904193	-5,633517812	3,15203E-06	9,05432E-05	4,148781891
COL5A3	-3,120608401	0,854453444	-5,633234096	3,15463E-06	9,05432E-05	4,147965327
S1PR1	-3,212688756	1,458863032	-5,632456063	3,16178E-06	9,05973E-05	4,145726064
IGDCC4	-2,102540053	-0,258208235	-5,631445985	3,17107E-06	9,07127E-05	4,142818948
CNN1	-3,840449095	1,233625038	-5,609803202	3,37702E-06	9,6444E-05	4,080527666
TAGLN	-3,359023492	5,452321276	-5,608457996	3,39026E-06	9,66617E-05	4,076655907
RHOH	-3,074380787	0,659019214	-5,604799302	3,42652E-06	9,7534E-05	4,066125469
LOC606724	-1,644507507	-1,05226574	-5,598177654	3,49314E-06	9,92662E-05	4,047067003
TLR8	-2,445695961	-0,884900353	-5,592428275	3,55203E-06	0,000100726	4,03051906
POM121L9P	-1,660075056	-1,129096952	-5,592022704	3,55622E-06	0,000100726	4,029351739
C1QTNF1	-2,550509033	0,730930141	-5,587334913	3,60503E-06	0,000101941	4,015859248
WNT5A	-2,595925586	4,130298408	-5,582771599	3,6532E-06	0,000103134	4,002725025
PLA2G4C	-2,051307074	0,245627046	-5,581901223	3,66246E-06	0,000103226	4,00021989
GSTM5	-2,189274469	-0,945926588	-5,570491706	3,78603E-06	0,000106535	3,967380818
HAMP	-1,999785519	-1,025129034	-5,565290513	3,84374E-06	0,000107982	3,952410697
C1S	-3,766164993	4,660788931	-5,562684555	3,87298E-06	0,000108626	3,944910224
CD300E	-2,540326097	-0,837585284	-5,539255771	4,14614E-06	0,0001158	3,877478254
XCL2	-2,484857484	-0,865319591	-5,538508099	4,15517E-06	0,0001158	3,87532636
FAM167B	-2,091749063	0,454475562	-5,538469122	4,15564E-06	0,0001158	3,875214179
DES	-4,57597525	1,007142584	-5,536062496	4,18483E-06	0,000116425	3,868287624
IFNG	-2,567666078	-0,823915294	-5,52901447	4,27153E-06	0,000118542	3,848002732
FAM110D	-1,537311365	-0,643327637	-5,528758386	4,27471E-06	0,000118542	3,847265703
RAMP2	-2,804809915	2,008032189	-5,524237631	4,33131E-06	0,000119919	3,834254678
ACTG2	-3,56508927	1,879282772	-5,505632987	4,57222E-06	0,000126386	3,78071064
CPED1	-3,556714666	0,706879112	-5,502400807	4,61543E-06	0,000127375	3,771408698
GJA5	-3,000282098	1,170412585	-5,477366663	4,96419E-06	0,000136781	3,69936556
SEPT1	-1,866480132	1,270468933	-5,460586074	5,21263E-06	0,000142983	3,651077994
CD19	-2,08725865	-0,609691274	-5,460485085	5,21416E-06	0,000142983	3,6507874
ERG	-3,142747625	1,994331074	-5,454541391	5,30514E-06	0,000145016	3,633684714
TREM1	-3,080484927	-0,014297601	-5,444431572	5,46355E-06	0,000149109	3,604595245
BGN	-4,690474904	4,34762303	-5,443189724	5,48333E-06	0,000149314	3,601022109
LOC441081	-2,118245843	0,499893111	-5,442871927	5,48841E-06	0,000149314	3,600107724
CPZ	-3,331675303	1,171558607	-5,439393555	5,54425E-06	0,000150596	3,590099629
DDR2	-2,29954938	0,007793396	-5,433734459	5,63633E-06	0,000152855	3,573817449

TOX2	-2,260025927	-0,301195093	-5,425241516	5,77739E-06	0,000156189	3,549382699
FGL2	-3,768504721	1,872992547	-5,423993066	5,79842E-06	0,000156512	3,54579092
CES1	-3,014220881	0,344725052	-5,417465038	5,90964E-06	0,000159264	3,527010254
HTRA4	-1,579503762	-1,000441919	-5,414021852	5,96917E-06	0,000160617	3,517104735
HIGD1B	-1,976559254	-1,119468706	-5,4129105	5,98851E-06	0,000160886	3,513907587
ADAP2	-2,699069519	1,325247446	-5,394079788	6,32591E-06	0,000169494	3,459738519
AXL	-3,093463443	1,90947508	-5,384464761	6,50545E-06	0,000173691	3,432082137
MS4A1	-2,910216294	-0,652640186	-5,375333894	6,68067E-06	0,000178093	3,405820079
C19orf38	-1,940031404	-0,212150634	-5,371114797	6,76323E-06	0,000180015	3,393685757
ACP5	-3,426586708	2,824480395	-5,362953588	6,92582E-06	0,000184058	3,370214818
CCR6	-1,701439916	-0,234144075	-5,349651745	7,19925E-06	0,00019103	3,331963041
ENG	-2,945197517	4,373758404	-5,347896015	7,23614E-06	0,000191713	3,326914438
SH2D2A	-2,05805285	1,111384461	-5,343836294	7,32216E-06	0,000193694	3,315240989
GRIP2	-1,58409711	-0,582440005	-5,342563621	7,34933E-06	0,000194115	3,311581585
S1PR4	-1,930574906	-0,441686429	-5,317010822	7,91683E-06	0,000208077	3,238116523
TLR10	-2,298322608	-0,646954741	-5,316601347	7,92628E-06	0,000208077	3,23693941
HLA-DPA1	-4,666562626	4,767777621	-5,313457015	7,99916E-06	0,00020967	3,22790058
GPR82	-1,643846314	-1,224003092	-5,298598006	8,35273E-06	0,000218605	3,185189971
TNFRSF8	-1,74094977	-0,570178456	-5,295998419	8,41618E-06	0,000219598	3,177718389
LAYN	-2,634294048	0,982845892	-5,294589813	8,45076E-06	0,000220167	3,173669937
RFTN2	-2,040043383	-0,558405021	-5,291600887	8,5246E-06	0,000221551	3,165079711
GEM	-2,645971347	2,299048841	-5,291400074	8,52958E-06	0,000221551	3,164502581
NR5A2	-3,34795845	0,708204567	-5,283244288	8,7345E-06	0,000226191	3,141064119
PCDHB15	-1,522633024	-0,453367441	-5,277532399	8,88093E-06	0,000229637	3,124650249
ABCB1	-2,857318228	0,21315037	-5,276038425	8,91964E-06	0,000230292	3,120357287
XYLT1	-2,284606603	-0,049437442	-5,255335768	9,47364E-06	0,000243865	3,060875204
INHBA	-2,877841664	-0,086119679	-5,254127236	9,50703E-06	0,000244359	3,057403333
BTLA	-1,827987022	-0,960130679	-5,240048948	9,90467E-06	0,0002542	3,01696295
WIPF1	-3,190296464	3,269686619	-5,239468925	9,92141E-06	0,000254251	3,015296962
CD163L1	-1,950453571	-0,216228197	-5,2343093	1,00715E-05	0,000257714	3,000477613
MMP25	-2,400939937	0,085731166	-5,223494071	1,03936E-05	0,00026556	2,969417517
BCL11B	-2,151931224	-0,328458753	-5,222870562	1,04125E-05	0,000265649	2,967627004
CYP7B1	-1,713148874	-0,970859066	-5,215424362	1,06406E-05	0,000271067	2,946245085
PTGIS	-2,468347684	-0,152158936	-5,210391731	1,07975E-05	0,000274359	2,93179498
LINC00341	-1,602186485	-0,721635217	-5,210260902	1,08017E-05	0,000274359	2,931419347
RGS4	-3,592648981	0,873113611	-5,209749977	1,08177E-05	0,000274362	2,929952394
P2RX7	-1,919052643	-0,402281904	-5,201431571	1,10828E-05	0,000280672	2,906070284
LRCH2	-2,07793173	-0,794438095	-5,200894728	1,11001E-05	0,000280698	2,904529102
PLCB2	-2,866549094	2,014438729	-5,196592572	1,124E-05	0,000283402	2,892178803
GPR97	-2,485820675	0,079974973	-5,177620471	1,18779E-05	0,000298613	2,837724216
ZNF300P1	-1,978466743	-1,071296913	-5,16686427	1,22555E-05	0,000307657	2,806858055
HLA-DQA2	-3,28389515	0,352527623	-5,164538919	1,23387E-05	0,000309294	2,800185854
COL8A1	-3,207312044	0,334308322	-5,154145891	1,27175E-05	0,000318071	2,770367769
TMEM140	-2,182215512	1,911338332	-5,153587019	1,27382E-05	0,000318071	2,768764478
FHL1	-3,407331436	1,715490397	-5,153423501	1,27443E-05	0,000318071	2,76829538
APOBEC3H	-1,671617485	-0,402381025	-5,146693481	1,29963E-05	0,000323891	2,74898947
P2RY8	-2,282921381	-0,438459665	-5,139740035	1,32618E-05	0,000330032	2,729044838
ANO4	-1,978964149	-0,79842307	-5,124901514	1,38468E-05	0,000343999	2,686491046
LOC654433	-2,469267943	-0,032367395	-5,124498646	1,3863E-05	0,000343999	2,685335852
LRRN3	-2,26559769	-0,670304627	-5,122584755	1,39404E-05	0,000345016	2,679848025
CD1D	-2,435382237	0,182444604	-5,122494557	1,39441E-05	0,000345016	2,679589399
TM6SF1	-2,101080494	-0,273175235	-5,108197079	1,45362E-05	0,00035915	2,638599104
TIMP3	-3,878852977	3,262987597	-5,106388797	1,46128E-05	0,000360527	2,633415567

KLRC2	-1,962107018	-1,058191498	-5,102395595	1,47835E-05	0,000364216	2,621969433
DACT3	-2,221273791	0,388044496	-5,098259826	1,49624E-05	0,000368097	2,610115508
PLXNC1	-2,982329479	2,188812184	-5,094653617	1,51202E-05	0,0003711	2,599780134
ENPEP	-2,832813143	0,297637437	-5,094484615	1,51276E-05	0,0003711	2,599295793
SLC6A1	-1,567691139	-1,186416089	-5,088377242	1,53987E-05	0,000376963	2,581793715
PPP2R2B	-1,895551732	-0,552166865	-5,088115818	1,54104E-05	0,000376963	2,581044588
TMEM200A	-3,177394271	1,348025285	-5,07984723	1,57854E-05	0,000385589	2,55735234
TSHZ3	-3,113286215	0,83481144	-5,07597651	1,5964E-05	0,000389401	2,546262717
LOC643733	-2,246934713	-0,586422984	-5,072546833	1,6124E-05	0,000392747	2,536437367
TRAT1	-1,973349006	-1,053226687	-5,066490659	1,64105E-05	0,000398596	2,519089211
HEYL	-2,405469533	1,655125764	-5,064778771	1,64924E-05	0,000400021	2,514185809
STARD8	-2,414719947	0,717576331	-5,063699954	1,65442E-05	0,000400713	2,511095813
HLA-F	-3,852794289	2,823926958	-5,060025292	1,67219E-05	0,000403957	2,50057117
POU2AF1	-2,782751448	0,208904168	-5,059960262	1,6725E-05	0,000403957	2,500384924
RGCC	-2,722411611	2,933005568	-5,050209612	1,72059E-05	0,00041499	2,472461742
TBC1D10C	-2,177611753	1,686275252	-5,046611246	1,73868E-05	0,000418767	2,462158396
RNASE1	-3,329002028	4,437918743	-5,043768923	1,7531E-05	0,000421651	2,454020393
ZNF208	-1,772632253	-0,929427926	-5,042891098	1,75758E-05	0,000422139	2,451507144
PLXDC2	-3,019042867	2,453569552	-5,040350449	1,77061E-05	0,000424675	2,444233411
IGSF21	-1,508810549	-1,353343058	-5,036813663	1,78891E-05	0,000428467	2,434108434
GPR176	-1,94217143	0,798794974	-5,023737718	1,8582E-05	0,000444445	2,396681633
SCARF1	-1,996082784	1,479734405	-5,022690128	1,86387E-05	0,000445182	2,393683603
ADAMTSL1	-3,00772007	0,696202515	-5,002136594	1,97858E-05	0,000471926	2,334876595
MFAP4	-4,373374975	1,711552449	-5,000402944	1,98857E-05	0,000473653	2,329917559
PRKCDBP	-3,508239314	1,025643176	-4,985086735	2,07905E-05	0,000494521	2,286114628
HGF	-1,871353848	-0,81751642	-4,983304018	2,08984E-05	0,000496402	2,281017223
HVCN1	-1,993480171	0,730091699	-4,979915135	2,11052E-05	0,000500623	2,271327814
TTN	-1,524314481	-0,954408403	-4,978486033	2,1193E-05	0,000502014	2,267241992
RASSF2	-2,969671731	2,253316546	-4,97633748	2,13256E-05	0,000504462	2,261099505
ARHGAP6	-1,915917244	-0,472771727	-4,973628513	2,14941E-05	0,00050775	2,253355299
EDIL3	-2,614017004	-0,588580707	-4,971589371	2,16218E-05	0,000510066	2,247526269
HSD11B1	-2,260494037	-0,631345672	-4,965189216	2,20274E-05	0,000518925	2,229232812
CD72	-2,062022261	1,09456352	-4,963063949	2,21638E-05	0,000521425	2,223158817
NPTX2	-2,703961205	0,157219114	-4,961883753	2,22399E-05	0,000522501	2,21978596
MYLK	-3,135433569	4,273389348	-4,95539346	2,2663E-05	0,000531019	2,201239224
DPEP2	-1,802492699	-0,66737133	-4,955376704	2,26641E-05	0,000531019	2,201191348
THEMIS	-2,454100452	-0,532815842	-4,953812149	2,27673E-05	0,000532712	2,196720897
GPR18	-1,599328524	-1,193049317	-4,953233374	2,28056E-05	0,000532884	2,195067189
HOXD11	-2,029178134	-0,429129014	-4,943997953	2,34254E-05	0,000546625	2,168682441
PLN	-3,010357004	-0,55640057	-4,93991446	2,37048E-05	0,000552396	2,157018213
DOCK8	-3,062628155	2,239276535	-4,927808203	2,45527E-05	0,000571381	2,12244457
CDH11	-3,549792493	2,905347885	-4,92243833	2,49384E-05	0,000579573	2,107112462
TSPAN18	-2,618227837	0,287311717	-4,921291518	2,50215E-05	0,000580722	2,103838349
ESAM	-3,295713004	2,16143534	-4,912802953	2,56456E-05	0,000593606	2,079606824
SIGLEC8	-1,718936135	-1,248280265	-4,911984523	2,57066E-05	0,000594219	2,077270813
IKZF3	-2,862423747	1,923978111	-4,911449673	2,57465E-05	0,000594344	2,075744242
SEPT4	-2,231424085	0,974758563	-4,909857037	2,58658E-05	0,000596298	2,071198655
CHRD	-2,973284857	1,32269889	-4,905579888	2,61888E-05	0,000602938	2,058992085
GBP4	-3,04467972	3,175140749	-4,901154177	2,65273E-05	0,000609916	2,046363004
FCGR3B	-2,836213797	-0,483763405	-4,887668424	2,75858E-05	0,000633406	2,0078898
KNDC1	-1,617956701	-1,030664091	-4,855332186	3,0298E-05	0,000693831	1,915697483
C16orf45	-2,480492464	1,10532049	-4,854428849	3,03774E-05	0,000694727	1,913123249
COL1A2	-4,600019983	6,448511458	-4,853892912	3,04247E-05	0,000694885	1,911596025

N4BP2L1	-2,069545798	2,216844295	-4,844593032	3,12561E-05	0,000711986	1,885098597
MARCO	-2,375492516	-0,59403079	-4,841217796	3,15634E-05	0,000718036	1,875483602
MMRN2	-3,128836008	1,793561758	-4,838018979	3,18574E-05	0,000723769	1,866372065
HLX	-2,040194141	0,838470468	-4,834932172	3,21437E-05	0,000729311	1,857580409
HLA-DPB1	-4,270523457	4,384657937	-4,823389593	3,32371E-05	0,000751151	1,824712815
DLGAP1-AS1	-1,599763929	2,533535371	-4,820168235	3,35488E-05	0,000757201	1,815542042
SYDE1	-1,989358577	1,70820707	-4,819492223	3,36146E-05	0,000757693	1,813617643
CARD16	-3,106884569	2,043098297	-4,817779735	3,37818E-05	0,000759596	1,808742893
LAT	-2,080420827	2,065541305	-4,817724114	3,37873E-05	0,000759596	1,808584568
FHL5	-1,790823321	-1,036528181	-4,816746024	3,38832E-05	0,000760759	1,80580047
TMEM215	-2,116481437	-0,690284171	-4,813807063	3,41729E-05	0,000766265	1,797435329
IGF2	-4,400266538	2,778027305	-4,809148092	3,46373E-05	0,000775668	1,784176109
FCER1A	-2,232868196	-0,991314235	-4,797646347	3,58107E-05	0,000799865	1,751451011
COL6A1	-3,400222067	5,391756927	-4,795368747	3,60477E-05	0,000804116	1,744972138
RRN3P2	-1,958460888	-0,317286728	-4,772685341	3,84948E-05	0,000853998	1,680472948
LOC728392	-2,478350829	0,866430563	-4,769506112	3,88507E-05	0,00085996	1,671436809
TDO2	-2,239250947	-0,154492557	-4,76482416	3,93808E-05	0,000870574	1,658131319
P2RY6	-1,799813855	0,365832092	-4,762212129	3,96797E-05	0,000876056	1,65070918
APOL3	-2,238252188	2,403583325	-4,744660443	4,1747E-05	0,000919342	1,600852825
C2	-2,622048953	2,538799281	-4,740776319	4,22188E-05	0,000927359	1,589823869
COL22A1	-2,104571654	-0,485367316	-4,740330775	4,22732E-05	0,000927373	1,588558843
ZDBF2	-2,056034241	-0,30475689	-4,738611925	4,2484E-05	0,00093081	1,583678727
SIGLEC12	-1,541445962	-1,337025352	-4,73782434	4,25809E-05	0,000931747	1,581442733
TNXB	-2,75898431	0,897370625	-4,733771547	4,3083E-05	0,000940345	1,569937623
TM4SF18	-2,416553482	0,840983893	-4,732449395	4,32481E-05	0,000942753	1,56618464
SFMBT2	-2,719184985	0,842325109	-4,727211621	4,39082E-05	0,000955933	1,551318729
FAM26F	-2,793698286	1,438901185	-4,719371853	4,4915E-05	0,000976618	1,529072989
ARHGAP30	-2,247487947	2,17028466	-4,707165837	4,65283E-05	0,001010421	1,494450312
FOXS1	-2,734791509	0,047942583	-4,688308694	4,91344E-05	0,001064332	1,440992055
FBXL7	-3,47080377	1,07268821	-4,686473032	4,93957E-05	0,001067585	1,43579013
ZNF781	-1,629653328	-0,291715946	-4,686013705	4,94613E-05	0,001067585	1,434488541
DKK2	-1,963903038	-0,831245477	-4,685948717	4,94706E-05	0,001067585	1,434304387
CCL13	-2,386015326	-0,91474067	-4,684601176	4,96636E-05	0,001070408	1,430486011
APOBEC3G	-3,041721188	2,438818346	-4,683705219	4,97923E-05	0,001071841	1,427947348
PEG3	-2,293032611	-0,574133076	-4,682685612	4,99392E-05	0,001073661	1,42505843
APOD	-3,173336319	0,670704913	-4,681936778	5,00473E-05	0,001074645	1,42293678
JAM2	-3,295521412	0,546613824	-4,67821223	5,05887E-05	0,001084917	1,412385031
CMAHP	-2,906664878	1,137625595	-4,671850768	5,15269E-05	0,001103663	1,394366295
RLTPR	-1,756075655	0,313633801	-4,670905512	5,16678E-05	0,001105305	1,391689248
LHFP	-1,786047657	3,117985518	-4,667953949	5,21101E-05	0,001113384	1,383330799
LOC100505495	-2,854791519	1,041559119	-4,654102529	5,42365E-05	0,001156082	1,344118145
ARHGAP4	-2,129832597	2,899135346	-4,654062486	5,42428E-05	0,001156082	1,344004817
ST8SIA4	-3,575221366	1,642552118	-4,65335435	5,43538E-05	0,001157016	1,342000697
DPP6	-2,24282543	-0,847098042	-4,646378963	5,54593E-05	0,001176188	1,322262403
MME	-3,959648801	1,606353676	-4,645157681	5,56551E-05	0,00117889	1,318807099
SNORA25	-1,560036721	-0,772334739	-4,635641863	5,72047E-05	0,001210224	1,291890309
CD1A	-2,179867358	-1,017814654	-4,634381542	5,74131E-05	0,001213143	1,288326092
RERG	-2,759772749	0,141875322	-4,606077254	6,2297E-05	0,001313118	1,208329409
FILIP1L	-2,828528548	2,755947262	-4,603568839	6,27492E-05	0,001319143	1,20124439
GPSM3	-1,936510041	2,972894612	-4,603218021	6,28127E-05	0,001319143	1,200253563
ST6GALNAC3	-2,463890471	0,37624769	-4,595941323	6,41442E-05	0,001345466	1,179705087
RECK	-2,270153504	0,705007587	-4,593595775	6,45794E-05	0,001351965	1,173082908

ROBO3	-1,975271972	-0,237556896	-4,59295481	6,46988E-05	0,001352157	1,17127339
FAM65B	-2,365665092	0,783374137	-4,591956888	6,48851E-05	0,001354408	1,168456239
FGF7	-2,541793095	0,180728248	-4,589320376	6,538E-05	0,001361557	1,161013901
GALNT15	-1,838093981	-0,54956885	-4,58873521	6,54904E-05	0,001362089	1,15936221
ZMIZ1-AS1	-1,528018448	-0,181599458	-4,587003263	6,58181E-05	0,001367253	1,154473855
CFP	-1,755281293	0,042607385	-4,585103145	6,61794E-05	0,001373104	1,149111265
PCOLCE	-3,392903281	3,471168162	-4,570540586	6,9015E-05	0,001428494	1,108026912
NAPSB	-3,37817065	1,701367214	-4,569941746	6,91341E-05	0,001429242	1,106338007
CXCR2P1	-1,814689503	-1,177589494	-4,562263472	7,06799E-05	0,001459447	1,084686964
PRAM1	-1,745875459	-0,479657931	-4,56111207	7,09147E-05	0,001462541	1,081440899
PDLIM3	-3,130633651	0,620306361	-4,558889606	7,13699E-05	0,001468413	1,075175736
CLEC12A	-1,875771153	-1,169862756	-4,55403372	7,23747E-05	0,001485537	1,061489086
GNG7	-2,033552914	-0,506848437	-4,543284055	7,46492E-05	0,001530398	1,03120106
TNFRSF17	-1,936428667	-1,118178744	-4,538393905	7,57071E-05	0,001550241	1,017427574
TMEM26	-2,753848422	0,415861286	-4,53688861	7,60358E-05	0,001555121	1,013188413
ZNF626	-2,742377279	-0,203124519	-4,512276967	8,16135E-05	0,001661307	0,943919924
HECW2	-2,032979693	0,324232609	-4,499044372	8,47777E-05	0,00172368	0,906710514
KCTD12	-2,90390339	3,216548451	-4,497924274	8,5051E-05	0,001727198	0,903561936
PTPRN2	-2,239946227	0,27857257	-4,497496252	8,51557E-05	0,001727287	0,902358816
C3orf70	-1,854623171	-0,690512267	-4,493878349	8,60457E-05	0,001743285	0,892190317
VGLL3	-2,579914722	-0,004156585	-4,491750124	8,65735E-05	0,001751917	0,886209546
CACNA1H	-2,893605192	0,500545794	-4,490948525	8,67731E-05	0,001753896	0,883957041
LINC00667	-2,952018045	0,648960817	-4,487681269	8,75914E-05	0,001768361	0,874776911
EPB41L3	-3,286960918	1,318522524	-4,478906299	8,98273E-05	0,001811377	0,850128811
HLA-DQB2	-2,513379794	0,285882868	-4,477551629	9,01775E-05	0,001814221	0,84632461
CCL14	-2,276374313	-0,769962038	-4,477545477	9,01791E-05	0,001814221	0,846307336
NPL	-2,292208117	1,84107045	-4,459192301	9,50582E-05	0,001903485	0,794793005
DPYSL4	-2,129120774	-0,466641235	-4,457627824	9,5486E-05	0,001909729	0,790403968
IL33	-3,513346158	2,328377155	-4,457241851	9,55918E-05	0,001909729	0,789321201
TMEM200B	-2,466015987	0,685210771	-4,447592389	9,82755E-05	0,001961069	0,762258509
LOC100216479	-2,287762929	0,25403204	-4,43788256	0,000101051	0,002011795	0,735039997
GFI1	-1,881921909	0,199785374	-4,436080283	0,000101575	0,002019885	0,729989371
MYL9	-2,890715769	4,96214008	-4,433527919	0,000102321	0,002027698	0,722837536
CLEC3B	-2,124120567	-0,222296614	-4,428372952	0,000103845	0,002055526	0,708395995
NTNG2	-1,5145988	-0,905969393	-4,427517826	0,000104099	0,002058206	0,706000753
VASH1	-2,26267808	2,803159978	-4,420589	0,000106188	0,002094683	0,686596796
LPPR4	-2,381833773	0,442777196	-4,419363648	0,000106561	0,002099648	0,683165972
RASA3	-2,590667337	1,746500586	-4,413577116	0,000108343	0,002132316	0,666967459
COL10A1	-2,348230451	-0,815002805	-4,412991945	0,000108525	0,002133456	0,665329642
CST7	-2,962388592	0,677600589	-4,410657761	0,000109253	0,002142913	0,658797064
ST8SIA1	-1,710432526	-0,45166598	-4,410652328	0,000109255	0,002142913	0,65878186
SOGA3	-1,818308358	-0,859192211	-4,401114129	0,000112281	0,002197266	0,632096219
TBX2	-3,248230506	1,262767739	-4,395560384	0,000114081	0,002223576	0,616564462
PAG1	-2,023233033	2,388538691	-4,395374095	0,000114142	0,002223576	0,616043562
NPR2	-1,628658183	2,325593572	-4,393915741	0,00011462	0,002229293	0,611965908
GPR133	-1,522858921	-1,237592542	-4,393688029	0,000114694	0,002229293	0,611329239
LRRK2	-2,355626209	0,245469405	-4,384569534	0,000117728	0,002285674	0,585840994
EMB	-3,346281825	1,438979488	-4,379912955	0,000119307	0,002313731	0,572829733
CCL11	-2,097900729	-1,058797968	-4,375189	0,000120931	0,002337892	0,559633632
REN	-2,508075058	0,787520231	-4,37510394	0,000120961	0,002337892	0,559396054
VSTM4	-2,54361986	0,412000747	-4,368421697	0,000123295	0,002380344	0,540735603
CAMK1G	-1,624636778	-0,511667428	-4,363555839	0,000125023	0,002408304	0,527151875
AFF3	-3,096466274	1,098826927	-4,345793624	0,000131536	0,002521159	0,477597894

GPRASP1	-1,814479229	1,418008111	-4,345586314	0,000131614	0,002521159	0,477019827
F10	-1,822025334	-0,710019858	-4,335134955	0,000135603	0,002591816	0,447885974
PTPRB	-2,42740156	2,656515094	-4,328647978	0,000138139	0,00263736	0,429811987
CD1E	-1,569103509	-1,287386241	-4,319958381	0,00014161	0,002700623	0,40561183
TBXAS1	-2,007161113	1,657263143	-4,288344152	0,000154976	0,002948996	0,317673223
CD5	-2,636396928	0,477336533	-4,286641819	0,00015573	0,002960072	0,31294276
PROCR	-2,106961835	1,951558214	-4,282452159	0,000157601	0,002992333	0,301302584
CEACAM21	-2,58876643	-0,175778143	-4,277890684	0,000159663	0,00302815	0,288632804
TSPAN2	-2,65609435	-0,010515493	-4,277385992	0,000159893	0,003029144	0,287231211
MCAM	-2,03625802	4,469544064	-4,274730135	0,000161108	0,003042144	0,279856279
POSTN	-3,914892393	1,973199203	-4,261719644	0,00016719	0,003150095	0,243745643
TNFSF14	-2,287610188	0,439889682	-4,259073726	0,000168455	0,00317045	0,236405498
FFAR2	-2,155579773	-0,840391818	-4,255191944	0,000170327	0,003202184	0,225639111
FOXN4	-2,615989681	0,247609676	-4,253734782	0,000171035	0,003208489	0,221598255
KCNE4	-2,495631901	0,413014973	-4,246447828	0,000174619	0,003265059	0,201396422
C3orf80	-1,640742232	-1,116095216	-4,238524758	0,0001786	0,003332262	0,179441746
CLEC4E	-2,658941913	0,267275412	-4,237882061	0,000178927	0,003334747	0,177661335
PMP22	-1,985690771	3,374107886	-4,22285624	0,000186738	0,003469059	0,136057669
RGS2	-2,760662388	3,021634503	-4,214202824	0,000191388	0,003547778	0,112116532
GAS1	-2,707459125	0,875237597	-4,202531037	0,00019784	0,003651657	0,079846275
GBP2	-2,686704994	4,167089768	-4,190325881	0,000204815	0,003770802	0,046128306
FAM19A5	-2,766291991	0,469262161	-4,19009129	0,000204952	0,003770802	0,045480496
GBGT1	-1,993942978	0,383273707	-4,187011429	0,000206751	0,003791752	0,036976596
HNRNPUL2<br>-BSCL2	3,455681	4,259850025	4,182016559	0,000209702	0,0038377	0,023188894
LILRA3	-1,757726623	-1,228885022	-4,179004253	0,000211502	0,003862426	0,014876081
PDZD4	-1,807106019	-0,742003234	-4,174671478	0,000214117	0,003906043	0,002922286
PTGIR	-1,70991317	-0,601945906	-4,168862896	0,000217673	0,003962517	-0,013097549
LGALS2	-2,069485839	-0,836771934	-4,168123812	0,000218129	0,003962601	-0,015135451
NEXN	-1,672992801	1,182163521	-4,167661899	0,000218415	0,003963463	-0,01640905
PDE4B	-2,704204713	2,024778484	-4,164239017	0,000220545	0,003997896	-0,025845419
NID1	-3,292257469	3,272673475	-4,154788751	0,000226531	0,004097783	-0,051886694
ITGA1	-2,515724915	3,422592564	-4,153188358	0,00022756	0,004112086	-0,05629505
LAX1	-1,800776471	-0,484582347	-4,148723426	0,000230457	0,004155703	-0,068591286
FCRLA	-1,708488565	-1,232148795	-4,143119898	0,000234143	0,00421466	-0,084017679
GIMAP2	-2,374728456	1,284072835	-4,143010198	0,000234216	0,00421466	-0,084319619
FMNL1	-1,606967968	2,740389623	-4,139349761	0,000236656	0,004254121	-0,094393328
HHEX	-1,877638131	0,644203706	-4,13607787	0,000238858	0,00428476	-0,103395515
FLRT2	-2,432057218	0,811319585	-4,129281596	0,000243496	0,004363423	-0,122087848
CD180	-1,928864517	0,640702851	-4,122695119	0,000248076	0,004431656	-0,140194426
GJA4	-3,22361641	1,244184789	-4,112599531	0,00025526	0,004544057	-0,167930942
NAP1L3	-1,824361909	-0,193894741	-4,112373194	0,000255423	0,004544057	-0,168552542
HEG1	-1,812877287	3,696190634	-4,099715138	0,000264726	0,004690169	-0,203299584
AOC3	-1,881726375	1,708841854	-4,091017209	0,00027131	0,004792027	-0,227156986
ZCCHC24	-1,71456728	2,345493767	-4,086669571	0,000274661	0,004846242	-0,239076242
BMP8A	-2,187901699	0,913056229	-4,081769607	0,000278486	0,004908707	-0,252505055
WT1	-2,295683189	0,265862708	-4,067711636	0,000289753	0,005096869	-0,291004619
POPDC2	-1,539484804	0,436224726	-4,066567392	0,000290689	0,005108124	-0,294136462
FAM49A	-2,020185599	1,700394839	-4,065981214	0,00029117	0,005111359	-0,295740751
GRAP	-2,126445917	2,016497592	-4,063272015	0,000293403	0,005140075	-0,303154516
COL4A1	-2,087242423	7,049041527	-4,046931747	0,00030723	0,005344248	-0,347837054
OLFML3	-2,485078533	2,653599474	-4,045865219	0,000308154	0,005354918	-0,350751507
ZNF334	-2,179073756	-0,576739841	-4,040102635	0,000313196	0,005437043	-0,366494461
TRO	-2,899886719	0,958708888	-4,039528561	0,000313703	0,005440354	-0,368062398

RASL12	-2,681347168	1,511172316	-4,03699643	0,000315947	0,005468264	-0,374977429
NNMT	-3,486199435	2,845152493	-4,032168904	0,00032027	0,005537512	-0,38815716
ARMCX1	-2,332653032	1,355994169	-4,030442462	0,000321829	0,005558902	-0,392869334
PLA2G7	-2,762675683	1,778128495	-4,029049415	0,000323094	0,005575144	-0,396671064
RAB31	-2,517417068	3,958557155	-4,021713689	0,000329831	0,005680016	-0,416683867
KIAA0125	-1,882725968	-1,166385349	-4,02014672	0,000331288	0,005699405	-0,42095724
MSRB3	-2,455715971	1,767295056	-4,017928016	0,000333361	0,005723642	-0,427007082
CDH5	-2,596039199	3,088123949	-4,015574196	0,000335575	0,005755913	-0,433424175
SIRPB2	-1,700901607	-0,622434034	-4,012355165	0,000338626	0,005802462	-0,442198076
VAMP5	-1,907163729	2,532773889	-4,007942574	0,000342852	0,005857393	-0,454221484
SYNE1	-2,198419525	2,621058356	-4,003300144	0,000347354	0,005928425	-0,466866516
CXCR7	-2,335492159	2,87210462	-4,002554631	0,000348082	0,005934974	-0,468896695
USHBP1	-2,030555268	0,361219416	-3,986496121	0,000364138	0,006178156	-0,512597175
RSPO1	-1,598994721	-1,287825939	-3,984239057	0,000366453	0,006211298	-0,51873476
ABCA6	-1,688614791	-0,507617416	-3,978543198	0,000372356	0,006292786	-0,534218275
STON1	-1,821816284	1,111284925	-3,976563677	0,00037443	0,006321625	-0,539597651
CHIT1	-1,824143886	-0,734672269	-3,975731004	0,000375305	0,006330201	-0,541860187
LOC339524	-1,520473884	-0,053391677	-3,96473842	0,000387054	0,006509234	-0,571714392
CYSLTR1	-1,620621382	-0,819484754	-3,961047958	0,000391078	0,006564092	-0,581730941
DNM3OS	-2,633551994	0,201084331	-3,958413774	0,000393976	0,006595208	-0,588878655
DPYD	-2,38654711	1,283988288	-3,95581459	0,000396855	0,006633893	-0,595929834
DYX1C1-CCPG1	2,903441206	1,317529521	3,955537926	0,000397163	0,006633893	-0,596680289
VEGFC	-2,099543959	1,753607525	-3,953156012	0,000399822	0,006665375	-0,603140528
KCNAB2	-2,028442565	2,212127707	-3,945790201	0,000408155	0,006758494	-0,623109804
TBC1D3	-1,955381303	-0,569044537	-3,943306806	0,000411002	0,006792583	-0,629839635
CALHM2	-2,09727379	1,247717295	-3,938148944	0,000416979	0,006858444	-0,643812493
CX3CR1	-1,614316274	-0,277367025	-3,936724946	0,000418644	0,006879258	-0,647669066
DOC2A	-2,081105387	-0,233713147	-3,933436389	0,000422513	0,006936227	-0,656573552
MYOCD	-1,539523719	-1,157199854	-3,913166785	0,000447148	0,00730126	-0,711401534
NDNF	-2,348397926	-0,0764548	-3,906705495	0,000455292	0,007402775	-0,728858347
DPT	-2,027388832	-0,832161525	-3,905312635	0,000457066	0,007418604	-0,732620197
ELFN1	-1,657798962	-0,690852722	-3,904484574	0,000458124	0,007421788	-0,734856413
C22orf34	-1,599525626	-0,880119095	-3,899448963	0,000464611	0,007519792	-0,748451742
CLDN5	-2,583100383	0,897647512	-3,895217882	0,00047013	0,007594844	-0,759870231
C1R	-2,417632223	5,289043893	-3,89023285	0,000476714	0,007694002	-0,77331785
LAMA2	-2,283708396	1,110031985	-3,884815639	0,000483972	0,007792033	-0,787924449
ECEL1P2	-2,114604366	-0,413945269	-3,884687039	0,000484146	0,007792033	-0,788271109
SELL	-2,321343149	1,348646431	-3,883522783	0,00048572	0,007810072	-0,791409356
P2RX1	-1,790272436	-0,713200735	-3,88129027	0,000488753	0,007840675	-0,797426158
RARB	-1,834024979	1,927523195	-3,878673058	0,000492332	0,007886934	-0,804478198
CLECL1	-1,649626981	-1,186894428	-3,877900327	0,000493394	0,007892868	-0,806559988
PSD	-1,958612861	1,32691065	-3,877736393	0,000493619	0,007892868	-0,807001619
CHRDL1	-1,98691879	-0,843833265	-3,871891785	0,000501725	0,008007609	-0,822742395
P2RX5	-1,547916866	-0,775272885	-3,868349072	0,000506702	0,008072072	-0,832279567
IGFBP7	-2,537147019	7,04986851	-3,867279961	0,000508213	0,008088664	-0,835157059
COL11A1	-1,94257509	-0,967281508	-3,865765018	0,000510362	0,008107951	-0,839234017
LAMA4	-2,740807896	3,802310013	-3,865761854	0,000510366	0,008107951	-0,83924253
PODN	-2,098889546	0,561119259	-3,857524573	0,000522207	0,008273161	-0,861400379
LAIR2	-1,504147745	-1,002446993	-3,855534742	0,000525107	0,008311462	-0,866750378
HLA-DQB1	-3,511017455	4,848828708	-3,847473941	0,000537018	0,008476607	-0,888413053
FRZB	-2,619601494	2,373429655	-3,841973483	0,000545296	0,008588928	-0,903185638
IL1B	-2,841189005	0,824895531	-3,83693385	0,000552989	0,008665174	-0,91671386
GZMM	-1,607704687	-0,688875438	-3,831318181	0,000561687	0,008785475	-0,931780768

MAFB	-1,538043092	3,424863011	-3,814212435	0,00058901	0,009179496	-0,977625913
FMO2	-1,685924068	-0,718956463	-3,81068041	0,000594811	0,009244803	-0,9870827
EBF1	-2,178006696	1,133848704	-3,81035391	0,00059535	0,009244837	-0,98795672
KANK3	-1,635328547	0,121788613	-3,808676117	0,000598128	0,009279599	-0,992447636
BATF3	-1,648720243	-0,159259161	-3,805137259	0,000604027	0,009354277	-1,001917626
CD36	-2,444667899	0,242060025	-3,804517687	0,000605066	0,009356284	-1,003575265
CCDC80	-2,275871991	4,053336594	-3,800298442	0,000612186	0,009446649	-1,014861021
ADAM8	-1,968539151	2,891876118	-3,797339725	0,000617228	0,009515919	-1,022772305
NOS3	-1,984152324	1,6934063	-3,790507091	0,000629025	0,009671827	-1,04103323
HLA-DRB5	-3,647378839	3,460548098	-3,7900844	0,000629762	0,009674522	-1,042162513
TXLNB	-1,549344832	-0,847055704	-3,788286478	0,000632907	0,009714159	-1,046965404
PEAR1	-1,919770644	0,520964974	-3,775580088	0,000655568	0,010008451	-1,080884292
ITGA4	-2,708694666	2,351411349	-3,771728721	0,000662591	0,010097771	-1,09115678
NFASC	-2,72565018	0,805923285	-3,769788216	0,000666157	0,010134185	-1,096331054
ADRA2C	-2,563618383	1,646488136	-3,766007167	0,00067316	0,010231678	-1,106410164
ADAMTS6	-2,171689384	1,753110714	-3,764928071	0,000675172	0,010244171	-1,109285995
SLC37A2	-2,301604365	0,690994861	-3,762378923	0,000679947	0,010307545	-1,116078329
MXRA8	-2,772270677	3,067936602	-3,744886993	0,000713613	0,010751643	-1,162639045
HOXD3	-1,697573196	-0,187417898	-3,740633987	0,00072204	0,010868427	-1,173947279
CD3G	-1,987173791	-0,669837104	-3,735638708	0,000732062	0,010991142	-1,1872228
RSPO3	-2,155430439	0,28666378	-3,735175231	0,000732999	0,010995619	-1,188454195
UQCRBP1	-1,937233069	4,761194795	-3,720569343	0,000763121	0,011378204	-1,227229664
LTBP2	-2,449920762	3,990785368	-3,715464692	0,000773929	0,01149186	-1,240767449
HLA-DRB1	-3,648277965	5,65146687	-3,712352168	0,000780593	0,011558545	-1,249018445
RBP5	-1,836054264	0,460219099	-3,688306442	0,000833982	0,012190187	-1,312669442
TBX3	-2,97009693	2,315408815	-3,685458207	0,000840536	0,012256447	-1,320198096
EFEMP1	-3,482843439	2,247641108	-3,68382648	0,000844313	0,012290712	-1,324510146
CRISPLD2	-1,520040344	3,257419	-3,678191245	0,000857485	0,012461391	-1,339396131
MEIS3	-2,06440579	0,857959846	-3,674435184	0,000866375	0,012571618	-1,349313043
PDE2A	-1,779500049	-0,274362883	-3,67366769	0,000868203	0,012585283	-1,351338916
JPH4	-1,711222183	-0,957704161	-3,671766528	0,000872746	0,012629877	-1,356356479
GLI1	-1,966941811	0,168083613	-3,665704185	0,000887387	0,012809467	-1,372349293
DLC1	-2,37711789	2,996802894	-3,664099987	0,000891301	0,012844433	-1,376579479
HIST1H4J	-2,555259658	1,031935864	-3,66348655	0,000892802	0,012850171	-1,378196886
SDPR	-2,47398681	0,905024824	-3,662453722	0,000895335	0,012859524	-1,380919822
HHIP	-2,552919509	0,269964176	-3,661053735	0,00089878	0,01289824	-1,384610235
JAKMIP1	-1,695490423	-0,684765766	-3,647286247	0,000933346	0,01333871	-1,420871369
BTN3A1	-1,718179016	4,767524383	-3,644516577	0,000940454	0,013384752	-1,428159483
HHIP-AS1	-1,839852301	-0,054025199	-3,64394681	0,000941922	0,013394585	-1,42965849
AGBL2	-1,776373965	0,7051621	-3,642736871	0,000945049	0,013427954	-1,432841416
MS4A2	-1,586828074	-1,291520208	-3,640903866	0,000949804	0,013471017	-1,437662585
GLT8D2	-2,06016604	1,353815095	-3,640664713	0,000950426	0,013471017	-1,438291532
PILRA	-1,954751244	1,697862901	-3,63965577	0,000953055	0,013497171	-1,440944761
ZIK1	-1,533761824	-0,816284102	-3,636733402	0,00096071	0,013594398	-1,448628045
DKFZp686 D0853	-2,616703046	-0,758156066	-3,635345395	0,000964366	0,013597931	-1,452276411
LRRC36	-1,523372172	-0,162571588	-3,63175153	0,000973897	0,013668684	-1,461720202
NAIP	-1,525872661	4,325891741	-3,629504432	0,000979902	0,013730588	-1,467623074
INSR	-2,320381771	3,85276552	-3,625554568	0,000990545	0,013834692	-1,477995279
CCL3L3	-1,767776937	-1,12816919	-3,616575245	0,001015158	0,014155498	-1,501557324
FPR2	-1,648773467	-1,189666008	-3,610998496	0,001030742	0,014326397	-1,516178724
PLAT	-2,615501621	3,666157357	-3,604434103	0,001049382	0,014573721	-1,533377561
CXCL10	-2,835018629	2,891803924	-3,603915481	0,001050869	0,014582606	-1,534735805
F2R	-1,796842465	3,601797186	-3,598690215	0,00106596	0,014716564	-1,548415954

PTPRO	-2,278646712	-0,037978395	-3,598503221	0,001066504	0,014716564	-1,548905364
HCG27	-1,661829923	-0,1012482	-3,595290549	0,001075892	0,014822344	-1,557312084
KIF19	-1,999582387	0,228075769	-3,59404636	0,001079549	0,014860837	-1,560566955
MCOLN2	-1,9327693	0,644771476	-3,585948132	0,001103647	0,015132085	-1,581740828
MATK	-1,924802873	-0,475596329	-3,582664009	0,001113567	0,015228925	-1,590321866
GPR75-ASB3	2,03971275	0,889628482	3,579887693	0,001122021	0,015323011	-1,597573472
CCR7	-1,863153246	-0,159200764	-3,572890186	0,001143604	0,01556838	-1,615840087
CXCL11	-2,41161729	0,758669626	-3,571779636	0,001147066	0,015603177	-1,618737724
ATP8A1	-1,573397173	2,709951594	-3,570366145	0,001151487	0,015638612	-1,62242524
TCF4	-3,0637317	4,219685605	-3,56833482	0,00115787	0,015700531	-1,627723475
PCDH18	-2,828633813	2,36641591	-3,563559337	0,00117301	0,015843451	-1,640174149
PRELP	-2,344721206	1,166469369	-3,560158847	0,001183907	0,015934505	-1,649035598
IRF8	-2,92051014	1,569307419	-3,558861738	0,001188089	0,015971967	-1,652414826
CXCR1	-1,779924284	-0,995653881	-3,558026224	0,001190791	0,015995799	-1,654591227
MIR155HG	-1,840055059	0,458803045	-3,555458802	0,00119913	0,016095262	-1,661277649
GLIPR2	-2,513732233	3,350364126	-3,546350074	0,001229173	0,016396811	-1,684983106
FXYD5	-2,31955883	3,000909553	-3,543361635	0,001239188	0,016517068	-1,692754818
TWIST2	-1,643776659	-0,43603071	-3,543028724	0,001240308	0,016519217	-1,69362041
NBPF10	1,529304208	3,978378819	3,53473698	0,001268533	0,016804162	-1,715168178
PRR16	-1,678391381	0,045452262	-3,525705073	0,001299984	0,01708933	-1,738614511
MIAT	-3,411844712	1,899023206	-3,524613462	0,001303836	0,017113837	-1,741446507
NR3C1	-2,526894603	2,076980461	-3,523453561	0,001307941	0,017142205	-1,744455252
S1PR3	-2,280369418	2,66331468	-3,514841158	0,001338812	0,017479673	-1,76678202
P4HA3	-1,550573636	0,309608035	-3,509938765	0,001356699	0,017641377	-1,779480293
GAS6	-1,864615934	2,912337333	-3,50610473	0,001370847	0,017776628	-1,789405865
RMRP	-1,653762001	0,45918908	-3,505272051	0,001373939	0,017803313	-1,791560879
AKAP2	-1,715532018	3,36790379	-3,501794708	0,001386923	0,017931078	-1,800557977
C4A	-2,462877524	3,993683683	-3,499140317	0,001396913	0,018033167	-1,807423166
DTHD1	-1,614656084	-0,470135481	-3,496311386	0,001407637	0,018112931	-1,814737244
PI15	-2,113002589	-0,101238719	-3,494043609	0,001416291	0,018174334	-1,820598597
ANKRD36BP2	-1,547019104	-0,926593611	-3,492149379	0,001423559	0,018240414	-1,825493179
SOCS1	-1,550160302	1,49125121	-3,489134561	0,001435201	0,018321423	-1,833280873
LOC339803	-2,101614502	-0,193795601	-3,486775398	0,001444375	0,018417036	-1,83937284
SOWAHB	1,657570381	1,756377201	3,48665883	0,00144483	0,018417036	-1,8396738
ST6GAL2	-2,031729726	0,266177808	-3,483411379	0,001457555	0,018542937	-1,848056436
VAT1L	-1,588757875	-1,213227368	-3,48331416	0,001457937	0,018542937	-1,848307334
ITGB2-AS1	-1,94789388	0,459820364	-3,482532472	0,001461017	0,018568394	-1,850324559
GPR68	-1,604717883	0,154621891	-3,480279561	0,001469928	0,018648333	-1,856137294
RASD2	-1,634268283	-0,017187652	-3,480121541	0,001470556	0,018648333	-1,856544939
SFRP1	-3,509111253	2,499387248	-3,477661491	0,00148035	0,018744935	-1,862890073
RPPH1	-1,560828661	-0,243896508	-3,477262336	0,001481945	0,018751346	-1,863919411
GLIPR1	-1,937555826	2,887663513	-3,471302212	0,001505962	0,018916249	-1,879283082
DSC2	1,663089531	6,274510924	3,469642822	0,001512715	0,018987226	-1,883558466
ENTPD1	-2,181192626	3,655440327	-3,463957394	0,001536075	0,019211092	-1,898199896
C22orf15	-1,848271609	-0,604257329	-3,454608996	0,001575243	0,019600626	-1,922250921
HLA-DRA	-3,785108043	7,014979234	-3,453074909	0,001581762	0,019667528	-1,926194934
ANO1	-2,050924365	5,086539269	-3,451042208	0,00159044	0,019761157	-1,931419619
ABI3BP	-3,06357902	1,69090982	-3,449376078	0,001597587	0,019821354	-1,935701064
EMP3	-1,754660676	3,187141464	-3,443324917	0,001623804	0,020059925	-1,951242819
GATA3	-1,525278282	-0,08832281	-3,429806421	0,001683881	0,020639229	-1,985918745
SNAP25	-1,728727522	-0,345672124	-3,422940965	0,001715205	0,020993284	-2,003505265
OLFM1	-2,244684303	0,120032829	-3,4183799	0,001736325	0,021176523	-2,015179925

HLA-DOA	-2,5762224	2,01713872	-3,41278141	0,00176259	0,021421008	-2,029500223
CD82	1,611893371	4,902969885	3,402849184	0,001810131	0,021859938	-2,054879042
DACT1	-2,246880336	0,900376501	-3,39826481	0,001832488	0,022026296	-2,066581469
LOC100507463	-1,90256245	2,68264559	-3,397393698	0,001836767	0,022036744	-2,068804308
SUSD3	-1,76409106	0,584611981	-3,388232605	0,001882344	0,022408941	-2,092164843
LGI4	-1,582644649	-0,793821315	-3,388047362	0,001883276	0,022408941	-2,092636902
BNC2	-2,120629869	0,817008718	-3,386732541	0,001889909	0,022461098	-2,09598716
MDFIC	-2,181519266	2,043394018	-3,375586505	0,001947049	0,023013127	-2,124363579
CHST11	-2,289538961	2,817464035	-3,369867168	0,001977009	0,023306084	-2,13890724
PDE1A	-1,603879139	1,186836821	-3,368307651	0,001985255	0,023361224	-2,142870908
THBS4	-2,26708131	2,0587246	-3,360891212	0,002024925	0,023705796	-2,161708676
PATL2	-1,555256961	-0,149318595	-3,359661344	0,002031576	0,023751372	-2,164830644
LRRC4B	-1,809485264	-0,727652914	-3,353094472	0,002067448	0,024056438	-2,1814912
CD38	-1,647752005	0,580512646	-3,346986431	0,00210136	0,024396762	-2,196973766
HCLS1	-2,499929906	3,552865569	-3,34680714	0,002102363	0,024396762	-2,197428028
CBFA2T3	-1,527602141	-0,298500451	-3,345884885	0,002107532	0,024440274	-2,199764517
LCP1	-2,28918747	5,256768645	-3,339729334	0,002142345	0,024770984	-2,215351444
CCL2	-2,404181555	3,62392268	-3,339700681	0,002142508	0,024770984	-2,215423966
SCG5	-2,161500297	0,738584915	-3,338545142	0,002149105	0,024805452	-2,218348451
MUSTN1	-1,667394205	0,027214685	-3,331573326	0,002189322	0,025168431	-2,235982714
TTYH2	-1,632813747	0,650808505	-3,330548884	0,002195292	0,025220226	-2,238572409
NLRP1	-2,435369581	1,885944138	-3,328880893	0,002205046	0,025315393	-2,242788121
CCDC17	-1,518189296	1,304851884	-3,32846445	0,002207487	0,02532654	-2,243840489
RBMS3	-2,004680212	1,286124483	-3,316080529	0,002281293	0,025914344	-2,275106123
PTPN22	-2,201845587	0,674647274	-3,314779012	0,002289186	0,025971034	-2,278388789
FAM92B	-2,225898545	0,563157213	-3,314512074	0,002290808	0,025971034	-2,279061979
LOC389895	-1,954690148	-0,163779903	-3,309167979	0,002323513	0,026289898	-2,292533697
C19orf59	-1,598168575	-1,146494123	-3,3079779	0,002330857	0,026355677	-2,295532287
SLC15A3	-2,314338031	2,516455295	-3,299481871	0,002383936	0,026780018	-2,316924103
SPIB	-1,507535393	-0,41855862	-3,292761689	0,002426739	0,027129095	-2,333825578
CPXM2	-2,571619325	0,945599644	-3,29262556	0,002427614	0,027129095	-2,334167773
FBN1	-1,992667898	3,53041767	-3,289278047	0,002449216	0,027317326	-2,342580419
RUNDC3B	-1,802657201	-0,231132468	-3,285740819	0,002472243	0,027485149	-2,351465281
HOXD8	-2,408315018	1,443796434	-3,280735291	0,002505183	0,027779622	-2,364030218
CSPG4	-1,931268231	2,049615418	-3,277114773	0,00252927	0,027974665	-2,373112612
LINC00839	-1,843856671	-0,353432419	-3,275694415	0,00253878	0,028008024	-2,376674357
OGN	-1,947581285	-1,13395769	-3,268461026	0,002587744	0,028426841	-2,394801263
INS-IGF2	-2,387878471	-0,583717324	-3,266130689	0,00260371	0,02857775	-2,400636878
MAN1C1	-1,886677782	1,450324283	-3,264475789	0,002615106	0,028684543	-2,404779816
ITGA5	-2,581312426	4,162170694	-3,264142724	0,002617405	0,028691488	-2,405613497
TCF21	-1,572373915	-1,321561375	-3,263847538	0,002619444	0,028695578	-2,40635233
IL22RA2	-1,514339642	-1,350578512	-3,261103593	0,002638474	0,028830689	-2,413218673
ASB2	-1,774139214	1,260712719	-3,249092288	0,002723344	0,029626478	-2,443241481
CPNE5	-1,7159364	0,14805933	-3,248795988	0,002725471	0,02963089	-2,443981398
CACNA1C	-1,577440544	1,257402815	-3,243632835	0,002762777	0,02988675	-2,456869359
LOC100144604	-2,252982497	1,149093354	-3,231187903	0,002854716	0,030629951	-2,487891414
CHRDL2	-1,688353193	-1,027238179	-3,227854564	0,002879834	0,030871464	-2,496190396
ZNF521	-2,439450006	1,018732316	-3,220414689	0,00293666	0,031344183	-2,514697788
LPAR6	-2,018102844	3,662733035	-3,218268987	0,002953247	0,031471449	-2,520031412
CAV1	-2,010108617	3,687330034	-3,213812145	0,002987986	0,031763026	-2,531104136
TMOD2	-1,791327564	0,722683913	-3,206000434	0,003049818	0,032320561	-2,550493003
XCL1	-2,161183611	0,640973235	-3,201671286	0,003084608	0,032649072	-2,561227709

OLFML2A	-1,981899528	2,193441858	-3,193111487	0,003154516	0,033286717	-2,582431072
GBP1	-2,226246019	4,301217636	-3,192605451	0,003158696	0,033310412	-2,583683658
PPP1R14A	-1,741661031	1,392456464	-3,18728216	0,003202987	0,033623652	-2,596854173
GPR132	-1,500419534	0,301486689	-3,184163515	0,003229209	0,033805599	-2,604564863
SOCS3	-2,064134149	4,540138557	-3,177885608	0,003282618	0,034239828	-2,62007488
ISY1-RAB43	2,053194161	4,703199041	3,173751289	0,003318249	0,034548705	-2,630280388
NDUFA4L2	-2,528492768	2,458345799	-3,171018175	0,003342006	0,034775032	-2,637023271
GBP1P1	-1,561141907	-0,471393813	-3,16400073	0,003403748	0,035310812	-2,654322271
INPP5D	-2,038317157	2,794122065	-3,153540481	0,003497804	0,03598308	-2,680071303
C10orf54	-1,933429357	2,982851356	-3,150790625	0,003522937	0,036114366	-2,686832993
C2orf74	-1,757924756	0,892958096	-3,150767652	0,003523148	0,036114366	-2,68688947
PSMB9	-2,255277754	4,043009917	-3,144602921	0,003580124	0,036589504	-2,702036827
C1orf228	-1,548204872	1,08030149	-3,131000423	0,00370896	0,037593883	-2,73540446
FEZ1	-1,744533853	1,169145338	-3,119693137	0,003819404	0,038374539	-2,763083796
CR1	-1,644393859	0,027683983	-3,113087254	0,003885367	0,038801019	-2,779229943
TLE4	-1,73229336	1,608455767	-3,111251526	0,003903889	0,0388831	-2,783713618
ANGPT1	-1,714371531	-0,367647823	-3,11093442	0,003907097	0,038892558	-2,784487992
SLC24A3	-1,779962657	0,765325965	-3,110506104	0,003911435	0,038913238	-2,785533872
CSDC2	-1,764658893	0,04575656	-3,103232781	0,003985786	0,039515957	-2,80328251
GNG11	-2,446063294	3,955415516	-3,102339335	0,003995011	0,03958463	-2,805461204
STARD9	-1,583889331	1,459315082	-3,093718524	0,004085074	0,040222451	-2,826466083
ANKRD1	1,903648144	0,924675443	3,093076673	0,004091857	0,040266207	-2,828028723
MAF	-2,256795774	2,418267585	-3,092083564	0,004102371	0,040323595	-2,830446187
CUBN	-1,842163716	1,631509965	-3,086957393	0,004157056	0,040690113	-2,842917892
LYPLAL1	-2,408145187	1,361797287	-3,086407138	0,004162966	0,040690113	-2,844255972
EMILIN3	-1,513377559	-0,119053008	-3,083975036	0,004189188	0,040873659	-2,850168695
PAGE4	-1,962631588	-1,126432539	-3,08030801	0,004229021	0,0412156	-2,859078926
CHN1	-1,538351867	2,95803722	-3,064122132	0,004409174	0,0427055	-2,898339376
EHD2	-2,017014859	3,035747639	-3,059147874	0,004465985	0,043110277	-2,910382423
HSD17B3	-1,591716702	0,317167458	-3,05735217	0,004486665	0,043272366	-2,914727345
HLA-H	-1,994350282	4,013863467	-3,057035968	0,004490315	0,043272366	-2,915492289
GFPT2	-1,837141007	0,75392449	-3,055969648	0,004502647	0,043342699	-2,918071577
SERPINE1	-2,358816791	2,691116428	-3,053907088	0,004526592	0,043524536	-2,923059247
SNCAIP	-2,099601133	1,966180476	-3,052634066	0,004541431	0,043594196	-2,926136748
MOXD1	-2,604479107	2,963882075	-3,048209969	0,004593361	0,043921301	-2,936826452
CCDC78	-1,938386609	2,267998486	-3,047782486	0,004598408	0,043941998	-2,937858907
FCRL2	-1,548662287	-0,315010149	-3,046823801	0,004609747	0,044004683	-2,940174035
FAM20A	-2,580689615	2,55905711	-3,041022121	0,004678932	0,044480732	-2,954175998
PLAC9	-1,546763418	-1,259204681	-3,037963161	0,004715806	0,0447438	-2,961552708
FBLN2	-1,748958854	2,366301404	-3,036876163	0,004728975	0,044795205	-2,964173034
ZNF662	-1,78655362	0,413428801	-3,033767751	0,004766827	0,045079365	-2,971663356
GABRB3	-1,505719191	-0,837693175	-3,02476955	0,004878024	0,045901605	-2,993322488
HSPB6	-2,32502395	1,449685522	-3,020235905	0,004934975	0,046237874	-3,004221766
ADGB	-1,507507987	-0,850907499	-3,011781621	0,005042864	0,046977572	-3,02452246
CIITA	-1,581929475	2,395237688	-3,007822177	0,005094156	0,047284428	-3,034019157
LOC100132062	-1,842543857	-0,550528951	-3,00368166	0,005148321	0,047640646	-3,043942736
LOC100132287	-1,842543857	-0,550528951	-3,00368166	0,005148321	0,047640646	-3,043942736
AMY2B	-1,529628105	2,912296695	-3,002906803	0,005158518	0,047658177	-3,04579899
C10orf32-AS3MT	2,299884188	0,303031446	2,995281123	0,005259894	0,048387093	-3,064052916
C15orf48	-2,868190606	1,686441622	-2,989822395	0,005333617	0,048881393	-3,077103801
RGS10	-1,578047696	5,816060454	-2,980929058	0,005455821	0,049692816	-3,098337699

TEKT4	-1,769992417	-0,086448575	-2,97819255	0,005493953	0,049890106	-3,104864295
CD74	-2,285943382	9,234588414	-2,978020317	0,005496361	0,049890106	-3,105274958

Table S5. Genes included in card

ABL1	CTNNB1	FLT3	KIT	NOTCH1	RUNX1
AKT1	EGFR	GATA1	KRAS	NOTCH4	SMAD4
AKT2	ERBB2	GNA11	MAG	NRAS	SMARCB1
AKT3	ERBB3	GNAQ	MAP2K1	PDGFRA	SRC
ALK	ESR1	GNAS	MET	PIK3CA	STK11
APC	FBXW7	HRAS	MLH1	PIK3R1	TP53
BRAF	FGFR1	IDH1	MPL	PIK3R5	VHL
CDH1	FGFR2	IDH2	MSH6	PTEN	
CDKN2A	FGFR3	JAK1	MYC	RB1	
CSF1R	FGFR4	JAK3	NF2	RET	

Table S6. Mutational study

Sample	Only in primary tumor	Only in PDOX	Shared mutations
1	ERBB2_V477L	PIK3CA_R88Q; PTEN_E7X; PTEN_R130Q; RB1_E137X	PTEN_M134I; TP53_R273C
2	TP53_R213X		APC_V1377I; CDKN2A_H83Y; FGFR1_R609X; FGFR1_E612K; FGFR2_V463I; PIK3CA_R88Q; PIK3CA_R108C; PTEN_E7X; PTEN_R130Q; PTEN_F341V; TP53_R342X
3		FBXW7_R465C; FGFR4_P279T	PIK3R1_N564D
4		FGFR3_T394M; TP53_Q165K	KRAS_G13D; PIK3CA_E545G; PTEN_R130G; PTEN_T321fs
5			ERBB3_N62K; PTEN_R130G; PTEN_T321fs
6		CTNNB1_D32N; FGFR1_A21T; MSH6_T1085fs; PIK3R1_T576fs; TP53_Q165K	FGFR2_S252W; PTEN_S229X
7	FGFR2_N228H	FGFR2_S57L; JAK1_D145E; KRAS_Q61H; PIK3CA_E545K; PIK3R1_L466X; PTEN_E7X; RB1_K130T; STK11_Q37X; TP53_K132T	FGFR1_c_1430+7A>C; PIK3CA_E81K
8	STK11_G279fs	FGFR3_c_616-6G>A; NRAS_G12V	FGFR4_E316V; NRAS_G12D
9		PTEN_L182V	CTNNB1_G34R; PIK3CA_E542Q
10		FGFR4_A729V; PTEN_R130G; PTEN_Y177fs	EGFR_M793V; JAK1_K142fs; NOTCH1_L1678M; TP53_R342X
11	CDKN2A_R103Q; HRAS_E63del; STK11_G163D; TP53_R273C		MSH6_T1085fs; PTEN_L265fs; PTEN_T321fs
12	FBXW7_R465C; TP53_R280I	APC_R564X; JAK1_K142fs; TP53_H179R	ERBB2_R678Q; PIK3CA_Y1021C; PTEN_T321fs; TP53_R273C
13	APC_S1400L; CTNNB1_D32G; FGFR4_c_2154-5C>T; PIK3CA_R88Q	FGFR4_E330K; JAK1_D145E; TP53_F341V; TP53_R213X; TP53_D281E	ERBB2_T900S; PTEN_E299X
14			KRAS_G12C; PIK3CA_H1047R; PTEN_L265fs; PTEN_E291fs; STK11_P275L
15	APC_T1292M; FGFR2_R759X; FGFR2_c_940-4C>A; KIT_K558N; PTEN_E299X; TP53_R290C	FBXW7_R465H	TP53_R213X

SUPPORTING FIGURE LEGENDS

Suppl Figure-1

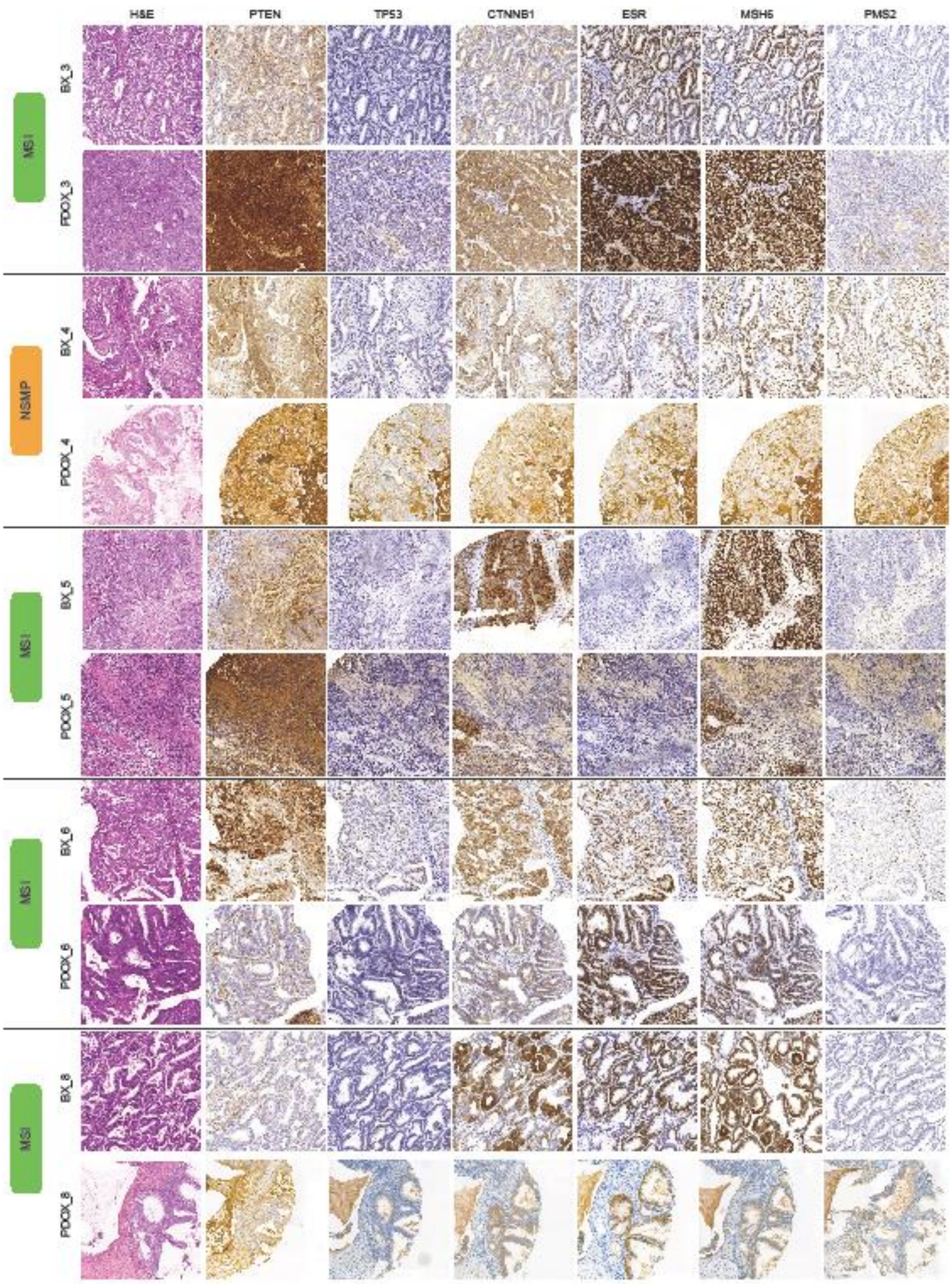

Figure S1. Morphological and immunohistochemical correlation between primary tumors (BXs) and PDOXs. Images illustrate HE stainings and detection by immunohistochemistry of PTEN, TP53, CTNNB1, ESR, MSH6 and PMS2 between BX and PDOX. BX3, BX5, BX6, BX8 (MSI) and BX4 (MSS).

Suppl Figure-2

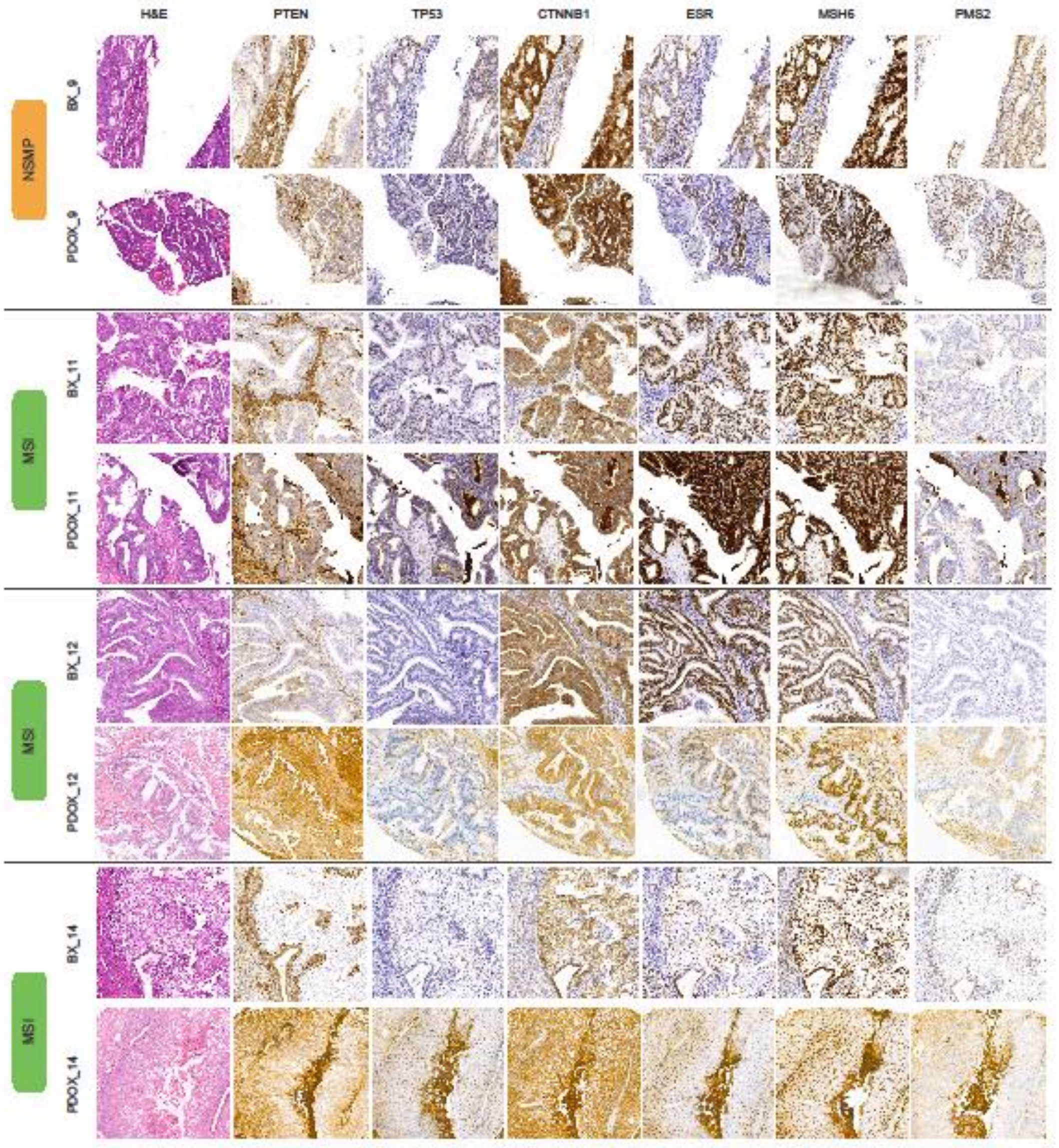

Figure S2. Morphological and immunohistochemical correlation between primary tumors (BXs) and PDOXs. Images illustrate HE stainings and detection by immunohistochemistry of PTEN, TP53, CTNNB1, ESR, MSH6 and PMS2 between BX and PDOX. BX11, BX12, BX14 (MSI) and BX9 (MSS).

Suppl Figure-3

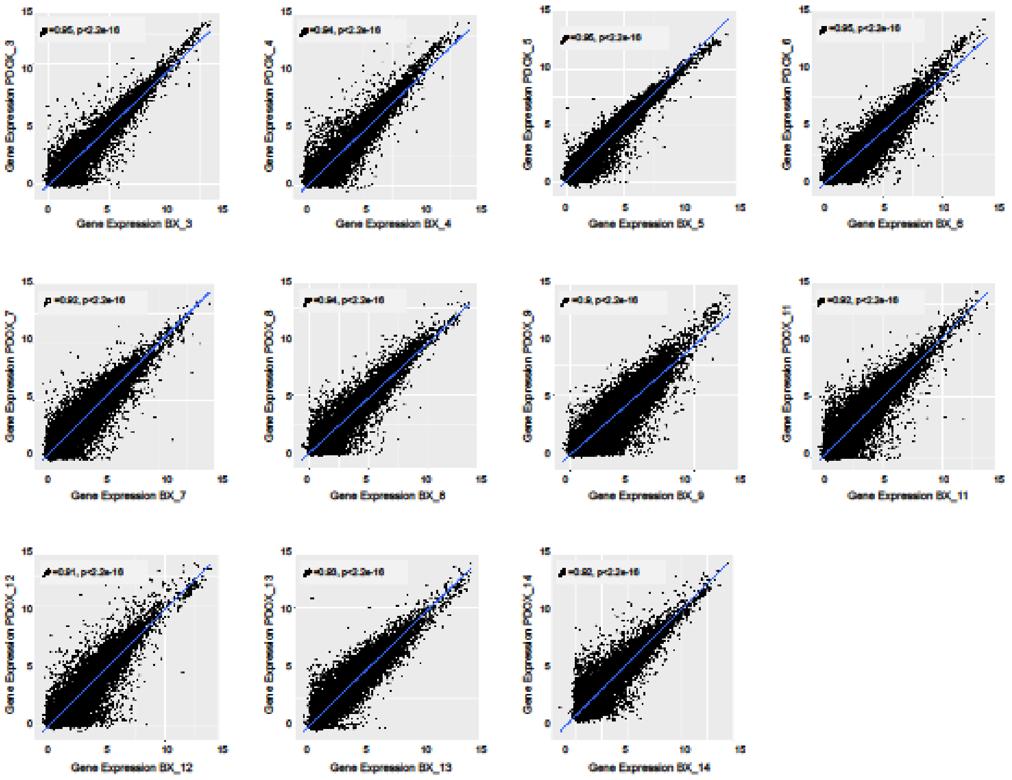

Figure S3. Transcriptomic analysis of BX and PDOX tumor pairs**.** Total RNA sequencing for the 15 pairs of BX/PDOX tumors was conducted and computationally analyzed. BX and PDOX pairs correlated extremely well at transcriptomic level. Spearman's correlation (rho) ρ values and P values indicate the strength and significance of the correlations, respectively.

Suppl Figure-4

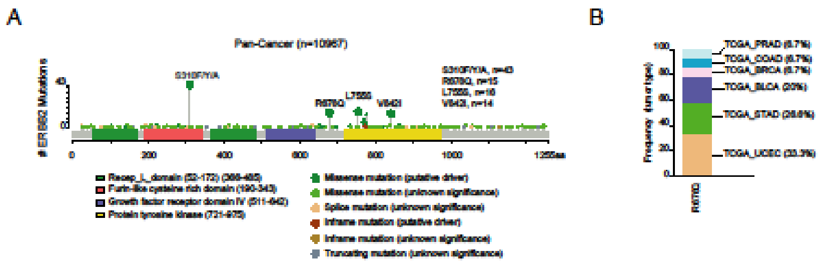

Figure S4. (A) Lollipop plot and analysis of the ERBB2 mutational landscape using the TCGA pancancer dataset (n=10,967) showing that ERBB2^{R678Q} is one of the most representative ERBB2 mutations. **(B)** Stacked bar chart illustrating the distribution of ERBB2^{R678Q} according to tumor types and the highest representation in endometrial cancer (TCGA_ucec).
